# Identification and development of a series of disubstituted piperazines for the treatment of Chagas disease

**DOI:** 10.1016/j.ejmech.2022.114421

**Published:** 2022-08-05

**Authors:** Kate McGonagle, Gary J. Tarver, Juan Cantizani, Ignacio Cotillo, Peter G. Dodd, Liam Ferguson, Ian H. Gilbert, Maria Marco, Tim Miles, Claire Naylor, Maria Osuna-Cabello, Christy Paterson, Kevin D. Read, Erika G. Pinto, Jennifer Riley, Paul Scullion, Yoko Shishikura, Frederick Simeons, Laste Stojanovski, Nina Svensen, John Thomas, Paul G. Wyatt, Pilar Manzano, Manu De Rycker, Michael G. Thomas

**Affiliations:** a Drug Discovery Unit, Wellcome Centre for Anti-Infectives Research, University of Dundee, Dundee, DD1 5EH, UK; b Global Health R&D, GlaxoSmithKline, Tres Cantos, 28760, Spain

## Abstract

Approximately 6–7 million people around the world are estimated to be infected with *Trypanosoma cruzi,* the causative agent of Chagas disease. The current treatments are inadequate and therefore new medical interventions are urgently needed. In this paper we describe the identification of a series of disubstituted piperazines which shows good potency against the target parasite but is hampered by poor metabolic stability. We outline the strategies used to mitigate this issue such as lowering logD, bioisosteric replacements of the metabolically labile piperazine ring and use of plate-based arrays for quick diversity scoping. We discuss the success of these strategies within the context of this series and highlight the challenges faced in phenotypic programs when attempting to improve the pharmacokinetic profile of compounds whilst maintaining potency against the desired target.

## Highlights

• Novel series of *T. cruzi* inhibitors identified.
• Compound **1** had promising rate-of-kill, washout profile and *in vivo* proof of concept.
• Synthesised plate-based arrays and piperazine bioisosteres to expand SAR.
• LogD reduction was a successful strategy to improve metabolic stability.

## Graphical abstract

**Figure:**
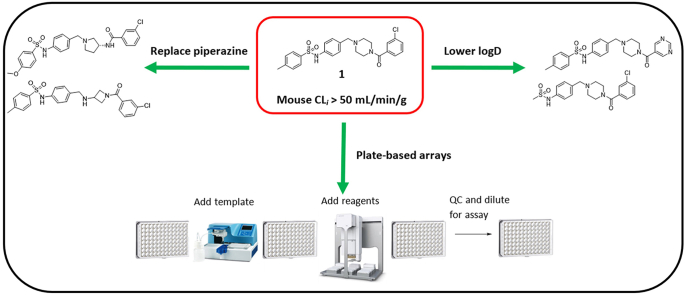

## 1 Introduction

Chagas disease is a parasitic infection which is responsible for around 10,000 deaths per year with 6–7 million people infected and approximately 70 million at risk of infection [[1], [2], [3]]. The disease is most prevalent in Latin America where it is responsible for more deaths than any other parasitic disease. Due to migration, Chagas disease is now also present in many other non-endemic countries [[4], [5], [6]]. The disease is caused by *Trypanosoma cruzi (T. cruzi)* and is primarily spread via an insect vector (triatomine or “kissing bug”) but can also be transmitted congenitally [7], by blood transfusions and through ingestion of contaminated food and juices.

Chagas disease has two clinical phases. The acute phase directly follows infection and presents with non-specific flu-like symptoms including fever and malaise. The chronic asymptomatic or indeterminate phase follows this, with most patients experiencing no symptoms. Around 10–30% of chronically infected patients will progress to the chronic symptomatic phase of the disease. This can occur 10–30 years after the acute infection and involves problems with the heart or gastrointestinal tract which can ultimately be fatal [[8], [9], [10]].

Current treatments for Chagas disease are very limited; the nitroaromatic drugs benznidazole and nifurtimox are the only available options. These drugs suffer from issues such as long treatment duration (60–90 days) and toxicity which frequently leads to premature treatment discontinuation [11,12]. In terms of new chemical entities, two sterol C14*α-*demethylase (CYP51) inhibitors, posaconazole and fosravuconazole, were progressed to the clinic based on promising *in vitro* profiles. The results from these trials were disappointing with treatment failing in 70–90% of patients [13,14].

Despite the disappointing clinical outcome of the CYP51 inhibitor trials, the information gained from these studies has led to significant back-translation of the clinical data which should assist in identifying new candidates with novel mechanisms of action which have a greater chance of success. We have implemented these learnings in our *in vitro* screening cascade, introducing steps to filter out compounds which share the same CYP51 driven mode of action exhibited by posaconazole and fosravuconazole. The drug discovery pathway we have developed includes a CYP51 inhibition assay [15], rate-of-kill determination [16], assessment against a panel of strains and a washout assay, aimed to identify compounds which effectively clear all parasites *in vitro*, with no resurgence when the drug pressure is removed [17,18].

Here we present the discovery and optimisation of a series of disubstituted piperazines which shows efficacy against *T. cruzi*. We reveal a promising *in vitro* profile for the series and discuss the challenges associated with its optimisation.

## 2 Results and discussion

**Fig. 1:**
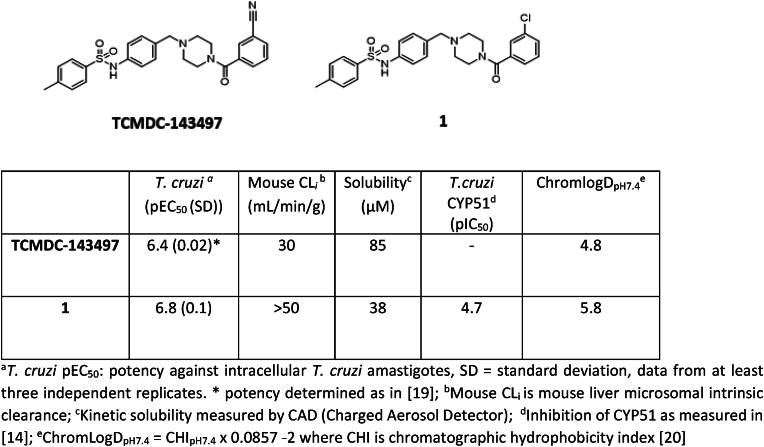
The structure of initial hit compound **TCMDC-143497** and compound 1 with key profiling data.

The starting point for this project was a high-throughput screen (HTS) of the GlaxoSmithKline (GSK) diversity collection of 1.8 million compounds undertaken against *T. cruzi*, cultured in NIH-3T3 fibroblasts. Potent and non-cytotoxic compounds with lead-like properties were selected and clustered into a “Chagas-box” with 222 compounds [19]. The most promising hits were selected for further optimisation based on physchem properties (MW < 500; Ar rings ≤ 4; cLogP <4) and visual inspection (to discard compounds which were similar to known series). One of the hits identified through this HTS and prioritization exercise was TCMDC-143497, with a reported potency of pEC_50_ 6.4 against intracellular *T. cruzi* [19]. A rapid hit expansion approach was performed by selecting available close analogues from the GSK and DDU compound collections (Tanimoto coefficient >0.7) and testing them against *T. cruzi*, resulting in the identification of a more potent analogue, compound **1** (see Fig. 1).

**Fig. 2:**
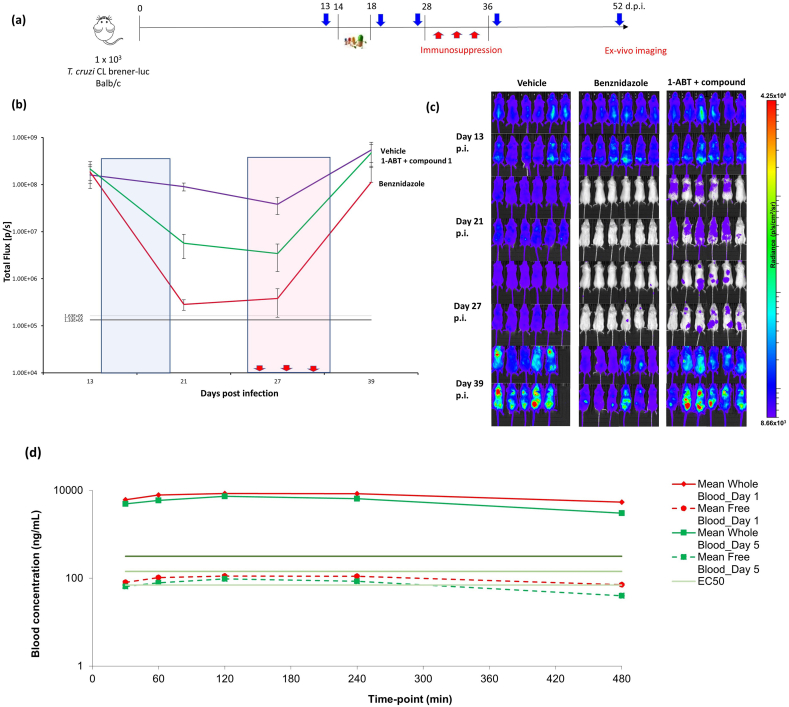
Results of acute *T. cruzi* infection efficacy study. Treatments: vehicle b.i.d. 10 mL/kg for 5 days, benznidazole 50 mg/kg b.i.d. for 5 days and 1-ABT (1-aminobenzotriazole) b.i.d. 50 mg/kg as a pre-treatment 30 min prior to each dose of compound **1** b.i.d. 50 mg/kg for 5 days **(a)** Study outline. Blue arrows indicate imaging days, red arrows indicate immunosuppression days. 5 day dosing begins on day 14 **(b)** Quantification (Total Flux [p/s]) of combined ventral and dorsal bioluminescence for the mice shown in **(c)**. Black line and grey line represent limit of detection of the imaging system and are the mean and mean + 2 SDs, respectively, for infected untreated control mice. Vehicle (purple), benznidazole (red), 1-ABT + compound **1** (green). Blue box indicates dosing period, red box indicates immunosuppression period **(c)** Whole body imaging – dorsal and ventral. Heat-maps are on log_10_ scales and indicate intensity of bioluminescence from low (blue) to high (red), minimum and maximum radiance values as indicated. The mice were immunosuppressed on days 28, 32 and 36 post-infection using cyclophosphamide (200 mg/kg i.p.). Study shows reduction in parasite burden when dosing compound **1** compared to vehicle but relapse is observed post immunosuppression **(d)** Exposure data (n = 3) for compound **1** from efficacy study showing sustained whole blood levels over EC_99_ for the duration of the study on day 1 and day 5. Free blood levels are over or around EC_50_ on day 1 but drop below EC_50_ after 5 h on day 5. (For interpretation of the references to colour in this figure legend, the reader is referred to the Web version of this article.)

Compound **1** demonstrated good potency against intracellular parasites and this activity does not appear to be driven by inhibition of *T. cruzi* CYP51. Rate-of-kill experiments comparing **1** with posaconazole further confirmed a non-CYP51 driven mode of action, as **1** showed a faster kill profile than the characteristically slow profile seen for CYP51 inhibitors [15,16] (Supplementary Information, Fig. S1). We tested **1** in a *T. cruzi* washout assay to assess the compound's ability to clear all parasites *in vitro*. In this experiment, *T. cruzi*, cultured in Vero cells were treated with compound **1** at 20- to 30-fold the EC_50_ for 8 days (with compound being re-supplemented after 4 days). After treatment, the culture medium containing compound **1** was removed, the cell monolayer was washed extensively and then incubated with fresh medium containing no compound **1.** After approximately a further 2 weeks, parasite relapse was observed, indicating that compound **1** could not kill all parasites under these conditions (Table 1). The same result was obtained in three independent experiments (Supplementary Information, Table S1). Compound **1** performed better than posaconazole, which showed earlier relapse at equivalent relative concentrations, but was not as good as benznidazole, which at lower relative concentration tended to relapse later. Despite the poor metabolic stability of **1**, we were keen to demonstrate proof of concept for this compound series in an *in vivo* efficacy study. We therefore ran a 4-day PK study on **1**, dosing at 50 mg/kg twice daily. As the intrinsic clearance was very high, compound **1** was co-dosed with 1-ABT (1-aminobenzotriazole) to inhibit CYP-mediated degradation and maximise exposure. The study showed that the compound gave coverage above EC_50_ for >8 h on day 1, although this did drop slightly by day 4 (Supplementary Information, Fig. S2). As this was a novel series with an unknown mechanism of action, the PKPD drivers were unknown, so this coverage was considered suitable for progression to an animal model of Chagas disease. Compound **1** was dosed twice daily at 50 mg/kg for 5 days in an acute Chagas disease mouse efficacy study. This mouse model, developed by the Kelly laboratory, is able to distinguish between benznidazole (clinically effective) and posaconazole (clinically ineffective) [20]. As in the previous PK study, compound **1** was co-dosed with 1-ABT and showed reduction in parasite burden compared to vehicle, although parasite levels were never reduced below the level of detection. Blood samples were taken on days 1 and 5 and showed that total exposure exceeded the *in vitro* EC_99_ for the duration of dosing. Free blood levels were over or around the EC_50_ on day 1 but dropped below the EC_50_ after 5 h on day 5 (Fig. 2).

**Table 1:** Washout experiment.

Compound	Conc. (μM)	Fold EC_50_	Relapse Day^a^
**1**	3.9	25	11
posaconazole	0.1	25	7
benznidazole	36	12.5	18
benznidazole	144	50	>60

Results for one of three independent washout experiments, others detailed in Supplementary Information (Table S1). T. cruzi infected Vero cells were treated for 8 days at the indicated concentrations (compounds replenished after 4 days), followed by extensive washing to remove compounds and incubation until parasites were observed microscopically.

a day after washout of compounds on which egressed parasites were first detected.

It was possible that increasing the duration of treatment or dose would lead to a further reduction in parasite levels, both in *in vitro* washout and *in vivo* animal model studies, but because compound **1** was hindered by poor solubility and metabolic stability we decided to focus on identifying improved compounds before running further efficacy studies. Three parallel approaches to optimise compound **1** and improve the metabolic stability were adopted. We investigated the terminal amide and sulphonamide moieties to understand the requirement for potency while maintaining or lowering the logD, since high lipophilicity is often correlated to high intrinsic clearance [21,22]. To expand upon this, plate-based arrays were used to rapidly synthesise and screen a large number of compounds without the need for purification. This allowed for a far more rapid make, test process to efficiently assess diverse groups. The third approach was to identify bioisosteric replacements of the embedded piperazine ring which was shown by metabolite identification (metID) studies to be a metabolic liability.

As this was a phenotypic series, with no structural information to guide optimisation, we initially focused on the terminal substituents, making numerous changes to build our understanding of the SAR. We first investigated the optimisation of the terminal sulphonamide whilst maintaining the amide portion of the molecule (Table 2). Truncating to the simple methyl sulphonamide **2** resulted in complete loss of potency suggesting larger substituents are required in this region. This compound did, however, show significant improvement in metabolic stability and solubility, which is consistent with our hypothesis that the high intrinsic clearance may be associated with high lipophilicity. Moving to the unsubstituted aromatic **3** showed improved solubility but suffered from a log unit drop in potency compared to **1**. Replacing the *p*-methyl with a *p*-methoxy substituent **4** resulted in a 0.5 log unit drop in potency whilst showing similar solubility. Switching this substituent for a nitrile **5** led to a considerable loss in potency. The dioxolane compound **6** showed equivalent potency to compound **1** but no marked improvement in solubility or microsomal stability was observed. Finally, removing the aromatic ring and replacing with saturated ring systems containing heteroatoms such as **7**, **8**, and **9** led to improved solubility. However, the intrinsic clearance was still too high and without exception these changes resulted in significant drops in potency.

**Table 2:**
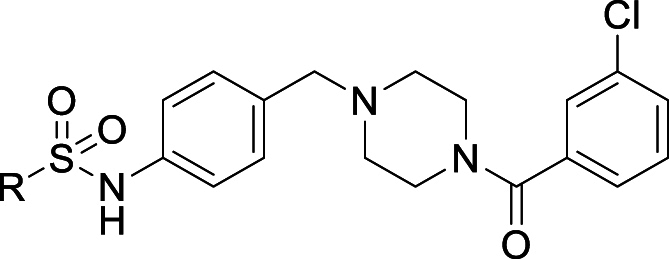
Changes to the sulphonamide substituent of compound **1**.

Structure	Number	*T. cruzi*^a^ (pEC_50_)	Mouse CL_*i*_^b^ (mL/min/g)	Solubility^c^ (μM)	ChromlogD_pH7.4_^d^
	**1**	6.8	>50	38	5.8
	**2**	<4.3	2.6	≥470	4.0
	**3**	5.8	>50	67	5.5
	**4**	6.3	>50	43	5.6
	**5**	5.1	>50	54	5.2
	**6**	6.8	>50	33	5.2
	**7**	4.8	44	486	4.6
	**8**^**1**^	4.6	21	≥402	5.1
	**9**	4.8	32	≥430	4.1

^1^Nitrogen linked directly to aromatic ring of core structure.

a *T. cruzi* pEC_50_: potency against intracellular *T. cruzi* amastigotes, data from at least three independent replicates, standard deviations ≤0.2.

b Cl_i_ is mouse liver microsomal intrinsic clearance.

c Kinetic solubility measured by CAD (Charged Aerosol Detector)

d ChromLogD_pH7.4_ = CHI_pH7.4_ × 0.0857–2 where CHI is chromatographic hydrophobicity index [20].

We then focused on the amide substituent, maintaining the sulphonamide portion of the molecule with the *p-*methoxyphenyl sulphonamide identified in compound **4** (Table 3). Unsubstituted aromatic compound **10** shows almost a log unit drop in potency while demonstrating an improvement in solubility. Substituting chlorine for fluorine **11** led to an equipotent compound with no significant advantage in other properties, similarly with substitution for methyl **12**. Addition of a methoxy or methyl substituent **13**, **14** and **15** led to reduced potency and no improvement in solubility or microsomal stability. We also looked at a series of heterocyclic amides. Placing a nitrogen into the aromatic ring **16** to introduce some polarity and reduce the ChromlogD_pH7.4_ worked to improve solubility and maintain reasonable potency but had no impact on clearance. Moving to other heterocycles such as **17**, **18** and **19** showed promise in terms of solubility as well as microsomal stability but this was at the expense of potency. Various small alkyl groups exemplified by **20**, **21** and **22** were also investigated; many of these compounds exhibited lower ChromlogD_pH7.4_ compared to compound **4** which translated to superior clearance and solubility but significantly reduced potency.

**Table 3:**
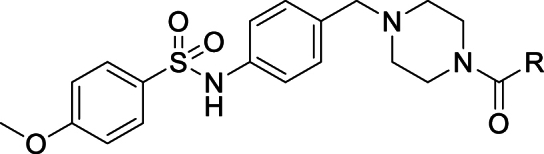
Changes to the amide substituent of compound **4**.

Structure	Number	*T. cruzi*^a^ (pEC_50_)	Mouse CL_*i*_^b^ (mL/min/g)	Solubility^c^ (μM)	ChromlogD_pH7.4_^d^
	**4**	6.3	>50	43	5.6
	**10**	5.5	>50	116	4.6
	**11**	6.2	>50	76	4.8
	**12**	6.0	NA	64	5.1
	**13**	5.5	>50	18	5.1
	**14**	5.7	>50	27	5.2
	**15**	5.9	>50	13	5.8
	**16**	6.0	>50	171	4.3
	**17**	5.2	6.4	122	4.9
	**18**	<4.3	<0.5	≥336	2.3
	**19**	<4.3	12	414	3.2
	**20**	<4.3	1.5	≥526	2.3
	**21**	<4.3	1.0	≥511	2.6
	**22**	<4.3	8.0	≥374	4.3

a *T. cruzi* pEC_50_: potency against intracellular *T. cruzi* amastigotes, data from at least three independent replicates, standard deviations ≤0.2.

b Cl_i_ is mouse liver microsomal intrinsic clearance.

c Kinetic solubility measured by CAD (Charged Aerosol Detector).

d ChromLogD_pH7.4_ = CHI_pH7.4_ × 0.0857–2 where CHI is chromatographic hydrophobicity index [20].

As this initial SAR investigation progressed it clearly highlighted a disconnect between optimisation of potency and optimisation of clearance and solubility. We decided to employ plate-based array chemistry [[23], [24], [25]] as an approach to rapidly explore a diverse set of amides in the hope of identifying a suitable compound which maintained potency and showed improved solubility and microsomal stability. The aim of the plate-based chemistry was to use liquid handling robotics to rapidly produce an array of compounds which could inform the SAR, without the need for the time-consuming steps of purification and isolation. Synthesis, analysis and assay plating can all be carried out from the microtiter plates used. Relative to batch synthesis tiny amounts of material are required, typically of the order of 0.1 mg of template per well. In this way 384 compounds can be made and screened with around 50 mg of material in hand. At the DDU we have invested time in standardising reliable chemistry methods which allows automated synthesis to be conducted with good synthetic reliability. Optimisation of reaction conditions is key when using this approach to ensure that there is a good yield of product. Careful use of controls is also required, all reagents, solvents and starting materials employed are screened in the relevant assays to confirm they are inactive and will therefore not interfere with the output.

**Fig. 3:**
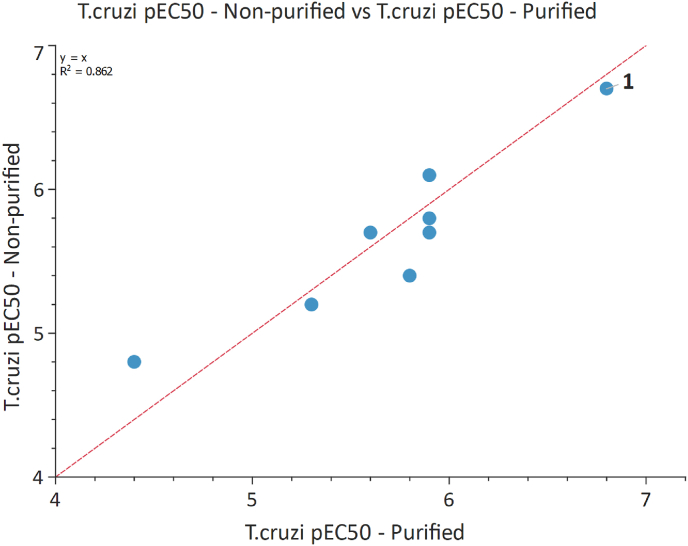
Comparison of *T. cruzi* pEC_50_ of purified and non-purified material for test set.

The technology was first validated with a series-specific test set. Eight compounds which had been previously synthesised, purified and tested were resynthesized in the plate format. All desired products were formed with purities ranging from 39 to 82% as reported from LCMS UV trace (254 nm). The reaction mixtures were tested directly in our intracellular *T. cruzi* assay, dose-response curves were generated, and the potencies of purified and non-purified material were compared. To our delight, the measured potencies correlated well, which validated this technique for use on our series and beyond this, across many other in-house projects (Fig. 3).

**Fig. 4:**
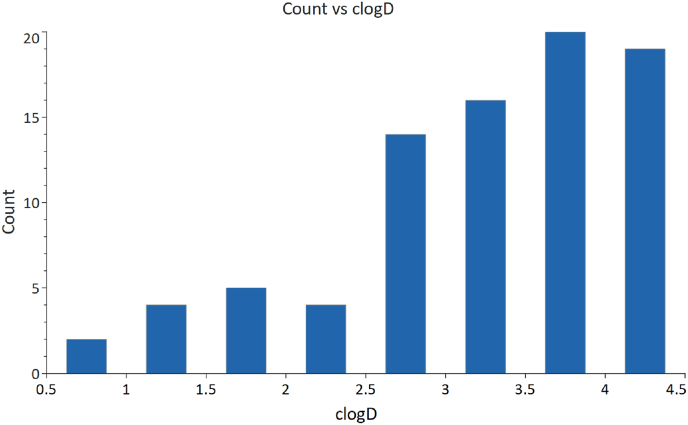
Plot showing number of compounds in each clogD bracket included in the plate-based array.

The amine scaffold was enumerated with our in-house acid compound collection and a clogD cut off of 4.5 was applied (clogD as calculated in Stardrop) to afford a set of 922 unique compounds. The set was assessed based on reactivity, toxicity and frequent hitters as well as assigning a drug likeness score (QED). Based on these outputs, several compounds were removed from the set to leave 659 potential compounds for synthesis. Fingerprints were generated for these molecules (Canvas [26,27]) and using these, a selection of 84 compounds was made to include a diverse range of aliphatic, aromatic, basic and neutral groups. We also aimed to include molecules with a range of clogD values (Fig. 4).

The array was run in a 96-well plate format and 84 reactions were performed. Of these 84 reactions, 63 led to formation of the desired product with conversions of 20–80% as estimated by LCMS UV trace (254 nm). These compounds were then tested directly at three concentrations in our intracellular *T. cruzi* assay (0.5, 5 and 50 μM based on complete conversion to desired product). One hit was identified as showing >50% effect with no cytotoxicity when tested at 0.5 μM and four further hits were identified when tested at 5 μM. These structurally diverse compounds were selected for resynthesis, purification and further profiling (Table 4). Of these hits, indole compound **23** was found to be the most potent but only showed a modest improvement in clearance and was considerably less soluble. Unfortunately, the other hits identified **24**–**27** were found to be at least one log unit less potent than **4**. Alkyne compound **26** showed considerably reduced ChromlogD_pH7.4_ when compared to **4** (c.f 4.4 vs 5.6) and exhibited superior solubility. However, this compound was inactive when the purified batch was tested.

**Table 4:**
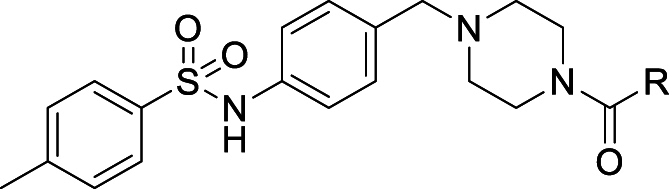
Hits identified from plate-based array.

R	Number	*T. cruzi*^a^ (pEC_50_)	Mouse CL_*i*_^b^ (mL/min/g)	Solubility^c^ (μM)	ChromlogD_pH7.4_^d^	clogD
	**23**	6.7	40	<1	5.6	2.4
	**24**	5.4	>50	35	5.8	2.8
	**25**	5.2	>50	22	5.7	2.9
	**26**	<4.3	>50	367	4.4	1.4
	**27**	5.4	>50	29	5.4	2.7

a *T. cruzi* pEC_50_: potency against intracellular *T. cruzi* amastigotes, data from at least three independent replicates, standard deviations ≤0.2.

b Cl_i_ is mouse liver microsomal intrinsic clearance.

c Kinetic solubility measured by CAD (Charged Aerosol Detector).

d ChromLogD_pH7.4_ = CHI_pH7.4_ × 0.0857–2 where CHI is chromatographic hydrophobicity index [20].

A further plate-based array of 52 compounds was made focused around compound **23**, but no improvement in activity or DMPK properties was achieved.

The plate-based array approach undoubtedly facilitated a rapid SAR expansion allowing us to investigate structural diversity without the need for a purification step. Unfortunately, in the case of this series no compounds with the desired balance of potency, solubility and metabolic stability were identified. This could indicate that lipophilic amides and sulphonamides are required for interaction with the molecular target. Converse to this, the relatively high lipophilicity appeared to be driving poor solubility and microsomal instability [28,29]. In the absence of knowledge of the molecular target and any structural information, we could not identify any further opportunities to optimise these terminal moieties.

**Fig. 5:**
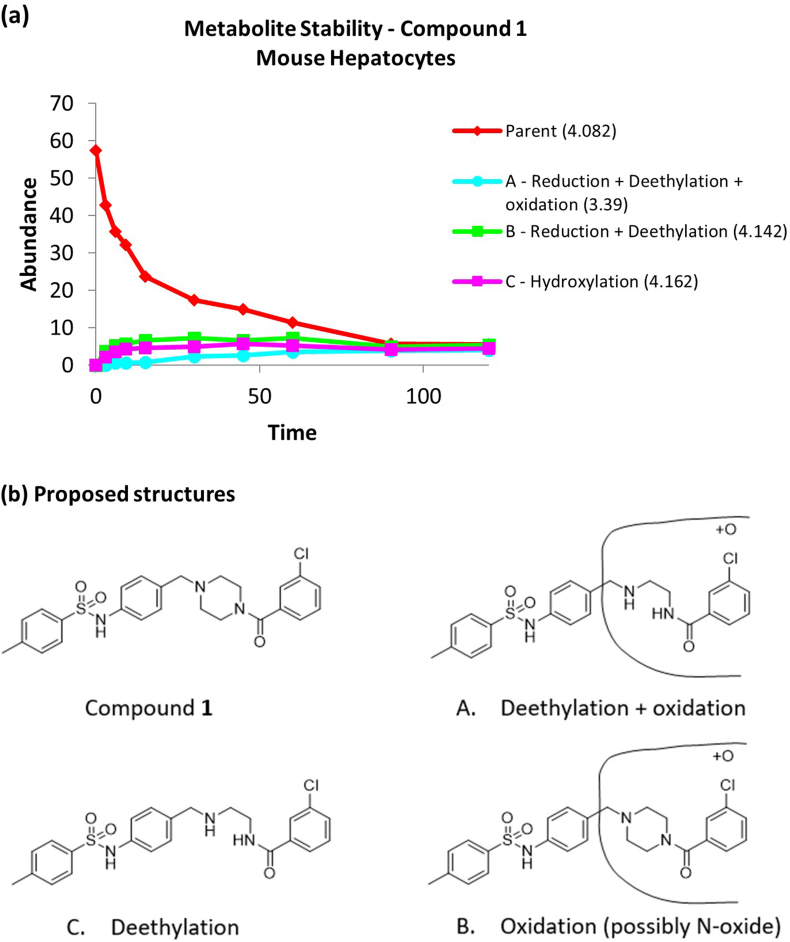
(a) the loss of parent and appearance of metabolites when compound **1** was incubated with mouse liver microsomes; (b) the proposed structures of the metabolites.

To investigate the high metabolic clearance of the hit further a metID study was performed (Fig. 5). Compounds were incubated with mouse liver microsomes and metabolites were identified using LCMS-MS. This work revealed that the embedded piperazine moiety is a metabolic hot spot with two of the identified metabolites indicating deethylation of this group (A and B). A third metabolite (C) suggested oxidation of the molecule, the point of which could not be specified but may be occurring on the piperazine ring with formation of the N-oxide.

With the metID study identifying the embedded piperazine as a metabolic liability we hypothesised that finding a suitable isostere could retain potency and reduce the clearance of the series. We designed and synthesised a set of 13 compounds including known piperazine isosteres from the literature [30,31]. Compounds with a range of clogD values were included with a focus on those in similar or lower clogD space compared to compound **4** (clogD 2.9, Table 5), as isosteres which increased logD were unlikely to improve metabolic stability or solubility. Dihydropyrrolopyrazole core **29** showed the most significant improvement in clearance compared to compound **4** (12 compared to >50 ml/min/g) with a reduction in ChromlogD_pH7.4_ despite a higher clogD. This compound was still not in desirable stability space and was over a log unit less potent than compound **4**. All other isosteric replacements synthesised **28**, **30**–**40** showed no substantial improvement in metabolic stability and significantly reduced potency against the *T. cruzi* parasites.

**Table 5:** Compounds containing isosteric replacements of the piperazine moiety.

Structure	Number	*T. cruzi*^a^ (pEC_50_)	Mouse CL_*i*_^b^ (mL/min/g)	Solubility^c^ (μM)	ChromlogD_pH7.4_^d^	clogD
	**4**	6.3	>50	43	5.6	2.9
	**28**	5.3	37	<1	5.9	4.1
	**29**	4.9	12	19	5.0	3.6
	**30**	4.3	>50	51	5.1	3.1
	**31**	5.0	>50	72	4.9	2.5
	**32**	4.8	48	75	4.9	2.5
	**33**	5.2	>50	27	5.7	2.8
	**34**	5.0	>50	28	5.7	2.8
	**35**	5.2	>50	25	5.8	3.4
	**36**	4.7	25	18	5.6	2.9
	**37**	5.5	37	42	5.3	3.4
	**38**	4.9	>50	70	4.8	2.4
	**39**	4.8	>50	47	5.1	2.9
	**40**	5.3	>50	35	5.5	2.9

a *T. cruzi* pEC_50_: potency against intracellular *T. cruzi* amastigotes, data from at least three independent replicates, standard deviations ≤0.2.

b Cl_i_ is mouse liver microsomal intrinsic clearance.

c Kinetic solubility measured by CAD (Charged Aerosol Detector).

d ChromLogD_pH7.4_ = CHI_pH7.4_ × 0.0857–2 where CHI is chromatographic hydrophobicity index [20].

Having thoroughly explored changes to the piperazine ring, we turned our attention to the central phenyl moiety with the intention of introducing polarity to reduce logD and improve metabolic stability (Table 6). Introducing oxadiazole **41** was successful in driving down ChromlogD_pH7.4_ and moving solubility and clearance into excellent space. However, disappointingly this compound was inactive. Likewise, introducing various 6-membered heterocycles such as pyrimidines **42**, **43** and pyrazines **45**, **46** showed superior solubility and microsomal stability but lacked any activity in the intracellular *T. cruzi* assay. Pyridine compound **44** retained some activity and showed improved solubility but the metabolic stability was still poor.

**Table 6:**
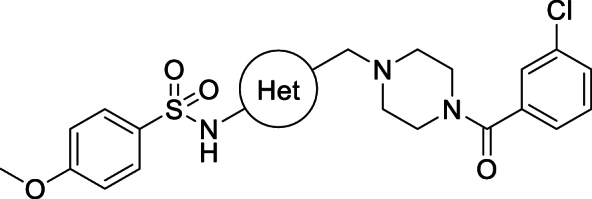
Compounds containing replacements of the central phenyl moiety.

Structure	Number	*T. cruzi*^a^ (pEC_50_)	Mouse CL_*i*_^b^ (mL/min/g)	Solubility^c^ (μM)	ChromlogD_pH7.4_^d^
	**4**	6.3	>50	43	5.6
	**41**	<4.3	0.6	≥404	1.9
	**42**	<4.3	1.0	≥345	3.1
	**43**	<4.3	1.5	≥474	2.9
	**44**	5.1	39	≥373	4.5
	**45**	<4.3	1.3	≥414	2.8
	**46**	<4.3	1.2	≥443	4.0

a *T. cruzi* pEC_50_: potency against intracellular *T. cruzi* amastigotes, data from at least three independent replicates, standard deviations ≤0.2.

b Cl_i_ is mouse liver microsomal intrinsic clearance.

c Kinetic solubility measured by CAD (Charged Aerosol Detector).

d ChromLogD_pH7.4_ = CHI_pH7.4_ × 0.0857–2 where CHI is chromatographic hydrophobicity index [20].

**Fig. 6:**
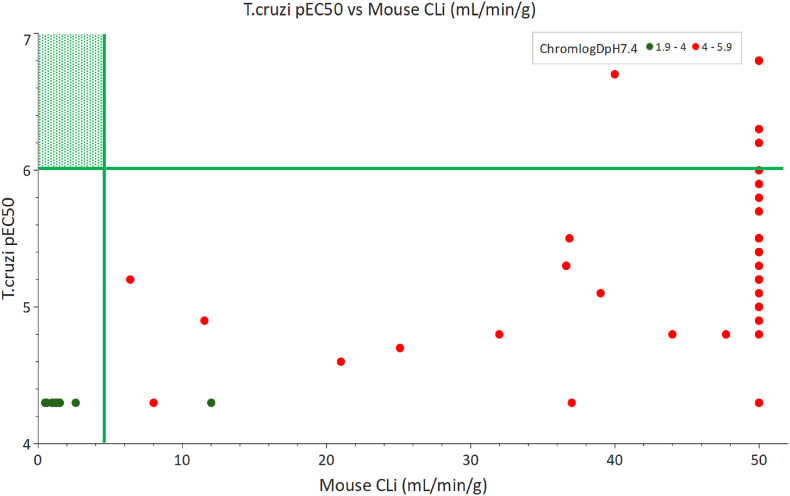
Plot of potency vs microsomal clearance. Green lines show desired property cut offs of pEC_50_ > 6 and clearance <5 mL/min/g. Desired property space is coloured in green. (For interpretation of the references to colour in this figure legend, the reader is referred to the Web version of this article.)

Having explored various strategies to identify a suitable compound which retained potency and improved metabolic stability and solubility we performed a thorough analysis of the series. On inspection of the available SAR it was apparent that within the series ChromlogD_pH7.4_ > 4 was required to achieve the desired potency level of pEC_50_ > 6. Conversely, ChromlogD_pH7.4_ < 4 was necessary to have any hope of achieving Cl < 5 mL/min/g (Fig. 6). Considering this information, the decision was taken to close the series.

### 2.1 Synthesis

**Scheme 1:**
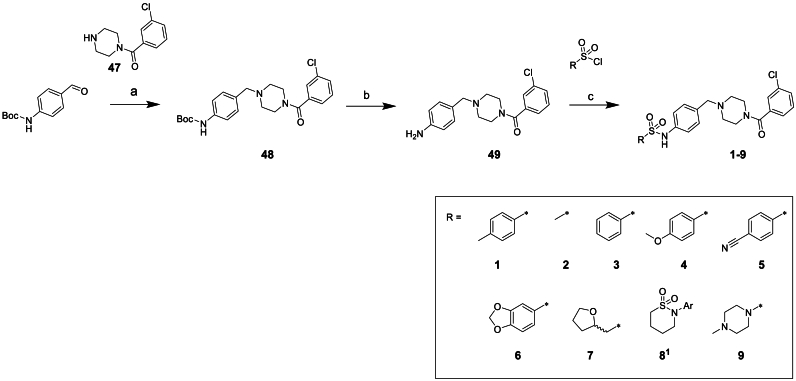
**Synthesis of varied sulphonamide compounds 1-9**^a^ ^a^Reagents and conditions: (a) NaBH(OAc)_3_, anh. MgSO_4_, DCM, rt, 5 h (b) 4 M HCl in dioxane, DCM, rt, 16 h (c) triethylamine, DCM, 0°C-rt, 16 h or pyridine, 0°C-rt, 16 h ^1^Nitrogen linked directly to aromatic ring of core structure.

As shown in Scheme 1 changes to the terminal sulfonamide could be easily accessed from intermediate aniline **49**. This was afforded in two steps from commercially available N-Boc-p-aminobenzaldehyde, performing a reductive amination with **47** followed by a Boc deprotection. With aniline **49** in hand, reaction with the relevant sulfonyl chloride yielded the desired sulfonamide products **1**–**9**.

**Scheme 2:**
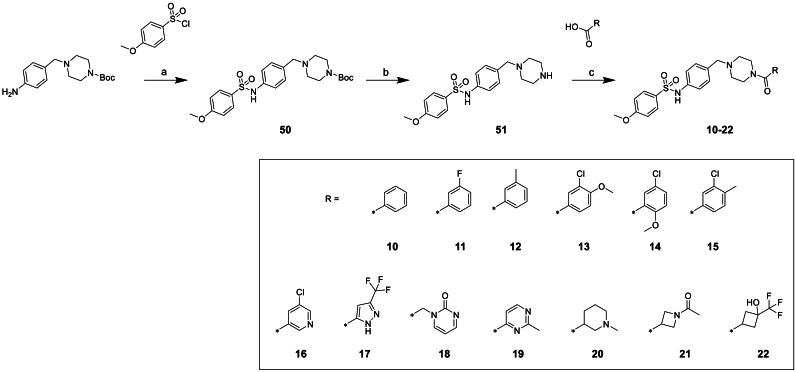
**Synthesis of varied amide compounds 10–22**^a^ ^a^Reagents and conditions: (a) DCM, rt, 16 h (b) TFA, DCM, rt, 20 h (c) T3P, triethylamine, DCM, rt, 5 h.

Variation of the terminal amide (Scheme 2) could be easily investigated from common piperazine intermediate **51**. This could be afforded from commercially available 4-(1-Boc-piperazin-4-yl-methyl)-aniline by first reacting with p-methoxy benzenesulphonyl chloride to install the sulphonamide **50** followed by Boc deprotection. With this advanced intermediate in hand an amide coupling reaction afforded products **10–22**.

**Scheme 3:**
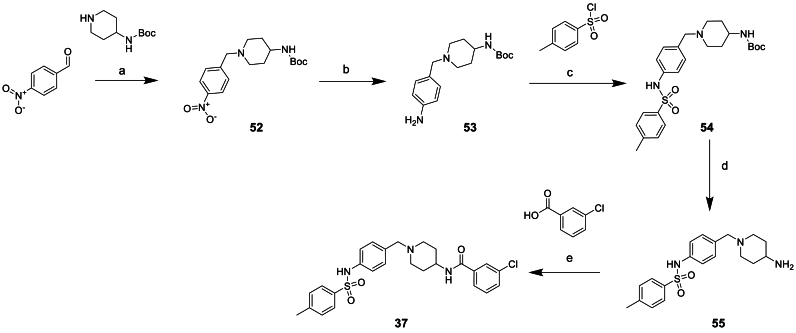
Synthesis of piperazine replacement compound 37^a^ ^a^Reagents and conditions: (a) NaBH(OAc)_3_, DCM, 0°C-rt, 22 h (b) Zn, NH_4_Cl, THF:water, 0°C-rt, 16 h (c) triethylamine, DCM, 0°C-rt, 5 h (d) TFA, DCM, 0°C-rt, 4 h (e) HATU, DIPEA, DCM, 0°C-rt, 16 h.

**Scheme 4:**
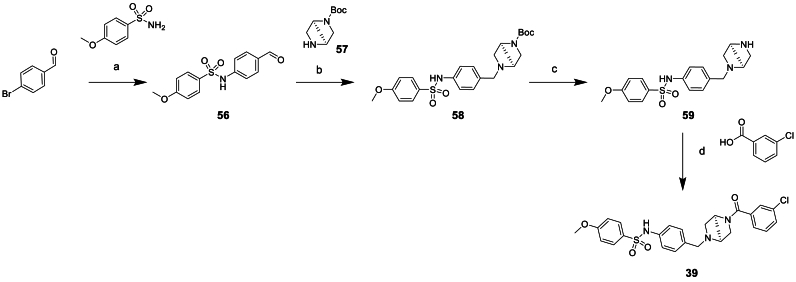
Synthesis of piperazine replacement compound 39^a^ ^a^Reagents and conditions: (a) Pd_2_(dba)_3_, XPhos, K_3_PO_4_, 1,4-dioxane, 100 °C, 16 h (b) NaBH(OAc)_3_, THF, rt, 3 h (c) 4 M HCl dioxane, MeOH, rt, 16 h (d) HOBt, EDC.HCl, triethylamine, DCM, rt, 18 h.

**Scheme 5:**
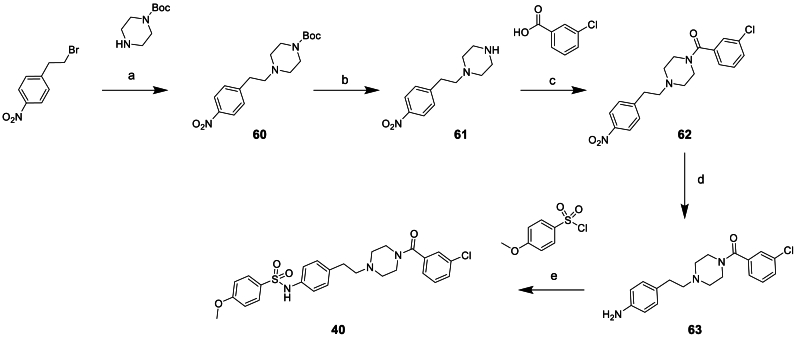
Synthesis of piperazine replacement compound 40^a^ ^a^Reagents and conditions: (a) K_2_CO_3_, MeCN, rt 16 h (b) 4 M HCl in dioxane, MeOH, rt, 24 h (c) HOBt, EDC.HCl, DIPEA, DCM, rt, 72 h (d) Zn, AcOH, DCM, rt, 2.5 h (e) DIPEA, DCM, 0°C-rt, 16 h.

All the compounds containing replacements of the central piperazine unit **28**–**40** were made via one of the routes exemplified in Scheme 3, Scheme 4, Scheme 5 or similar, based on commercially available starting materials.

The synthesis of compound **37** started from 4-nitrobenzaldehyde, as outlined in Scheme 3. The reductive amination to give **52** was followed by nitro reduction to afford **53** which underwent reaction with 4-methylbenzenesulfonyl chloride to give **54**. Subsequent Boc deprotection afforded amine **55** which could then be coupled with 3-chlorobenzoic acid yielding compound **37**.

The synthesis of bridged piperazine compound **39** started with a palladium facillitated sulfonamidation with 4-bromobenzaldehyde to afford aldehyde intermediate **56**, Scheme 4. Subsequent reductive amination with bridged piperazine monomer **57** followed by Boc deprotection afforded amine intermediate **59**. This could then undergo amide coupling with 3-chlorobenzoic acid to afford compound **39**.

The synthesis of compound **40** started from 1-(2-bromoethyl)-4-nitrobenzene, as shown in Scheme 5. Substitution of the bromo with *tert*-butyl piperazine-1-carboxylate to give **60** was followed by Boc deprotection and subsequent amide coupling with 3-chlorobenzoic acid to give **62**. Nitro reduction using zinc and acetic acid afforded **63** which could then be reacted with 4-methoxybenzenesulfonyl chloride to give **40**.

**Scheme 6:**
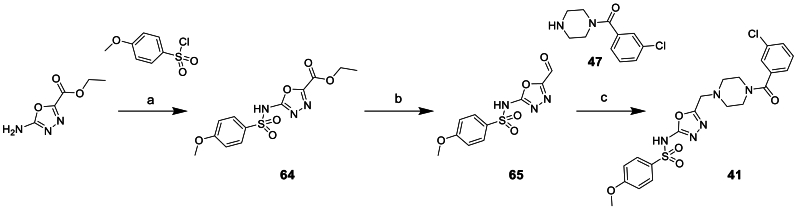
Synthesis of phenyl replacement compound 41^a^ ^a^Reagents and conditions: (a) NaH, DMF, 0°C-rt, 2 h (b) LiAlH_4_, THF, −78 °C, 1 h (c) NaBH(OAc)_3_, anh. MgSO_4_, DCM, 0°C-rt, 12 h.

**Scheme 7:**
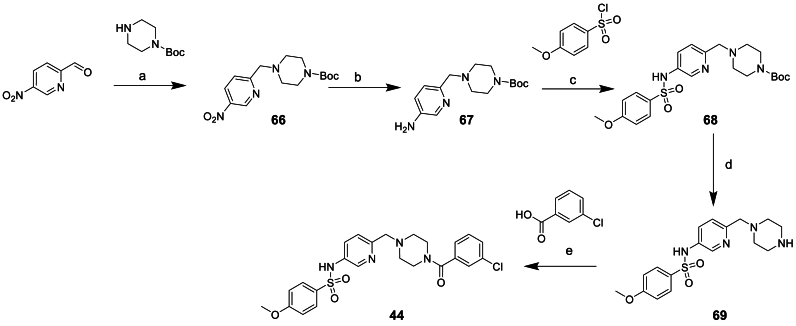
Synthesis of phenyl replacement compound 44^a^ ^a^Reagents and conditions: (a) NaBH(OAc)_3_, DCM, rt, 16 h (b) Zn, NH_4_Cl, THF:H20, rt, 24 h (c) pyridine, 0°C-rt, 1 h (d) TFA, DCM, 0°C-rt, 5 h (e) T3P, DIPEA, DCM, rt, 16 h.

**Scheme 8:**
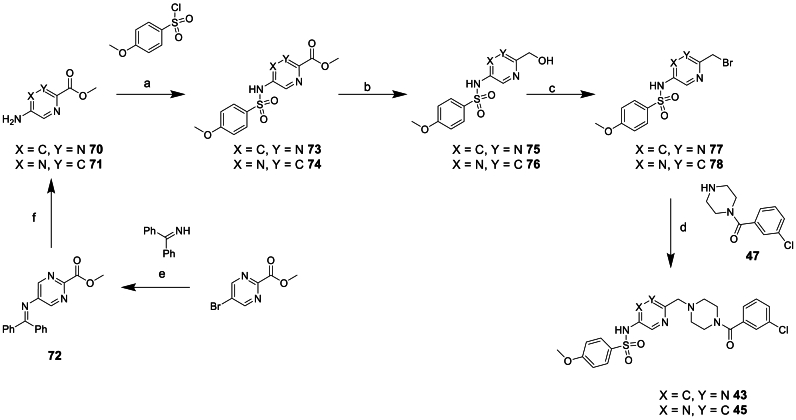
Synthesis of phenyl replacement compounds 43 and 45^a^ ^a^Reagents and conditions: (a) Pyridine, 60 °C, 16 h (b) LiBH_4_, EtOH:Et_2_O:THF, 0°C-rt, 20 h (c) PBr_3_, DCM, 0°C-rt, 1 h (d) K_2_CO_3_, DMF, rt, 16 h (e) [Pd(OAc)_2_]_3_, BINAP, Cs_2_CO_3_, toluene, 100 °C, 5 h (f) HCl, MeOH, rt, 16 h.

Compounds **41**–**46**, synthesised to investigate changes to the central phenyl moiety, were afforded via routes exemplified in Scheme 6, Scheme 7, Scheme 8 or similar, depending on commercial availability of starting materials.

Compound **41** was prepared as outlined in Scheme 6 starting from ethyl 5-amino-1,3,4-oxadiazole-2-carboxylate. Reaction with 4-methoxybenzenesulfonyl chloride to give **64** was followed by reduction of the ethyl ester moiety using LiAlH_4_. This afforded a mixture of aldehyde **65** and the expected alcohol product. This mixture was used directly in a reductive amination reaction with **47** and yielded **41**.

The synthesis of compound **44** started from 5-nitropicolinaldehyde as shown in Scheme 7. Reductive amination with *tert*-butyl piperazine-1-carboxylate to give **66** was followed by zinc mediated nitro reduction to afford **67**. Subsequent sulfonamide formation with 4-methoxybenzenesulfonyl chloride and Boc deprotection afforded amine **69** which was then coupled to 3-chlorobenzoic acid yielding **44**.

Heterocyclic replacements for the phenyl moiety, **43** and **45** were prepared as shown in Scheme 8. Starting material **71** was commercially available but **70** had to be synthesised. Starting from methyl 5-bromopyrimidine-2-carboxylate Buchwald coupling with benzophenone imine afforded **72** which could then be hydrolysed under acidic conditions to afford the desired starting material **70**. Sulfonamide formation to afford **73**/**74** was followed by reduction of the methyl ester to the alcohol using LiBH_4_ to give **75**/**76**. The alcohol was converted to the benzyl halide using PBr_3_ to give **77**/**78**, subsequent substitution of the halide with **47** afforded compounds **43** and **45**.

## 3 Conclusions

Through a phenotypic screen, we have identified a compound series which shows good activity against *T. cruzi.* Despite numerous strategies to mitigate the metabolic instability of this series, no compound was identified which maintained the desired potency whilst reducing clearance to suitable levels for *in vivo* studies. We have demonstrated that the strategies adopted including lowering logD, scoping out diversity and replacing particular metabollically labile groups can indeed improve intrinsic clearance. Also, the use of high-throughput plate-based chemistry proved an effective way to rapidly explore SAR. Unfortunately, in the case of this series, the changes which improved metabolic stability were associated with loss of potency against the target parasite. This is demonstrated in Fig. 6 which clearly illustrates there is no overlap in the ChromLogD_pH7.4_ space required to achieve potency and the ChromLogD_pH7.4_ space required to achieve suitable clearance.

Both the *in vitro* and *in vivo* profiles of compound **1** show that the mechanism of action is potentially suitable for developing a novel treatment for Chagas disease, although it proved challenging to progress this series. Establishing the molecular target and understanding how our compound interacts with this may provide a clearer route to optimising this compound series.

## 4 Notes

The authors declare the following competing financial interest(s): Several authors have shares in GlaxoSmithKline.

All regulated procedures on living animals in Dundee were carried out under the authority of a project license issued by the Home Office under the Animals (Scientific Procedures) Act 1986, as amended in 2012 (and in compliance with EU Directive EU/2010/63). License applications have been approved by the University's Ethical Review Committee (ERC) before submission to the Home Office. The ERC has a general remit to develop and oversee policy on all aspects of the use of animals on University premises and is a subcommittee of the University Court, its highest governing body. All animal studies carried out by GSK were reviewed by GlaxoSmithKline's (GSK) internal ethical review committee and performed in accordance with Animals (Scientific Procedures) Act 1986 and the GSK Policy on the Care, Welfare, and Treatment of Laboratory Animals (UK 1986).

## Declaration of competing interest

The authors declare that they have no known competing financial interests or personal relationships that could have appeared to influence the work reported in this paper.

## References

[1] Echeverría L.E., Marcus R., Novick G. (2020). WHF IASC roadmap on chagas disease. Glob. Heart..

[2] https://www.who.int/news-room/fact-sheets/detail/chagas-disease-(american-trypanosomiasis).

[3] GBD 2019 Diseases and Injuries Collaborators (2020). Global burden of 369 diseases and injuries in 204 countries and territories, 1990–2019: a systematic analysis for the Global Burden of Disease Study 2019. Lancet.

[4] Kratz J.M. (2019). Drug discovery for chagas disease: a viewpoint. Acta Trop..

[5] Requena-Mendez A., Aldasoro E., de Lazzari E., Sicuri E., Brown M., Moore D.A., Gascon J., Muñoz J. (2015). Prevalence of Chagas disease in Latin-American migrants living in Europe: a systematic review and meta-analysis. PLoS Neglected Trop. Dis..

[6] Bern C., Messenger L.A., Whitman J.D., Maguire J.H. (2019). Chagas disease in the United States: a public health approach. Clin. Microbiol. Rev..

[7] Cevallos A.M., Chagas’ Disease Hernández R. (2014). Pregnancy and Congenital Transmission *BioMed Res. Int.*.

[8] Chatelain E. (2017). Chagas disease research and development: is there light at the end of the tunnel?. Comput. Struct. Biotechnol. J..

[9] Pérez-Molina J.A., Molina I. (2018). Chagas disease *Lancet*.

[10] Chatelain E. (2015). Chagas disease drug discovery: towards a New Era. J. Biomol. Screen.

[11] Wilkinson S.R., Kelly J.M. (2009). Trypanocidal drugs: mechanisms, resistance and new targets. Expet Rev. Mol. Med..

[12] Gaspar L., Moraes C.B., Freitas-Junior L.H., Ferrari S., Costantino L., Costi M.P., Coron R.P., Smith T.K., Siqueira-Neto J.L., McKerrow J.H. (2015). Current and future chemotherapy for Chagas disease. Curr. Med. Chem..

[13] Molina I., Gómez i Prat J., Salvador F., Treviño B., Sulleiro E., Serre N., Pou D., Roure S., Cabezos J., Valerio L., Blanco-Grau A., Sánchez-Montalvá A. (2014). Randomized trial of posaconazole and benznidazole for chronic chagas' disease. N. Engl. J. Med..

[14] Torrico F., Gascon J., Ortiz L., Alonso-Vega C., Pinazo M.J., Schijman A., Almeida I.C., Alves F., Strub-Wourgaft N., Ribeiro I. (2018). Treatment of adult chronic indeterminate Chagas disease with benznidazole and three E1224 dosing regimens: a proof-of-concept, randomised, placebo-controlled trial. Lancet Infect. Dis..

[15] Riley J., Brand S., Voice M., Caballero I., Calvo D., Read K.D. (2015). Development of a fluorescence-based trypanosoma cruzi CYP51 inhibition assay for effective compound triaging in drug discovery programmes for chagas disease. PLoS Neglected Trop. Dis..

[16] Svensen N., Wyllie S., Gray D.W., De Rycker M. (2021). Live-imaging rate-of-kill compound profiling for Chagas disease drug discovery with a new automated high-content assay. PLoS Neglected Trop. Dis..

[17] MacLean L.M., Thomas J., Lewis M.D., Cotillo I., Gray D.W., De Rycker M. (2018). Development of Trypanosoma cruzi in vitro assays to identify compounds suitable for progression in Chagas' disease drug discovery. PLoS Neglected Trop. Dis..

[18] De Rycker M., Thomas J., Riley J., Brough S.J., Miles T.J. (2016). Identification of trypanocidal activity for known clinical compounds using a new *Trypanosoma cruzi* hit-discovery screening cascade. PLoS Neglected Trop. Dis..

[19] Pena I., Manzano M.P., Cantizani J., Kessler A., Alonso-Padilla J., Bardera A.I., Alvarez E., Colmenarejo G., Cotillo I., Roquero I., de Dios-Anton F., Barroso V., Rodriguez A., Gray D.W., Navarro M., Kumar V., Sherstnev A., Drewry D.H., Brown J.R., Fiandor J.M., Martin J.J. (2015). New compound sets identified from high throughput phenotypic screening against three kinetoplastid parasites: an open resource. Sci. Rep..

[20] Lewis M.D., Fortes Francisco A., Taylor M.C., Kelly J.M. (2015). A new experimental model for assessing drug efficacy against *Trypanosoma cruzi* infection based on highly sensitive in vivo imaging. J. Biomol. Screen.

[21] Stepan A.F., Mascitti V., Beaumontc K., Kalgutkar A.S. (2013). Metabolism-guided drug design Med. Chem. Commun. (J. Chem. Soc. Sect. D).

[22] Young R.J., Green D.V.S., Luscombe C.N., Hill A.P. (2011). Getting physical in drug discovery II: the impact of chromatographic hydrophobicity measurements and aromaticity. Drug Discov.

[23] Cernak T., Gesmundo N.G., Dykstra K., Yu Y., Wu Z., Shi Z., Vachal P., Sperbeck D., He S., Murphy B.A., Sonatore L., Williams S., Madeira M., Verras A., Reiter M., Heechoon Lee C., Cuff J., Sherer E.C., Kuethe J., Goble S., Perrotto N., Pinto S., Shen D., Nargund R., Balkovec J., DeVita R.J., Dreher S.D. (2017). Microscale high-throughput experimentation as an enabling technology in drug discovery: application in the discovery of (Piperidinyl)pyridinyl-1H-benzimidazole diacylglycerol acyltransferase 1 inhibitors. J. Med. Chem..

[24] Gesmundo N.J., Sauvagnat B., Curran P.J., Richards M.P., Andrews C.L., Dandliker P.J., Cernak T. (2018). Nanoscale synthesis and affinity ranking *Nature*.

[25] Santanilla A.B., Regalado E.L., Pereira T., Shevlin M., Bateman K., Campeau L.C., Schneeweis J., Berritt S., Shi Z.C., Nantermet P., Liu Y., Helmy R., Welch C.J., Vachal P., Davies I.W., Cernak T., Dreher S.D. (2015). Nanomole-scale high-throughput chemistry for the synthesis of complex molecules. Science.

[26] https://www.schrodinger.com/products/canvas.

[27] Duan J., Dixon S.L., Lowrie J.F., Sherman W. (2010). Analysis and comparison of 2D fingerprints: insights into database screening performance using eight fingerprint methods. J. Mol. Graph. Model..

[28] Leeson P.D. (2016). Molecular inflation, attrition and the rule of five. Adv. Drug Deliv. Rev..

[29] Young R.J., Leeson P.D. (2018). Mapping the efficiency and physicochemical trajectories of successful optimizations. J. Med. Chem..

[30] Mowbray C.E., Bell A.S., Clarke N.P., Collins M., Jones R.M., Lane C.A.L., Liu W.L., Newman S.D., Paradowski M., Schenck E.J., Selby M.D., Swain N.A., Williams D.H. (2011). Challenges of drug discovery in novel target space. The discovery and evaluation of PF-3893787: a novel histamine H4 receptor antagonist. Bioorg. Med. Chem. Lett.

[31] Luo G., Mattson G.K., Bruce M.A., Wong H., Murphy B.J., Longhi D., Antal-Zimanyic I., Poindexter G.S. (2004). Isosteric N-arylpiperazine replacements in a series of dihydropyridine NPY1 receptor antagonists. Bioorg. Med. Chem. Lett.

