# Supplementary material for: Identification and development of a series of disubstituted piperazines for the treatment of Chagas disease
Source: Eur J Med Chem. 2022 Aug 5;238:114421. doi: 10.1016/j.ejmech.2022.114421 (PMC11458808; doi:10.1016/j.ejmech.2022.114421)
Supplement: Multimedia component 1 [file mmc1.docx]

**Identification and development of a series of disubstituted piperazines for the treatment of Chagas disease**

Kate McGonagle, Gary J. Tarver, Juan Cantizani, Ignacio Cotillo, Peter G. Dodd, Liam Ferguson, Ian H. Gilbert, Maria Marco, Tim Miles, Claire Naylor, Maria Osuna-Cabello, Christy Paterson, Kevin D. Read, Erika G. Pinto, Jennifer Riley, Paul Scullion, Yoko Shishikura, Frederick Simeons, Laste Stojanovski, Nina Svensen, John Thomas, Paul G. Wyatt, Pilar Manzano*, Manu De Rycker* and Michael G. Thomas*

**Contents**

1. **In vitro assays…………………………………………………………………………………………………………………..3**
   1. **Intracellular *T. cruzi* assay…………………………………………………………………………………………..3**
   2. **Washout assay……………………………………………………………………………………………………………3**
   3. **Rate of kill studies………………………………………………………………………………………………………3**
   4. **CYP51 assay………………………………………………………………………………………………………………..3**
2. **DMPK assays…………………………………………………………………………………………………………………….3**
   1. **Aqueous solubility………………………………………………………………………………………………………3**

**2.2 Intrinsic clearance……………………………………………………………………………………………………….3**

**2.3 Metabolite identification studies…………………………………………………………….………………….3**

1. **In vivo studies…………………………………………………………………………………………………………………..3**
   1. **In vivo mouse efficacy model……………………………………………………………………………………..3**
2. **Supplementary tables and figures…………………………………………………………………………………….4**
3. **Chemical synthesis…………………………………………………………………………………………………………...6**
   1. **General experimental information……………………………………………………………………………..6**
   2. **Synthesis – General……………………………………………………………………………………………………7**
   3. **Synthesis – Plate-based…………………………………………………………………………………………….14**
   4. **Final compound experimental data…………………………………………………………………………..14**
4. **^1^H, ^13^C NMR spectra and HRMS trace of final compounds………………………………………………..21**
5. **References………………………………………………………………………………………………………………………83**

**1. *In vitro* assays**

**1.1 Intracellular *T. cruzi* assay:** Potency against intracellular *T. cruzi* X10/7 A1 amastigotes was determined as described previously [1] with as sole modification that the compound treatment duration was increased from 72h to 96h.

**1.2 Washout assay:** Vero cells were infected overnight at MOI 5 with *T. cruzi* X10/7 A1 trypomastigotes in MEM media (Gibco) containing 10% FCS (Hyclone) followed by washing of the monolayer to remove any extracellular trypomastigotes. 24 h later, infected cells were trypsinised and plated in T25 flasks in presence of compounds at the indicated concentrations and incubated for 8 days in MEM media (Gibco) containing 1% FCS. Four days into the treatment the media was replaced with fresh compound-containing media. After the treatment period, monolayers were washed extensively. The cultures were then maintained, replacing media twice weekly, until trypomastigotes could be observed by light microscopy. Observations were carried out twice weekly for up to 60 days post washout.

**1.3 Rate-of-kill studies:** Experiments with intracellular *T. cruzi* X10/7-E2Crimson were carried out as previously described in [2].

**1.4 CYP51 assay**: Potency against *T. cruzi* CYP51 was determined as previously described in [3].

**2. DMPK assays**

**2.1 Aqueous solubility:** This assay was conducted as previously described in [4].

**2.2 Intrinsic clearance:** This assay was conducted as previously described in [5].

**2.3 Metabolite identification studies:** Cryopreserved vials of mouse cryopreserved hepatocytes, supplied by Life Technologies, were thawed according to manufacturer’s instructions and cells resuspended in Williams Medium E (WME) containing cell maintenance supplement pack (CM4000, Life Technologies).  Hepatocytes were incubated in suspension (0.5 million cells/mL) in 48 well non-collagen coated cell culture plates for 10 minutes at 37^o^C, 5% CO_2_.  Upon addition of an equal volume of supplemented WME containing 10 mM test compound, an aliquot of incubation solution was removed to acetonitrile containing internal standard (final concentration 5 mM test compound and a cell density of 0.25 million cells/mL).  Similarly, aliquots were removed at 3, 6, 9, 15, 30, 45, 60, 90 and 120 minutes. 100 mL of 80:20 water:acetonitrile was added to all samples and the analysis plate was centrifuged for 10 min at room temperature prior to injection and analysis of samples by UPLC-MS/MS. Samples were analysed using a Waters Xevo G2 QToF equipped with an Acquity HPLC system. The chromatography method used a simple water/acetonitrile gradient elution (with 0.01% formic acid modifier) on a Waters Acquity UPLC BEH C_18_ Column, 1.7 µm, 2.1 mm x 50 mm. All ions between 50 and 1000 amu were collected (with MS^e^ fragmentation) and mass spectrometry data analysed using Waters Metabolynx software. Subsequently identified metabolites were subjected to ms/ms fragmentation and analysis to try to elucidate the position of the metabolic transformation.

**3. *In vivo* studies**

**3.1 *In vivo* mouse efficacy model:** This study was performed as previously described in [6].

**4. Supplementary tables and figures**

**Table S1:** Washout Experiment

| **Experiment** | **Compound** | **Conc. (μM)** | **Fold EC_50_** | **Relapse Day ^a^** |
| --- | --- | --- | --- | --- |
| a | **1** | 5 | 32 | 11 |
|  | posaconazole | 0.2 | 50 | 7 |
|  | benznidazole (replicate 1) | 36 | 12.5 | 18 |
|  | benznidazole (replicate 2) | 36 | 12.5 | 14 |
| b | **1** | 5.5 | 35 | 14 |
|  | posaconazole | 0.2 | 50 | 11 |
|  | benznidazole | 36 | 12.5 | 14 |
| c | **1** | 3.9 | 25 | 11 |
|  | posaconazole | 0.1 | 25 | 7 |
|  | benznidazole | 36 | 12.5 | 18 |
|  | benznidazole | 144 | 50 | >60 |

Results for three independent washout experiments (experiment c is reported in the main manuscript). *T. cruzi* infected Vero cells were treated for 8 days at the indicated concentrations (compounds replenished after 4 days), followed by extensive washing to remove compounds and incubation until parasites were observed microscopically. ^a^ day after washout of compounds on which egressed parasites were detected first.


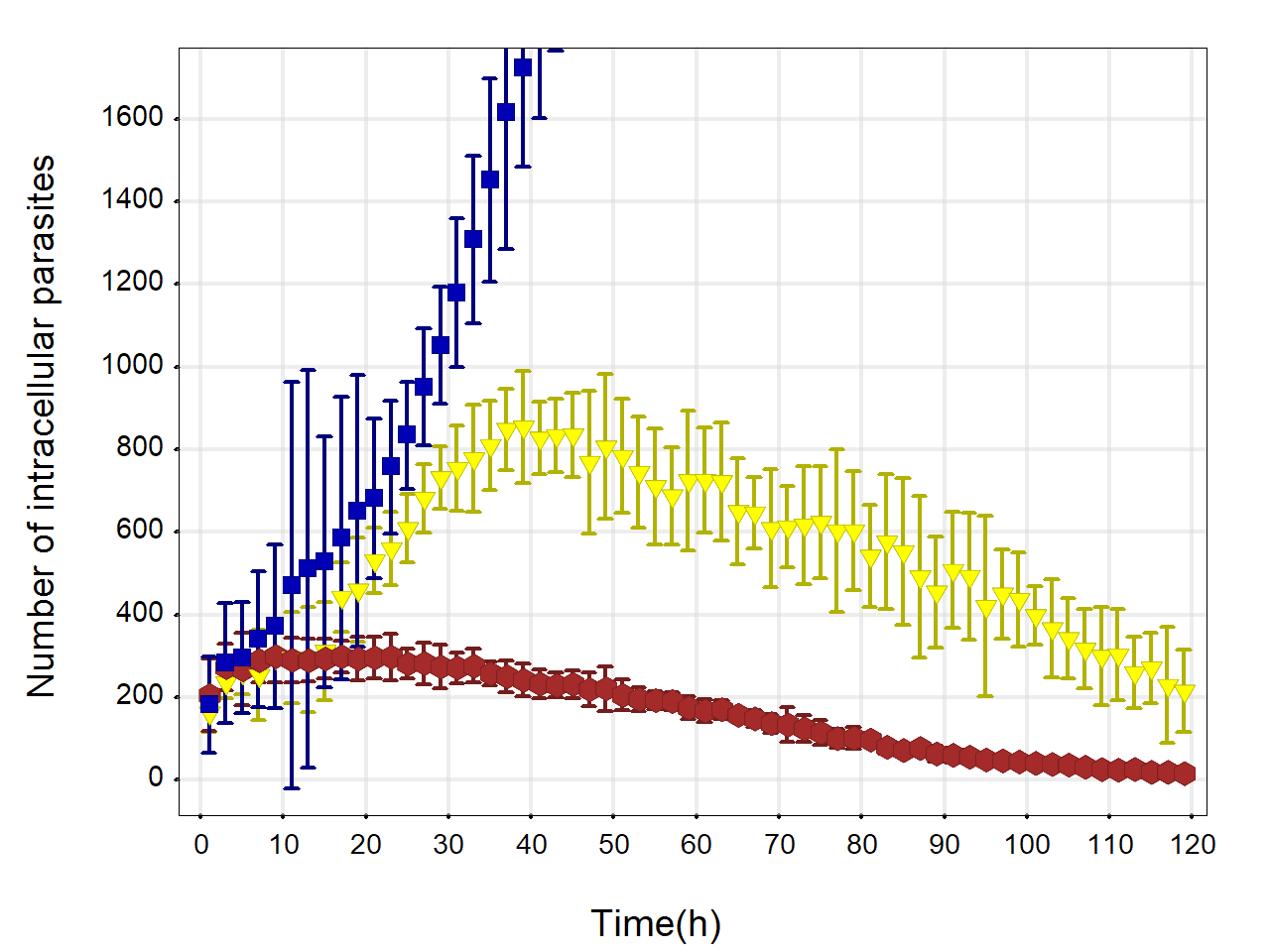


**Figure S1** Rate of kill experiment Compound **1** (red), Posaconazole (yellow), DMSO control (blue)


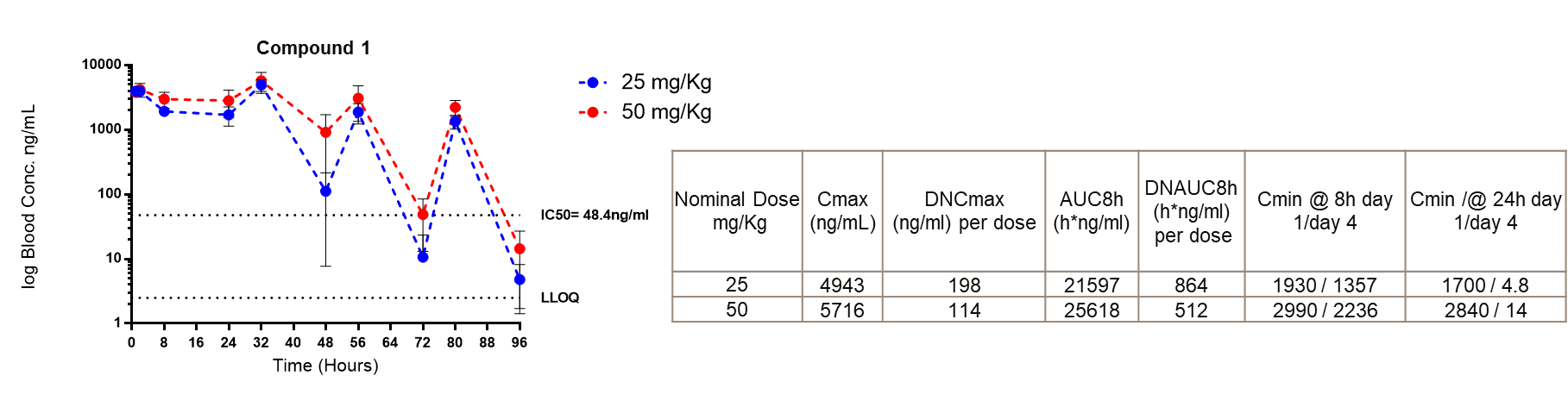


**Figure S2** Exposure of compound **1** in a 4-day PK study

**5. Chemical synthesis**

**5.1 General experimental information**

Chemicals and solvents were purchased from the Aldrich Chemical Company, Fluka, ABCR, VWR, Acros, Fluorochem and Alfa Aesar and were used as received. Air- and moisture-sensitive reactions were carried out under an inert atmosphere of nitrogen in oven-dried glassware. Analytical thin-layer chromatography (TLC) was performed on pre-coated TLC plates (layer 0.20 mm silica gel 60 with fluorescent indicator UV254, from Merck). Developed plates were air-dried and analyzed under a UV lamp (UV254/365 nm). Flash column chromatography was performed using pre-packed silica gel cartridges (230-400 mesh, 40–63 µm, from SiliCycle) using a Teledyne ISCO Combiflash Companion, or Combiflash Retrieve. ^1^H NMR and ^13^C NMR spectra were recorded on a Bruker Avance DPX 500 spectrometer (^1^H at 500.1 MHz, ^13^C at 125.8 MHz). Chemical shifts (d) are expressed in ppm recorded using the residual solvent as the internal reference in all cases. Signal splitting patterns are described as singlet (s), doublet (d), triplet (t), quartet (q), multiplet (m), broad (br), or a combination thereof. Coupling constants (J) are quoted to the nearest 0.1 Hz. Low resolution electrospray (ES) mass spectra were recorded on an Advion Compact mass spectrometer (CMS: model ExpressIon CMS) connected to Dionex Ultimate 3000 UPLC system with diode array detector. HPLC chromatographic separations were conducted using a Waters XBridge C_18_ column (2.1 x 50mm, 3.5 μm particle size) or Waters XSelect column (2.1 x 30mm, 2.5 μm particle size). The compounds were eluted with a gradient of 5-95% acetonitrile/water +0.1% ammonia or +0.1% formic acid. Alternatively, low resolution electrospray (ES) mass spectra were recorded on an Acquity UPLC (MS: Waters SQD; ELSD: Waters 2424; Waters PDA; Waters Binary solvent manager; Waters sample manager). HPLC chromatographic separations were conducted using a Waters Acquity BEH C_18_ column (3 x 50mm, 1.7 μm particle size). The compounds were eluted with a gradient of 5-95% acetonitrile/water +0.1% formic acid. Plate based chemistry was analysed on a Shimadzu Nexera X2 UHPLC. HPLC chromatographic separations were conducted using a Thermo Fisher Scientific Hypersil Gold column (2.1 x 50 mm, 1.9 µm particle size). The compounds were eluted with a gradient of 2-98% acetonitrile/water +0.05% formic acid. High resolution electrospray measurements were performed on a Waters Xevo QToF mass spectrometer coupled with a Waters Acquity UPLC system with diode array detector.  Separation of components was achieved using a Waters Acquity BEH C_18_ column (2.1 x 50mm, 1.7 μm particle size) eluting with 2-95% acetonitrile/water (both containing 0.01% formic acid), found mass is within 5 ppm unless otherwise stated. All intermediates had a measured purity ≥ 90% and all assay compounds had a measured purity of ≥ 95% as determined using one of the above analytical LC-MS system (TIC and UV) (excluding compounds synthesised via the plate-based method).

- 1. **Synthesis – General**

tert-butyl (4-((4-(3-chlorobenzoyl)piperazin-1-yl)methyl)phenyl)carbamate (**48**)

To a solution of (4-formyl-phenyl)-carbamic acid tert-butyl ester (125 mg, 0.56 mmol)  and  (3-chlorophenyl)-piperazin-1-yl-methanone (115 mg, 0.51 mmol)  in DCM (3 mL)  was added a spoonful of anh. MgSO_4_. The reaction was stirred for 3 h at rt before adding sodium triacetoxyborohydride (271 mg, 1.28 mmol). The reaction was stirred at rt for a further 2h. The reaction mixture was diluted with DCM (15 mL) and washed with NaHCO_3_ (satd. aq. 15 mL), the organic layer was collected and concentrated under reduced pressure. The material was solubilized in MeOH (5 mL) and subjected to SCX-2 cartridge (2 g) washing with MeOH before eluting compound with 7N NH_3_/MeOH. The NH_3_/MeOH fraction was concentrated under reduced pressure to afford title compound **48** (182 mg, 0.42 mmol, 82%). MS (ES+): *m/z* 431, 433 [M+H]^+^.

(4-(4-aminobenzyl)piperazin-1-yl)(3-chlorophenyl)methanone (**49**)

To a solution of tert-butyl N-[4-[[4-(3-chlorobenzoyl)piperazin-1-yl]methyl]phenyl]carbamate (**48**, 1.17 g, 2.72 mmol) in DCM (25 mL) was added 4 M HCl in dioxane (3.4 mL, 13.6 mmol).  The reaction was stirred at rt for 16 h and subsequently concentrated under reduced pressure. The material was solubilized in MeOH (20 mL) and purified by SCX-2 cartridge (20 g) washing with MeOH before eluting compound with 7 N NH_3_/MeOH. The NH_3_/MeOH fraction was concentrated under reduced pressure to afford the title compound **49** (726 mg, 2.2 mmol, 81%). MS (ES+): *m/z* 330, 332 [M+H]^+^.

N-(4-((4-(3-chlorobenzoyl)piperazin-1-yl)methyl)phenyl)-4-methoxybenzenesulfonamide (**4**)

To a solution of (4-(4-aminobenzyl)piperazin-1-yl)(3-chlorophenyl)methanone (**49**, 105 mg, 0.32 mmol) and triethylamine (0.13 ml, 0.96 mmol) in DCM (3.18 ml) at 0°C was added 4-methoxybenzenesulfonyl chloride (69 mg, 0.33 mmol). The reaction was stirred at 0°C for 4 h. Further 4-methoxybenzenesulfonyl chloride (69 mg, 0.33 mmol) was added and the reaction was stirred at 0°C to 10 °C for 12 h. The reaction was quenched with MeOH (2 mL) and concentrated under reduced pressure. The material was purified by column chromatography (12 g silica, 0-100% EtOAc in cyclohexane) then prep-HPLC (30-100% MeCN in water (+0.1% NH_3_)) to afford the title compound **4** as a white solid (60 mg, 0.12 mmol, 38%).; ^1^H NMR (500 MHz, DMSO-d_6_) δ 10.07 (br s, 1H), 7.69 – 7.66 (m, 2H), 7.52 – 7.50 (m, 1H), 7.48 – 7.45 (m, 1H), 7.42 – 7.41 (m, 1H), 7.33 – 7.32 (m, 1H), 7.16 – 7.14 (m, 2H), 7.06 – 7.02 (m, 4H), 3.79 (s, 3H), 3.59 – 3.57 (m, 2H), 3.38 (s, 2H), 3.29 – 3.26 (m, 2H), 2.35 – 2.28 (m, 4H).; ^13^C NMR (126 MHz, DMSO-d_6_) δ 167.8, 162.8, 138.5, 137.3, 133.8, 133.7, 131.8, 130.9, 130.1, 129.9, 129.3, 127.1, 125.9, 120.4, 114.8, 61.6, 56.1, 53.1, 52.6, 47.5, 42.1. Note, piperazine carbon peaks are weak and broad.; HRMS (ES^+^): m/z [M + H]^+^ calcd for C_25_H_27_N_3_O_4_SCl, 500.1411; found, 500.1432.

tert-butyl 4-(4-((4-methoxyphenyl)sulfonamido)benzyl)piperazine-1-carboxylate (**50**)

To a solution of tert-butyl 4-[(4-aminophenyl)methyl]piperazine-1-carboxylate (5.0 g, 17.2 mmol) in DCM (110 mL) at 0°C was added a solution of  4-methoxy benzenesulphonyl (3.9 g, 18.9 mmol) in DCM (20 mL). The reaction was allowed to warm to rt and stirred for 16 h. The reaction mixture was diluted with DCM (50 mL) and washed with NaHCO_3_ (satd. aq. 50 mL). The aqueous layer was further extracted with DCM (2×30 mL). The combined organic layer was concentrated under reduced pressure. The material was purified by column chromatography (80 g silica, 0-80% EtOAc in heptane) to afford the title compound **50** (4.37 g, 9.47 mmol, 53%). MS (ES+): *m/z* 462 [M+H]^+^.

4-methoxy-N-(4-(piperazin-1-ylmethyl)phenyl)benzenesulfonamide (**51**)

To a solution of tert-butyl 4-[[4-[(4-methoxyphenyl)sulfonylamino]phenyl]methyl]piperazine-1-carboxylate (**50**, 4.37 g, 9.47 mmol)  in DCM (100 mL) was added TFA (3.6 mL, 47.3 mmol). The reaction was stirred a rt for 4 h. Further TFA was added (3.6 mL, 47.3 mmol) and the reaction stirred for 16 h. The reaction mixture was diluted with MeOH (25 mL) and purified by SCX-2 cartridge (2×20 g (material split into 2 portions)) washing with MeOH before eluting compound with 7 N NH_3_/MeOH. The NH_3_/MeOH fraction was concentrated under reduced pressure. The material was purified by column chromatography (40 g silica, 0-16% 7 N NH_3_/MeOH in DCM) to afford the title compound **51** as a pale yellow solid (2.9 g, 8.02 mmol, 85%). MS (ES+): *m/z* 362 [M+H]^+^.

N-[4-[[4-(3-chloro-4-methoxy-benzoyl)piperazin-1-yl]methyl]phenyl]-4-methoxy-benzenesulfonamide (**13**)

To a solution of 3-chloro-4-methoxy-benzoic acid (57.8 mg, 0.31 mmol) in DCM (2 mL) was added  triethylamine (44.7 mg, 0.44 mmol) and T3P (225 mg, 0.35 mmol). After stirring for 15 min,  4-methoxy-N-[4-(piperazin-1-ylmethyl)phenyl]benzenesulfonamide (**51**, 80 mg, 0.22 mmol) was added and the reaction was stirred at rt for 5 h. The reaction mixture was diluted with DCM (15 mL) and washed with water (15 mL). The organic layer was separated and concentrated under reduced pressure. The material was solubilized in MeOH (2 mL) and subjected to SCX-2 cartridge (2 g) washing with MeOH before eluting compound with 7N NH_3_/MeOH. The NH_3_/MeOH fraction was concentrated under reduced pressure to afford the title compound **13** as a white solid (100 mg, 0.18 mmol, 81%). ^1^H NMR (500 MHz, DMSO-d_6_) δ 10.07 (br s, 1H), 7.70 – 7.65 (m, 2H), 7.45 – 7.42 (m, 1H), 7.36 – 7.33 (m, 1H), 7.20 – 7.13 (m, 3H), 7.06 – 7.01 (m, 4H), 3.89 (s, 3H), 3.78 (s, 3H), 3.60 – 3.27 (m, 4H), 3.38 (s, 2H), 2.38 – 2.25 (m, 4H) .; ^13^C NMR (126 MHz, DMSO-d_6_) δ 167.8, 162.8, 155.8, 137.2, 133.7, 131.7, 130.1, 129.3, 127.9, 121.3, 120.3, 114.8, 112.9, 61.6, 56.7, 56.0, 52.8. Note, one aromatic carbon peak missing, three piperazine carbon peaks missing.; HRMS (ES^+^): m/z [M + H]^+^ calcd for C_26_H_28_N_3_O_5_SCl, 530.1516; found, 530.1530.

tert-butyl (1-(4-nitrobenzyl)piperidin-4-yl)carbamate (**52**)

To a solution of 4-nitrobenzaldehyde (500 mg, 3.31 mmol) in DCM (10 mL) stirred under nitrogen at 0°C was added tert-butyl piperidin-4-ylcarbamate (530 mg, 2.65 mmol). After stirring the resulting mixture for 1 h sodium triacetoxyborohydride (1.96 g, 9.27 mmol) was added at 0°C. The reaction mixture was stirred at rt for 22 h. The reaction mixture was diluted with DCM (25 mL) and washed with NaOH (2 M aq. 25 mL). The organic layer was separated, dried over anh. Na_2_SO_4_, filtered and concentrated under reduced pressure. The material was purified by column chromatography (25 g silica, 30% EtOAc in pet. ether) to afford the title compound **52** as an orange solid (740 mg, 66%). MS (ES+): *m/z* (%) 336 (100) [M+H]^+^.

tert-butyl (1-(4-aminobenzyl)piperidin-4-yl)carbamate (**53**)

To a solution of tert-butyl (1-(4-nitrobenzyl)piperidin-4-yl)carbamate (**52**, 740 mg, 2.21 mmol) in THF (10 mL) and water (10 mL) stirred under nitrogen at 0°C was added ammonium chloride (590 mg, 11.0 mmol), followed by zinc (721 mg, 11.0 mmol). The reaction mixture was stirred at rt for 16 hr. The reaction mixture was filtered through celite, the filtrate was diluted with EtOAc (25 mL) and washed with water (30 mL). The organic layer was separated, dried over anh. Na_2_SO_4_, filtered and concentrated under reduced pressure to afford the title compound **53** as a yellow solid (680 mg, 91%). This compound was used in subsequent steps without further purification. MS (ES+): *m/z* (%) 306 (100) [M+H]^+^.

tert-butyl (1-(4-((4-methylphenyl)sulfonamido)benzyl)piperidin-4-yl)carbamate (**54**)

To a stirred solution of tert-butyl (1-(4-aminobenzyl)piperidin-4-yl)carbamate (**53**, 680 mg, 2.23 mmol) in DCM (13 mL) under nitrogen at 0°C was added 4-methylbenzenesulfonyl chloride (424 mg, 2.23 mmol) followed by triethylamine (631 mg, 6.23 mmol). The reaction mixture was stirred at rt for 5 h. The reaction mixture was diluted with DCM (30 mL) and washed with water (25 mL). The organic layer was separated, dried over anh. Na_2_SO_4_, filtered and concentrated under reduced pressure. The material was purified by column chromatography (25 g silica, 5% MeOH in DCM) to afford the title compound **54** as a pale yellow solid (650 mg, 1.41 mmol, 62%). MS (ES+): *m/z* (%) 460 (100) [M+H]^+^.

N-(4-((4-aminopiperidin-1-yl)methyl)phenyl)-4-methylbenzenesulfonamide,Trifluoroacetic acid salt (**55**)

To a solution of tert-butyl (1-(4-((4-methylphenyl)sulfonamido)benzyl)piperidin-4-yl)carbamate (**54**, 650 mg, 1.41 mmol) in DCM (8 mL) stirred under nitrogen at 0°C was added TFA (0.55 mL, 7.07 mmol). The reaction mixture was stirred at rt for 4 h. The reaction mixture was concentrated under reduced pressure to afford the title compound **55** (660 mg, 98%). MS (ES+): *m/z* (%) 360 (100) [M+H]^+^. This compound was used in subsequent steps without further purification.

3-chloro-N-(1-(4-((4-methylphenyl)sulfonamido)benzyl)piperidin-4-yl)benzamide (**37**)

To a solution of 3-chlorobenzoic acid (0.22 g, 1.39 mmol), in DCM (10 mL) stirred under nitrogen at 0°C was added HATU (0.64 g, 1.67 mmol) followed by N,N-diisopropylethylamine (0.73 mL, 4.18 mmol). After stirring the resulting mixture for 10 min, N-(4-((4-aminopiperidin-1-yl)methyl)phenyl)-4-methylbenzenesulfonamide,Trifluoroacetic acid salt (**55**, 0.66 g, 1.39 mmol) was added. The reaction mixture was stirred at rt for 16 h. The reaction mixture was diluted with DCM (30 mL) and washed with water (25 mL). The organic layer was separated, dried over anh. Na_2_SO_4_, filtered and concentrated under reduced pressure. The material was purified by column chromatography (25 g silica, 5% MeOH in DCM) then prep-HPLC (5-95% MeCN in water (+0.1% formic acid)) to afford the title compound **37** as a white solid (0.17 g, 0.306 mmol, 22% yield). ^1^H-NMR (500 MHz, DMSO-d_6_) δ 9.81 (br s, 1H), 8.34 (d, *J*=7.60 Hz, 1H), 8.17 (s, 1H), 7.87 (d, *J*=1.65 Hz, 1H), 7.79 (d, *J*=7.85 Hz, 1H), 7.63 (d, *J*=8.25 Hz, 2H), 7.58 (dd, *J*=1.05, 8.00 Hz, 1H), 7.49 (t, *J*=7.88 Hz, 1H), 7.34 (d, *J*=8.10 Hz, 2H), 7.15 (d, *J*=8.40 Hz, 2H), 7.04 (d, *J*=8.40 Hz, 2H), 3.74 (m, 2H), 2.75 (d, *J*=11.65 Hz, 2H), 2.33 (s, 3H), 1.98 (t, *J*=10.95 Hz, 2H), 1.75 (d, *J*=10.05 Hz, 2H), 1.54 (m, *J*=3.54, 11.88 Hz, 2H). ; ^13^C NMR (126 MHz, DMSO-d_6_) δ 164.64, 163.69, 143.62, 137.31, 137.18, 137.01, 134.58, 133.54, 131.35, 130.68, 130.10, 129.99, 127.50, 127.15, 126.56, 120.44, 66.47, 61.83, 58.68, 52.53, 47.53, 31.75, 21.41.; HRMS (ES^+^): m/z [M + H]^+^ calcd for C_26_H_29_N_3_O_3_SCl, 498.1618; found, 498.1633.

N-(4-formylphenyl)-4-methoxybenzenesulfonamide (**56**)

A mixture of 4-methoxybenzenesulfonamide (1.34 g, 7.13 mmol), 4-bromobenzaldehyde (1.20 g, 6.49 mmol), Potassium phosphate tribasic (2.07 g, 9.73 mmol) and XPhos (0.93 g, 1.95 mmol) in 1,4-Dioxane (20 mL) was degassed with nitrogen. Tris(dibenzylideneacetone)dipalladium(0) (0.59 g, 0.65 mmol) was then added and the reaction was heated at 100°C for 16 h. The reaction was filtered through celite with MeOH (150 mL) and subsequently concentrated under reduced pressure. The residue was partitioned between EtOAc (200 mL) and NaHCO_3_ (satd. aq. 100 mL). The organic phase was extracted, dried over anh. Na_2_SO_4_, filtered and concentrated under reduced pressure. Material purified by column chromatography (100 g silica, 0-25% EtOAc in cyclohexane) to afford the title compound **56** as a pale yellow solid (1.42 g, 75%). MS (ES-): *m/z* (%) 290 (100) [M-H]^+^.

tert-butyl (1S,4S)-5-(4-((4-methoxyphenyl)sulfonamido)benzyl)-2,5-diazabicyclo[2.2.1]heptane-2-carboxylate (**58**)

To a solution of N-(4-formylphenyl)-4-methoxybenzenesulfonamide (**56**, 100 mg, 0.34 mmol) in THF (1.5 mL) was added tert-butyl 3,8-diazabicyclo[3.2.1]octane-8-carboxylate (73 mg, 0.34 mmol). The reaction was stirred at rt for 30 min. Sodium triacetoxyborohydride (146 mg, 0.69 mmol) was then added and the reaction was stirred for further 3 h. NaHCO_3_ (satd. aq. 10 mL) was added and the product was extracted with EtOAc (2×10 mL). The organic extract was dried over anh. MgSO_4_, filtered and concentrated under reduced pressure. Material purified by column chromatography (5 g silica, 0-50% EtOAc in cyclohexane) to afford the title compound **58** as a white solid (0.15 g, 90%). MS (ES+): *m/z* (%) 488 (100) [M+H]^+^.

N-(4-(((1S,4S)-2,5-diazabicyclo[2.2.1]heptan-2-yl)methyl)phenyl)-4-methoxybenzenesulfonamide (**59**)

To a solution of tert-butyl 3-(4-((4-methoxyphenyl)sulfonamido)benzyl)-3,8-diazabicyclo[3.2.1]octane-8-carboxylate (**58**, 150 mg, 0.31 mmol) in MeOH (1.5 mL) was added 4 M HCl in dioxane (0.3 mL, 1.2 mmol). The reaction was stirred at rt for 16 h and subsequently concentrated under reduced pressure. The residue was purified by SCX-2 cartridge (10 g) loading and washing with MeOH before eluting compound with 7 N NH_3_/MeOH. The NH_3_/MeOH fraction was concentrated under reduced pressure to afford the title compound **59** as a colourless oil (0.12 g, quant.). MS (ES+): *m/z* (%) 388 (100) [M+H]^+^.

N-(4-(((1S,4S)-5-(3-chlorobenzoyl)-2,5-diazabicyclo[2.2.1]heptan-2-yl)methyl)phenyl)-4-methoxybenzenesulfonamide (**39**)

A solution of 3-chlorobenzoic acid (34 mg, 0.22 mmol) , 1-hydroxybenzotriazole hydrate (40 mg, 0.26 mmol), N-(3-dimethylaminopropyl)-N'-ethylcarbodiimide hydrochloride (50 mg, 0.26 mmol) and triethylamine (0.10 mL, 0.72 mmol) in DCM (2mL) was stirred for 30 min at rt before addding N-(4-(((1S,4S)-2,5-diazabicyclo[2.2.1]heptan-2-yl)methyl)phenyl)-4-methoxybenzenesulfonamide (**59**, 80 mg, 0.21 mmol) . The mixture was stirred at rt for 18 h. Water (10 mL) was added and the product was extracted with DCM (3×50 mL). The organic layer was dried over anh. Na_2_SO_4_, filtered and concentrated under reduced pressure. The material was purified by column chromatography (5 g silica, 50% EtOAc/EtOH (3/1) in cyclohexane) to afford title compound **39** as a white solid which was dried in an oven at 40˚C (50 mg, 46%).^1^H-NMR (500 MHz, DMSO-d_6_) δ 10.05 (br s, 1H), 7.68 (m, 2H), 7.58 - 7.39 (m, 4H), 7.17 (m, 2H), 7.03 (m, 4H), 3.79 (m, 3H), 3.67 - 3.33 (m, 6H), 2.74 (m, 1H), 2.60 (m, 1H), 1.76 (m, 2H).; ^13^C NMR (126 MHz, DMSO-d_6_) δ 167.10, 166.38, 162.83, 139.14, 138.73, 136.98, 135.66, 133.65, 133.58, 131.81, 131.78, 130.89, 130.82, 130.45, 130.16, 129.45, 129.32, 127.45, 127.31, 126.24, 126.05, 120.44, 120.40, 114.81, 114.78, 61.19, 60.86, 60.32, 60.07, 59.20, 57.59, 57.49, 57.31, 56.08, 53.84, 50.45, 36.06, 34.35.; HRMS (ES^+^): m/z [M + H]^+^ calcd for C_26_H_27_N_3_O_4_SCl, 512.1411; found, 512.1425.

tert-butyl 4-(4-nitrophenethyl)piperazine-1-carboxylate (**60**)

To a solution of 1-(2-bromoethyl)-4-nitrobenzene (400 mg, 1.74 mmol) in MeCN (5 mL) was added K_2_CO_3_ (481 mg, 3.48 mmol) and tert-butyl piperazine-1-carboxylate (389 mg, 2.09 mmol). The reaction was stirred at rt for 16 h. The mixture was partitioned between EtOAC (20 mL) and water (20 mL). The phases were separated. The aqueous phase was extracted with EtOAc (3×10 mL). The combined organic layer was dried over anh. Na_2_SO_4_, filtered and concentrated under reduced pressure. The material was purified by column chromatography (25 g silica, 0-40% EtOAc in cyclohexane) to afford the title compound **60** (240 mg, 0.72 mmol, 41%). MS (ES+): *m/z* (%) 336 (100) [M+H]^+^.

1-(4-nitrophenethyl)piperazine (**61**)

To a solution of tert-butyl 4-(4-nitrophenethyl)piperazine-1-carboxylate (**60**, 240 mg, 0.72 mmol) in MeOH (1 mL), 4 M HCl in dioxane (0.89 mL, 3.58 mmol) was added. The reaction was stirred at rt for 24 h. The reaction was concentrated under reduced pressure to afford the title compound **61** (203 mg, quant.). MS (ES+): *m/z* (%) 236 (100) [M+H]^+^. This compound was used in subsequent steps without further purification.

(3-chlorophenyl)(4-(4-nitrophenethyl)piperazin-1-yl)methanone (**62**)

To a solution of 3-chlorobenzoic acid (149 mg, 0.95 mmol), 1-hydroxybenzotriazole hydrate (172 mg, 1.12 mmol), N-(3-dimethylaminopropyl)-N'-ethylcarbodiimide hydrochloride (215 mg, 1.12 mmol) and 1-(4-nitrophenethyl)piperazine (**61**, 203 mg, 0.86 mmol) in DCM (3 mL) was added N,N-diisopropylethylamine (0.45 mL, 2.59 mmol). The reaction was stirred at rt for 72 h. The reaction mixture was poured into water (10 mL) and the product was extracted with DCM (3×10 mL). The organic layers were combined, dried over anh. Na_2_SO_4_, filtered and concentrated under reduced pressure. The material was purified by column chromathography (12 g silica, 0-30% EtOAc/EtOH in cyclohexane) to afford the title compound **62** as an off white solid (201 mg, 62%). MS (ES+): *m/z* (%) 374 (100) [M+H]^+^.

(4-(4-aminophenethyl)piperazin-1-yl)(3-chlorophenyl)methanone (**63**)

To a solution of (3-chlorophenyl)(4-(4-nitrophenethyl)piperazin-1-yl)methanone (**62**, 181 mg, 0.48 mmol) in acetic acid ( 3 mL) was added zinc (317 mg, 4.84 mmol). The reaction was stirred at rt for 2.5 h. The reaction mixture was filtered through celite and subsequently concentrated under reduced pressure. The residue was partitioned between K_2_CO_3_ (satd. aq. 15 mL) and DCM (15 mL). The organic layer was dried over anh. Na_2_SO_4_, filtered and concentrated under reduced pressure to afford the title compound **63** (149 mg, 89%). MS (ES+): *m/z* (%) 344 (100) [M+H]^+^.

N-(4-(2-(4-(3-chlorobenzoyl)piperazin-1-yl)ethyl)phenyl)-4-methoxybenzenesulfonamide (**40**)

To a solution of (4-(4-aminophenethyl)piperazin-1-yl)(3-chlorophenyl)methanone (**63**, 20 mg, 0.05 mmol), in DCM (0.5 mL) was added N,N-diisopropylethylamine (0.02 mL, 0.11 mmol). After stirring for 15 min at 0˚C, 4-methoxybenzenesulfonyl chloride (8.7 mg, 0.04 mmol) was added. The reaction was stirred at rt for 12 h. The mixture was partitioned between EtOAc (20 mL) and water (20 mL). The phases were separated. The aqueous phase was extracted with EtOAc (3×10 mL). The combined organic layer was dried over anh. Na_2_SO_4_, filtered and concentrated under reduced pressure. The material was purified by column chromatography (4 g, silica 0-5% MeOH in DCM) to afford the title compound **40** as a white solid (10 mg, 36%).; ^1^H NMR (500 MHz, DMSO-d_6_) δ 9.98 (br s, 1H), 7.66 (m, 2H), 7.49 (m, 2H), 7.44 (m, 1H), 7.34 (d, 1H), 7.05 (m, 4H), 6.97 (d, *J* = 7.5 Hz, 2H), 3.79 (s, 3H), 3.59 (br s, 2H), 3.27 (br s, 2H), 2.63 (m, 2H), 2.49 – 2.37 (m, 6H).; ^13^C NMR (126 MHz, DMSO-d_6_) δ 170.03, 167.68, 162.77, 138.55, 136.30, 133.64, 130.91, 129.85, 129.70, 129.29, 127.09, 125.86, 120.68, 114.77, 59.62, 56.07, 32.27.; HRMS (ES^+^): m/z [M + H]^+^ calcd for C_26_H_29_N_3_O_4_SCl, 514.1567; found, 514.1536 (-6 ppm).

ethyl 5-((4-methoxyphenyl)sulfonamido)-1,3,4-oxadiazole-2-carboxylate (**64**)

To a solution of ethyl 5-amino-1,3,4-oxadiazole-2-carboxylate (1.0 g, 6.36 mmol) in DMF (40 mL) at 0°C was added NaH (0.51 g, 12.7 mmol). The reaction was stirred for 25 min before adding 4-methoxybenzenesulfonyl chloride (1.45 g, 7.0 mmol). The reaction was stirred at 0°C to rt for 2 h. The reaction was quenched by dropwise addition of 1 M HCl (aq) and partitioned between 1 M HCl (aq. 60 mL) and EtOAc (80 mL), organic extract collected and aqueous re-extracted with EtOAc (2×50 mL). The combined organic layer was washed with brine (70 mL), collected and concentrated under reduced pressure. The material was purified by column chromatography (40 g silica, 0-100% EtOAc in heptane) to afford the title compound **64** as a white solid (0.77 g, 2.34 mmol, 37%). MS (ES+): *m/z* 328 [M+H]^+^.

N-(5-formyl-1,3,4-oxadiazol-2-yl)-4-methoxy-benzenesulfonamide (**65**)

To a solution of ethyl 5-[(4-methoxyphenyl)sulfonylamino]-1,3,4-oxadiazole-2-carboxylate (**64**, 250 mg, 0.76 mmol) in THF (10 mL) at -78^o^C was added LiAlH_4_, 2M in THF (0.46 mL, 0.92 mmol) dropwise. The reaction was stirred for 1 h. The reaction was quenched by dropwise addition of water, before adding 1M HCl (aq. 30 mL). The aqueous was extracted with EtOAc (3×30 mL). The combined organic layer was washed with brine (60 mL), collected and concentrated under reduced pressure. The material was purified by column chromatography (12g silica, 0-100% EtOAc in heptane) to afford the title compound **65** (110 mg, 0.21 mmol, 28%). Note, material was a 1:1 mixture of aldehyde product and fully reduced alcohol product, this mixture was used in subsequent steps without further purification.

N-[5-[[4-(3-chlorobenzoyl)piperazin-1-yl]methyl]-1,3,4-oxadiazol-2-yl]-4-methoxy-benzenesulfonamide (**41**)

To a solution of N-(5-formyl-1,3,4-oxadiazol-2-yl)-4-methoxy-benzenesulfonamide (**65**, 60 mg, 0.21 mmol)  and  (3-chlorophenyl)-piperazin-1-yl-methanone (52 mg, 0.23 mmol)  in DCM (2.5 mL) was added a spoonful of anh. MgSO_4_. The reaction was stirred at rt for 2 h before adding sodium triacetoxyborohydride (90 mg, 0.42 mmol). The reaction was stirred at rt for 16 h. The reaction mixture was diluted with DCM (15 mL) and washed with NaHCO_3_ (satd. aq.) (20 mL). The organic extract was collected and concentrated under reduced pressure. The material was solubilized in MeOH (5 mL) and subjected to SCX-2 cartridge (2 g) washing with MeOH before eluting compound with 7N NH_3_/MeOH. The NH_3_/MeOH fraction was concentrated under reduced pressure. The material was purified by column chromatography (12 g silica, 0-20% 7 N NH_3_/MeOH in DCM) then solubilized in MeOH (5 mL) and subjected to SCX-2 cartridge (2 g) washing with MeOH before eluting compound with 7N NH_3_/MeOH. The NH_3_/MeOH fraction was concentrated under reduced pressure to afford the title compound **41** as a pale yellow solid (10 mg, 0.02 mmol, 9%). ^1^H-NMR (500 MHz, DMSO-d_6_) δ 13.58 (br s, 1H), 7.82 (d, *J*=8.87 Hz, 2H), 7.53 (m, 1H), 7.48 (t, *J*=7.77 Hz, 1H), 7.44 (s, 1H), 7.34 (d, *J*=7.50 Hz, 1H), 7.06 (d, *J*=8.90 Hz, 2H), 3.81 (s, 3H), 3.68 (s, 2H), 3.61 (br s, 2H), 3.31 (m, 2H), 2.60 – 2.40 (m, 4H). ^13^C NMR (126 MHz, DMSO-d_6_) δ 167.79, 162.45, 156.21, 138.37, 134.87, 133.68, 130.92, 129.92, 128.76, 127.17, 125.95, 114.49, 56.06, 51.93, 51.35, 47.42. Note, piperazine carbon peaks are weak and broad.; HRMS (ES^+^): m/z [M + H]^+^ calcd for C_21_H_23_N_5_O_5_SCl , 492.1108; found, 492.1125.

tert-butyl 4-((5-nitropyridin-2-yl)methyl)piperazine-1-carboxylate (**66**)

A solution of 5-nitropicolinaldehyde (2.0 g, 13.2 mmol) and tert-butyl piperazine-1-carboxylate (1.96 g, 10.5 mmol) in DCM (50 mL) was stirred under nitrogen at rt for 1 h before adding sodium triacetoxyborohydride (8.92 g, 42.1 mmol) portionwise over 5 min. The reaction was stirred at rt for 16 h. Ice cold water (200 mL) was added to the reaction mixture and stirred for 5 min before adding DCM (200 mL). The organic layer was separated, aqueous layer was extracted with DCM (2×200 mL). The combined organic layer was washed with brine (200 mL), dried over anh. Na_2_SO_4_, filtered and concentrated under reduced pressure. The residue was washed with pentane (50 mL) and the solids dried under reduced presssure to afford the title compound **66** (3.20 g, 68%). MS (ES+): *m/z* (%) 323 (100) [M+H]^+^.This compound was used in subsequent steps without further purification.

tert-butyl 4-((5-aminopyridin-2-yl)methyl)piperazine-1-carboxylate (**67**)

To a solution of tert-butyl 4-((5-nitropyridin-2-yl)methyl)piperazine-1-carboxylate (**66**, 3.20 g, 9.93 mmol) in THF (25 mL) and water (25 mL) stirred under nitrogen at rt was added ammonium chloride (2.65 g, 49.6 mmol) followed by zinc (3.25 g, 49.6 mmol) portionwise over 1 min. The reaction was stirred at rt for 24 h. The reaction mixture was filtered, the solid residue was washed with EtOAc (500 mL) and the filtrate was partitioned between water (200 ml) and EtOAc (2×200 mL). The combined organic layer was washed with brine (150 mL), dried over anh. Na_2_SO_4_, filtered and concentrated under reduced pressure. The residue was triturated with pentane (100 mL) and concentrated under reduced pressure to afford the title compound **67** (2.9 g, 96%). MS (ES+): *m/z* (%) 293 (100) [M+H]^+^. This compound was used in subsequent steps without further purification.

tert-butyl 4-((5-((4-methoxyphenyl)sulfonamido)pyridin-2-yl)methyl)piperazine-1-carboxylate (**68**)

To a solution of tert-butyl 4-((5-aminopyridin-2-yl)methyl)piperazine-1-carboxylate (**67**, 1.0 g, 3.42 mmol) in pyridine (5 mL) stirred under nitrogen at 0°C was added 4-methoxybenzenesulfonyl chloride (707 mg, 3.42 mmol). The reaction was stirred at 0 °C to rt for 1 h. Ice cold water (50 mL) was added to reaction mixture, stirred for 5 min, then EtOAc (100 mL) was added. The organic layer was separated, aqueous layer was extracted with EtOAc (2×50 mL). The combined organic layer was washed with brine (150 mL), dried over anh. Na_2_SO_4_, filtered and concentrated under reduced pressure. The material was purified by column chromatography (25 g silica, 80% EtOAc in pet. ether) to afford the title compound **68** as a pale pink solid (1.25 g, 2.59 mmol, 77%). MS (ES+): *m/z* (%) 463 (100) [M+H]^+^.

4-methoxy-N-(4-(piperazin-1-ylmethyl)phenyl)benzenesulfonamide Trifluoroacetate (**69**)

To a solution of tert-butyl 4-((5-((4-methoxyphenyl)sulfonamido)pyridin-2-yl)methyl)piperazine-1-carboxylate (**68**, 1.25 g, 2.59 mmol) in DCM (10 mL) stirred under nitrogen at 0°C was added trifluoroacetic acid (2.0 mL, 25.9 mmol) dropwise over 1 min. The reaction mixture was stirred at 0 °C to rt for 5 h and subsequently concentrated under reduced pressure to afford the title compound **69** (1.6 g, 1.87 mmol, 72%). MS (ES+): *m/z* (%) 363 (100) [M+H]^+^. This compound was used in subsequent steps without further purification.

N-(6-((4-(3-chlorobenzoyl)piperazin-1-yl)methyl)pyridin-3-yl)-4-methoxybenzenesulfonamide (**44**)

To a solution of 4-methoxy-N-(6-(piperazin-1-ylmethyl)pyridin-3-yl)benzenesulfonamide (**69**, 300 mg, 0.83 mmol) and 3-chlorobenzoic acid (168 mg, 1.08 mmol) in DCM (10 mL) stirred under nitrogen at rt was added N,N-diisopropylethylamine (0.72 mL, 4.14 mmol) followed by T3P (0.79 mL, 1.24 mmol). The reaction mixture was stirred at rt for 16 h. The reaction mixture was diluted with DCM (30 mL) and washed with water (25 mL). The organic layer was separated, aqueous layer was extracted with DCM (2×30 mL). The combined organic layer was washed with brine (30 mL), dried over anh. Na_2_SO_4_, filtered and concentrated under reduced pressure. The material was purified by column chromatography (10 g silica, 5-6% MeOH in DCM). The material was further purified by dissolving in EtOAc (10 mL) and washing with NaHCO_3_ (satd. aq. 2×20 mL). The organic layer was washed with brine (10 mL), dried over anh. Na_2_SO_4_, filtered and concentrated under reduced pressure to afford the title compound **44** (110 mg, 26%).; ^1^H-NMR (500 MHz, DMSO-d_6_) δ 10.31 (br s, 1H), 8.18 (d, *J* = 2.4 Hz, 1H), 7.68 (d, *J* = 8.9 Hz, 2H), 7.52 (m, 1H), 7.47 (m, 2H), 7.43 (s, 1H), 7.33 (m, 1H), 7.06 (d, *J* = 8.9 Hz, 2H), 3.80 (s, 3H), 3.60 (br s, 2H), 3.53 (s, 2H), 2.39 (m, 4H). Note, some aliphatic signals hidden under water peak, 3.2 ppm. ^13^C NMR (126 MHz, DMSO-d_6_) δ 167075, 163.05, 154.02, 141.46, 138.51, 133.75, 131.33, 130.91, 129.87, 129.38, 128.52, 127.16, 125.94, 123.57, 114.97, 63.25, 56.13, 53.16, 52.76, 47.53, 42.05. Note, piperazine carbon peaks are weak and broad.; HRMS (ES^+^): m/z [M + H]^+^ calcd for C_24_H_26_N_4_O_4_SCl, 501.1363; found, 501.1383.

methyl 5-aminopyrimidine-2-carboxylate (**70**)

To a solution of methyl 5-((diphenylmethylene)amino)pyrimidine-2-carboxylate (2.0 g, 6.30 mmol) in MeOH (25 mL) stirred under nitrogen at rt was added neat HCl (5.32 mL, 63.0 mmol) dropwise. The reaction was stirred at rt for 16 h and subsequently concentrated under reduced pressure. The residue was basified with NH_3_ (10% aq. 12 mL) (to pH 8-9 approximately) and extracted into 10% MeOH in DCM (7×20 mL). The combined organic layer was dried over anh. Na_2_SO_4_, filtered and concentrated under reduced pressure. The residue was triturated with DCM and pentane (1:5, 3×30 mL) and dried under vacuo to afford the title compound **70** as a pale brown solid (0.65 g, 3.90 mmol, 62%). MS (ES+): *m/z* (%) 154 (100) [M+H]^+^.

methyl 5-((diphenylmethylene)amino)pyrimidine-2-carboxylate (**72**)

A suspension of methyl 5-bromopyrimidine-2-carboxylate (**70**, 2.0 g, 9.22 mmol), diphenylmethanimine (1.67 g, 9.22 mmol), BINAP (0.57 g, 0.92 mmol) and caesium carbonate (3.90 g, 12.0 mmol) in Toluene (20 mL) was stirred under nitrogen at rt. This was degassed with nitrogen for 15 min before adding palladium (II) acetate (0.21 g, 0.92 mmol) in one charge. The reaction was stirred at 100°C for 5 h. The reaction mixture was diluted with EtOAc (40 mL) and washed with water (35 mL). The aqueous layer was further extracted with EtOAc (2×35 mL). The combined organic layer was dried over anh. Na_2_SO_4_, filtered and concentrated under reduced pressure. The material was purified by column chromatography (100g silica, 35% EtOAc in pet. ether) to afford the title compound **72** as yellow solid (2.0 g, 5.29 mmol, 57%). MS (ES+): *m/z* (%) 318 (100) [M+H]^+^.

methyl 5-((4-methoxyphenyl)sulfonamido)pyrimidine-2-carboxylate (**73**)

A solution of methyl 5-aminopyrimidine-2-carboxylate (**72**, 590 mg, 3.85 mmol) and 4-methoxybenzenesulfonyl chloride (1.59 g, 7.71 mmol) in pyridine (8 mL) was stirred under nitrogen at 60°C for 16 h. The reaction mixture was concentrated under reduced pressure, diluted with EtOAc (25 mL) and washed with water (25 mL). The aqueous layer was extracted with EtOAc (15 mL). The combined organic layer was dried over anh. Na_2_SO_4_, filtered and concentrated under reduced pressure. The material was purified by trituration with EtOAc and pentane (1:3, 2×20 mL); then with diethyl ether and pentane (1:3, 2×20 mL) to afford the title compound **73** as a pale brown solid (650 mg, 50%). MS (ES+): *m/z* (%) 324 (100) [M+H]^+^.

N-(2-(hydroxymethyl)pyrimidin-5-yl)-4-methoxybenzenesulfonamide (**75**)

To a solution of methyl 5-((4-methoxyphenyl)sulfonamido)pyrimidine-2-carboxylate (**73**, 250 mg, 0.77 mmol) in EtOH (4 mL), diethyl ether (4 mL) and THF (4 mL) stirred under nitrogen at 0°C lithium borohydride (2 M in THF) (1.16 mL, 2.32 mmol) was added dropwise over 5 min. The reaction was stirred at rt for 20 h. Further lithium borohydride (2M in THF) (1.93 mL, 3.87 mmol) was added and the mixture was stirred at rt for 16 h. The reaction was quenched with NH_4_Cl (sat. aq. 15 mL) at 0°C and extracted with 10 % MeOH in DCM (3×20 mL). The combined organic layer was dried over anh. Na_2_SO_4_, filtered and concentrated under reduced pressure. The material was purified by column chromatography (10 g silica, 0-7% MeOH in DCM) to afford the title compound **75** as colorless oil (110 mg, 47%). MS (ES+): *m/z* (%) 296 (100) [M+H]^+^.

N-(2-(bromomethyl)pyrimidin-5-yl)-4-methoxybenzenesulfonamide (**77**)

To a solution of N-(2-(hydroxymethyl)pyrimidin-5-yl)-4-methoxybenzenesulfonamide (**75**, 110 mg, 0.37 mmol) in DCM (10 mL) stirred under nitrogen at 0°C, neat phosphorus tribromide (0.11 mL, 1.12 mmol) was added dropwise over 1 min. The reaction was stirred at rt for 1 h. The reaction was quenched with NaHCO_3_ (10% aq. 15 mL) at 0°C and extracted with 10% MeOH in DCM (3×20 mL). The combined organic layer was dried over anh. Na_2_SO_4_, filtered and concentrated under reduced pressure to afford the title compound **77** (90 mg, 59%). MS (ES+): *m/z* (%) 358 (100) [M+H]^+^. This compound was used in subsequent steps without further purification.

N-(2-((4-(3-chlorobenzoyl)piperazin-1-yl)methyl)pyrimidin-5-yl)-4-methoxybenzenesulfonamide (**43**)

To a solution of (3-chlorophenyl)(piperazin-1-yl)methanone (57 mg, 0.25 mmol) and N-(2-(bromomethyl)pyrimidin-5-yl)-4-methoxybenzenesulfonamide (**77**, 90 mg, 0.25 mmol) in DMF (3 mL), stirred under nitrogen at 0°C, potassium carbonate (694 mg, 0.50 mmol) was added. The reaction was stirred at rt for 16 h. The reaction mixture was diluted with EtOAc (15 mL) and washed with water (15 mL). The aqueous layer was further extracted with EtOAc (2×15 mL). The combined organic layer was concentrated under reduced pressure. The material was purified by column chromatography (10 g silica, 6% MeOH in DCM) then prep-HPLC (5-95% MeCN in water (+0.1% formic acid)) to afford the title compound **43** as a white solid (27 mg, 21%). ^1^H-NMR (500 MHz, DMSO-d_6_) δ 10.60 (br s, 1H), 8.40 (s, 2H), 7.65 (d, *J* = 8.9, 2H), 7.44 (m, 1H), 7.39 (t, *J* = 7.8 Hz, 1H), 7.35 (s, 1H), 7.53 (d, *J* = 7.5 Hz, 1H), 7.01 (d, *J* = 8.9 Hz, 2H), 3.73 (s, 3H), 3.64 (br s, 2H), 3.52 (br s, 2H), 2.55 – 2.31 (m, 4H). Note, two protons hidden under solvent peak.; ^13^C NMR (126 MHz, DMSO-d_6_) δ 167.76, 163.29, 148.91, 138.42, 133.67, 132.12, 130.92, 130.78, 129.50, 127.17, 125.96, 115.16, 63.49, 56.19, 52.94, 52.50, 47.30. Note, piperazine carbon peaks are weak and broad.; HRMS (ES^+^): m/z [M + H]^+^ calcd for C_23_H_25_N_5_O_4_SCl, 502.1316; found, 502.1323.

- 1. **Synthesis – Plate based**

The selected acids were solubilised and plated into a Griener 96-well 651201 V bottom polypropylene plate (33 mM in DMSO, 7 µL, 1 eq.) using a Tecan Fluent liquid handler. A solution of CDI (33 mM in DMSO, 14 µL, 2 eq.) was added and the reactions left to stand for 1 h. Note, some acids were insoluble at the required concentration and so were activated with CDI before plating. Finally, the template (33mM in DMSO, 7 µL, 1 eq.) was added and the plate sealed and left to stand for 12 h.

- 1. **Final compound experimental data**

N-(4-((4-(3-chlorobenzoyl)piperazin-1-yl)methyl)phenyl)-4-methylbenzenesulfonamide (**1**)

(480 mg, 0.94 mmol, 44%); ^1^H NMR (500 MHz, DMSO-d_6_) δ 10.14 (br s, 1H), 7.63 (d, *J* = 8.2, 2H), 7.52 – 7.50 (m, 1H), 7.48 – 7.44 (m, 1H), 7.42 (s, 1H), 7.34 – 7.32 (m, 3H), 7.15 (d, *J* = 8.4 Hz, 2H), 7.03 (d, *J* = 8.4 Hz, 2H), 3.60 – 3.56 (m, 2H), 3.38 (s, 2H), 3.29 – 3.25 (m, 2H), 2.35 – 2.28 (m, 4H), 2.33 (s, 3H) .; ^13^C NMR (126 MHz, DMSO-d_6_) δ 167.7, 143.6, 138.5, 137.3, 137.1, 133.9, 133.7, 130.9, 130.11, 130.10, 129.9, 127.1, 125.9, 120.4, 61.6, 53.0, 52.5, 47.6, 42.0, 21.4. Note, one aromatic carbon peak is not observed. Note, piperazine carbon peaks are weak and broad.; HRMS (ES^+^): m/z [M + H]^+^ calcd for C_25_H_27_N_3_O_3_SCl, 484.1462; found, 484.1484.

N-(4-((4-(3-chlorobenzoyl)piperazin-1-yl)methyl)phenyl)methanesulfonamide (**2**)

(47 mg, 0.11 mmol, 52%); ^1^H NMR (500 MHz, DMSO-d_6_) δ 9.65 (br s, 1H), 7.53 – 7.51 (m, 1H), 7.49 – 7.46 (m, 1H), 7.44 – 7.43 (m, 1H), 7.35 – 7.33 (m, 1H), 7.27 (d, *J* = 8.4 Hz, 2H), 7.17 (d, *J* = 8.4 Hz, 2H), 3.63 – 3.59 (m, 2H), 3.52 (s, 2H), 3.46 (s, 2H), 2.97 (s, 3H), 2.42 – 2.34 (m, 4H).; ^13^C NMR (126 MHz, DMSO-d_6_) δ 167.8, 138.5, 137.7, 133.8, 133.7, 130.9, 130.3, 129.9, 127.2, 125.9, 120.3, 70.3, 61.7, 53.0, 52.5, 47.6, 42.1. Note, piperazine carbon peaks are weak and broad.; HRMS (ES^+^): m/z [M + H]^+^ calcd for C_19_H_23_N_3_O_3_SCl, 408.1149; found, 408.1156.

N-(4-((4-(3-chlorobenzoyl)piperazin-1-yl)methyl)phenyl)benzenesulfonamide (**3**)

(282 mg, 0.60 mmol, 78%); ^1^H NMR (500 MHz, DMSO-d_6_) δ 10.22 (br s, 1H), 7.76 – 7.74 (m, 2H), 7.62 – 7.59 (m, 1H), 7.55 – 7.50 (m, 3H), 7.48 – 7.45 (m, 1H), 7.42 (m, 1H), 7.33 – 7.32 (m, 1H), 7.15 (d, *J* = 8.4 Hz, 2H), 7.04 (d, *J* = 8.4 Hz, 2H), 3.58 – 3.57 (m, 2H), 3.39 (s, 2H), 3.29 – 3.26 (m, 2H), 2.34 – 2.39 (m, 4H).; ^13^C NMR (126 MHz, DMSO-d_6_) δ 167.7, 140.1, 138.5, 137.0, 134.0, 133.7, 133.1, 130.9, 130.3, 130.1, 129.9, 129.7, 127.14, 127.08, 125.9, 120.6, 61.6, 53.0, 52.5, 47.5, 42.0. Note, piperazine carbon peaks are weak and broad. HRMS (ES^+^): m/z [M + H]^+^ calcd for C_24_H_25_N_3_O_3_SCl, 470.1305; found, 470.1322.

N-(4-((4-(3-chlorobenzoyl)piperazin-1-yl)methyl)phenyl)-4-cyanobenzenesulfonamide (**5**)

(15 mgs, 0.03 mmol, 12%); ^1^H NMR (500 MHz, DMSO-d_6_) δ 10.47 (br s, 1H), 8.03 (d, *J* = 8.4 Hz, 2H), 7.88 (d, *J* = 8.5 Hz, 2H), 7.52 – 7.51 (m, 1H), 7.48 – 7.45 (m, 1H), 7.42 – 7.41 (m, 1H), 7.17 (d, *J* = 8.4 Hz, 2H), 7.02 (d, *J* = 8.4 Hz, 2H), 3.60 – 3.58 (m, 2H), 3.39 (s, 2H), 2.37 – 2.29 (m, 4H). Note, two piperazine protons hidden under solvent peak.; HRMS (ES^+^): m/z [M + H]^+^ calcd for C_25_H_24_N_4_O_3_SCl, 495.1258; found, 495.1268.

N-(4-((4-(3-chlorobenzoyl)piperazin-1-yl)methyl)phenyl)benzo[d][1,3]dioxole-5-sulfonamide (**6**)

(40 mg, 0.07 mmol, 20%); ^1^H NMR (500 MHz, DMSO-d_6_) δ 10.13 (br s, 1H), 7.52 – 7.50 (m, 1H), 7.48 – 7.45 (m, 1H), 7.43 – 7.42 (m, 1H), 7.33 – 7.32 (m, 1H), 7.29 – 7.27 (m, 1H), 7.28 (dd, *J* = 8.2, 1.8 Hz, 1H), 7.18 (d, *J* = 1.7 Hz, 1H), 7.15 (d, *J* = 8.4 Hz, 2H), 7.03 (d, *J* = 8.4 Hz, 2H), 7.00 (d, *J* = 8.2 Hz, 1H), 6.12 (s, 2H), 3.60 – 3.57 (m, 2H), 3.39 (s, 2H), 2.35 – 2.29 (m, 4H). ). Note, two piperazine protons hidden under solvent peak.; ^13^C NMR (126 MHz, DMSO-d_6_) δ 167.7, 151.2, 148.2, 138.5, 137.7, 133.8, 133.7, 133.5, 130.9, 130.1, 129.8, 127.1, 125.9, 122.8, 120.5, 108.6, 106.9, 102.9, 61.6, 53.1, 52.5, 47.5, 42.0. Note, piperazine carbon peaks are weak and broad.; HRMS (ES^+^): m/z [M + H]^+^ calcd for C_25_H_25_N_3_O_5_SCl, 514.1203; found, 514.1214.

N-(4-((4-(3-chlorobenzoyl)piperazin-1-yl)methyl)phenyl)-1-(tetrahydrofuran-2-yl)methanesulfonamide (**7**)

(34 mgs, 0.07 mmol, 28%); ^1^H NMR (500 MHz, DMSO-d_6_) δ 9.71 (s, 1H), 7.53 – 7.51 (m, 1H), 7.48 – 7.45 (m, 1H), 7.44 – 7.43 (m, 1H), 7.35 – 7.33 (m, 1H), 7.25 (d, *J* = 8.3 Hz, 2H), 7.17 (d, *J* = 8.4 Hz, 2H), 4.20 – 4.15 (m, 1H), 3.70 – 3.66 (m, 1H), 3.62 – 3.58 (m, 3H), 3.46 (s, 2H), 3.25 – 3.24 (m, 2H), 2.40 – 2.35 (m, 4H), 2.07 – 2.00 (m, 1H), 1.85 – 1.73 (m, 2H), 1.64 –1.57 (m, 1H). Note, two piperazine protons hidden under solvent peak.; ^13^C NMR (126 MHz, DMSO-d_6_) δ 167.8, 138.5, 137.5, 133.7, 130.9, 130.2, 129.9, 127.1, 125.9, 120.2, 73.6, 67.6, 61.7, 55.9, 53.0, 52.5, 47.6, 42.0, 31.4, 25.2. Note, one aromatic carbon peak is not observed. Note, piperazine carbon peaks are weak and broad.; HRMS (ES^+^): m/z [M + H]^+^ calcd for C_23_H_29_N_3_O_4_SCl, 478.1567; found, 478.1586.

(3-chlorophenyl)(4-(4-(1,1-dioxido-1,2-thiazinan-2-yl)benzyl)piperazin-1-yl)methanone (**8**)

(314 mg, 0.70 mmol, 84%); ^1^H NMR (500 MHz, DMSO-d_6_) δ 7.53-7.50 (m, 1H), 7.49 – 7.45 (m, 1H), 7.45 – 7.43 (m, 1H), 7.36-7.30 (m, 3H), 7.29 – 7.25 (m, 2H), 3.65 – 3.56 (m, 4H), 3.50 (s, 2H), 3.29 – 3.25 (m, 2H), 2.47 – 2.31 (m, 4H), 2.20-2.13 (m, 2H), 1.84 -1.77 (m, 2H) .; ^13^C NMR (126 MHz, DMSO-d_6_) δ 167.7, 140.0, 138.5, 137.0, 133.6, 130.9, 129.8, 129.7, 127.1, 126.9, 125.9, 61.6, 53.6, 53.2, 52.6, 47.6, 42.1, 50.5, 24.3, 24.0. Note, two piperazine protons hidden under solvent peak.; HRMS (ES^+^): m/z [M + H]^+^ calcd for C_25_H_27_N_3_O_3_SCl, 448.1462; found, 448.1453.

N-(4-((4-(3-chlorobenzoyl)piperazin-1-yl)methyl)phenyl)-4-methylpiperazine-1-sulfonamide (**9**)

(45 mg, 0.09 mmol, 30%); ^1^H NMR (500 MHz, DMSO-d_6_) δ 9.85 (br s, 1H), 7.53 – 7.50 (m, 1H), 7.49 – 7.44 (m, 1H), 7.44 – 7.42 (m, 1H), 7.35 – 7.31 (m, 1H), 7.22 (d, *J* = 8.4 Hz, 2H), 7.15 (d, *J* = 8.4 Hz, 2H), 3.68 – 3.53 (m, 2H), 3.45 (s, 2H), 3.11 - 3.05 (m, 4H), 2.46 - 2.28 (m, 4H), 2.27 - 2.20 (m, 4H), 2.10 (s, 3H) .; ^13^C NMR (126 MHz, DMSO-d_6_) δ 167.7, 138.5, 137.9, 133.6, 133.1, 130.9, 130.0, 129.8, 127.1, 125.9, 119.9, 61.7, 54.1, 53.0, 52.5, 47.6, 46.1, 45.8, 42.0. Note, two piperazine protons hidden under solvent peak.; HRMS (ES^+^): m/z [M + H]^+^ calcd for C_23_H_30_N_5_O_3_SCl, 492.1836; found, 492.1847.

N-(4-((4-benzoylpiperazin-1-yl)methyl)phenyl)-4-methoxybenzenesulfonamide (**10**)

(105 mg, 0.23 mmol, 44%); ^1^H NMR (500 MHz, DMSO-d_6_) δ 10.07 (br s, 1H), 7.70 - 7.65 (m, 2H), 7.46 – 7.40 (m, 3H), 7.38 – 7.33 (m, 2H), 7.15 (d, *J* = 8.4 Hz, 2H), 7.06 - 7.01 (m, 4H), 3.78 (s, 3H), 3.69 - 3.48 (m, 2H), 3.38 (s, 2H), 2.41 - 2.21 (m, 4H) .; ^13^C NMR (126 MHz, DMSO-d_6_) δ 169.3, 162.8, 137.2, 136.4, 133.7, 131.7, 130.1, 129.9, 129.3, 128.8, 127.3, 120.3, 114.8, 61.6, 56.1, 53.0, 52.7, 47.6, 42.0. Note, two piperazine protons hidden under solvent peak.; HRMS (ES^+^): m/z [M + H]^+^ calcd for C_25_H_27_N_3_O_4_S, 466.1801; found, 466.1817.

N-(4-((4-(3-fluorobenzoyl)piperazin-1-yl)methyl)phenyl)-4-methoxybenzenesulfonamide (**11**)

(84 mgs, 0.17 mmol, 80%); ^1^H NMR (500 MHz, DMSO-d_6_) δ 10.06 (br s, 1H), 7.69 – 7.67 (m, 2H), 7.51 – 7.46 (m, 1H), 7.31 – 7.27 (m, 1H), 7.22 – 7.19 (m, 2H), 7.16 – 7.14 (m, 2H), 7.05 – 7.03 (m, 4H), 3.79 (s, 3H), 3.60 – 3.58 (m, 2H), 3.39 (s, 2H), 2.34 – 2.30 (m, 4H). Note, two piperazine protons hidden under solvent peak.; MS (ES+): *m/z* (%) 484 (100) [M+H]^+^.

N-(4-((4-(5-chloro-2-methoxybenzoyl)piperazin-1-yl)methyl)phenyl)-4-methoxybenzenesulfonamide (**14**)

(46 mgs, 0.08 mmol, 40%); ^1^H NMR (500 MHz, DMSO-d_6_) δ 10.07 (br s, 1H), 7.69 – 7.65 (m, 2H), 7.44 – 7.40 (m, 1H), 7.22 – 7.19 (m, 1H), 7.14 (d, *J* = 8.4 Hz, 2H), 7.09 (d, *J* = 8.7 Hz, 1H), 7.06 – 7.01 (m, 4H), 3.78 (s, 3H), 3.76 (s, 3H), 3.59 – 3.53 (m, 2H), 3.40 – 3.36 (m, 2H), 3.12 – 3.06 (m, 2H), 2.36 – 2.30 (m, 2H), 2.26 – 2.20 (m, 2H) .; ^13^C NMR (126 MHz, DMSO-d_6_) δ 165.1, 162.8, 154.2, 137.2, 133.7, `131.7, 130.3, 130.0, 129.3, 127.8, 127.6, 124.8, 120.3, 114.8, 113.8, 61.6, 56.3, 56.0, 53.0, 52.5, 46.7, 41.5.; HRMS (ES^+^): m/z [M + H]^+^ calcd for C_26_H_28_N_3_O_5_SCl, 530.1516; found, 530.1533.

N-(4-((4-(3-chloro-4-methylbenzoyl)piperazin-1-yl)methyl)phenyl)-4-methoxybenzenesulfonamide (**15**)

(78 mgs, 0.14 mmol, 70%); ^1^H NMR (500 MHz, DMSO-d_6_) δ 10.07 (br s, 1H), 7.70 – 7.65 (m, 2H), 7.42 – 7.38 (m, 2H), 7.26 – 7.21 (m, 1H), 7.15 (d, *J* = 8.4 Hz, 2H), 7.07 – 7.00 (m, 4H), 3.78 (s, 3H), 3.66 – 3.44 (m, 2H), 3.38 (s, 2H), 2.38 – 2.23 (m, 4H), 2.35 (s, 3H) .; ^13^C NMR (126 MHz, DMSO-d_6_) δ 167.8, 162.8, 137.4, 137.2, 135.8, 133.7, 133.7, 131.7, 131.6, 130.0, 129.3, 127.7, 126.0, 120.3, 114.8, 61.6, 56.1, 52.9, 52.6, 47.6, 42.0, 19.9.; Note, two piperazine protons hidden under solvent peak.; HRMS (ES^+^): m/z [M + H]^+^ calcd for C_26_H_28_N_3_O_4_SCl, 514.1567; found, 514.1588.

N-(4-((4-(5-chloronicotinoyl)piperazin-1-yl)methyl)phenyl)-4-methoxybenzenesulfonamide (**16**)

(74 mg, 0.15 mmol, 29%); ^1^H NMR (500 MHz, DMSO-d_6_) δ 10.08 (br s, 1H), 8.71 – 8.69 (m, 1H), 8.55 – 8.53 (m, 1H), 8.00 – 7.97 (m, 1H), 7.69 – 7.65 (m, 2H), 7.15 (d, *J* = 8.4 Hz, 2H), 7.06 – 7.01 (m, 4H), 3.79 (s, 3H), 3.65-3.55 (m, 2H), 3.39 (s, 2H), 2.43-2.23 (m, 4H) .; ^13^C NMR (126 MHz, DMSO-d_6_) δ 165.4, 162.8, 149.4, 146.2, 137.2, 134.8, 133.7, 133.5, 131.7, 131.5, 130.1, 129.3, 120.3, 114.8, 61.5, 56.0, 52.9, 52.3, 47.5, 42.0. Note, two piperazine protons hidden under solvent peak.; HRMS (ES^+^): m/z [M + H]^+^ calcd for C_24_H_25_N_4_O_4_SCl, 501.1363; found, 501.1370.

4-methoxy-N-(4-((4-(5-(trifluoromethyl)-1H-pyrazole-3-carbonyl)piperazin-1-yl)methyl)phenyl)benzenesulfonamide (**17**)

(119 mg, 0.22 mmol, 41%); ^1^H NMR (400 MHz, CDCl_3_) δ 12.58 (br s, 1H), 7.76 – 7.72 (m, 2H), 7.22 – 7.20 (m, 2H), 7.07 – 7.04 (m, 3H), 6.93 – 6.89 (m, 2H), 6.71 (s, 1H), 3.85 (s, 3H), 3.83 – 3.81 (m, 4H), 3.50 (2H, s), 2.52 – 2.49 (m, 4H).; MS (ES+): *m/z* 524 [M+H]^+^.

4-methoxy-N-(4-((4-(2-(2-oxopyrimidin-1(2H)-yl)acetyl)piperazin-1-yl)methyl)phenyl)benzenesulfonamide (**18**)

(103 mg, 0.20 mmol, 29%); ^1^H NMR (500 MHz, DMSO-d_6_) δ 10.08 (br s, 1H), 8.57 – 8.54 (m, 1H), 8.05 – 8.01 (m, 1H), 7.71 – 7.66 (m, 2H), 7.17 (d, *J* = 8.4 Hz, 2H), 7.07 – 7.02 (m, 4H), 6.45 – 6.41 (m, 1H), 4.78 (s, 2H), 3.79 (s, 3H), 3.50– 3.41 (m, 4H), 3.40 (s, 2H), 2.40 – 2.35 (m, 2H), 2.30 – 2.26 (m, 2H) .; ^13^C NMR (126 MHz, DMSO-d_6_) δ 166.9, 165.0, 162.8, 156.1, 151.3, 137.2, 133.7, 131.7, 130.1, 129.3, 120.3, 114.8, 103.9, 61.6, 56.1, 52.9, 52.4, 51.2, 44.6, 42.1.; HRMS (ES^+^): m/z [M + H]^+^ calcd for C_24_H_27_N_5_O_5_S, 498.1811; found, 498.1825.

4-methoxy-N-(4-((4-(2-methylpyrimidine-4-carbonyl)piperazin-1-yl)methyl)phenyl)benzenesulfonamide (**19**)

(32 mg, 0.06 mmol, 13%); ^1^H NMR (500 MHz, DMSO-d_6_) δ 10.09 (br s, 1H), 8.80 (d, *J* = 5.0 Hz, 1H), 7.67 – 7.65 (m, 2H), 7.39 (d, *J* = 5.0 Hz, 1H), 7.15 – 7.13 (m, 2H), 7.05 – 7.01 (m, 4H), 3.77 (s, 3H), 3.60 – 3.57 (m, 2H), 3.38 (s, 2H), 3.29 – 3.25 (m, 2H), 2.62 (s, 3H), 2.37 – 2.35 (m, 2H), 2.28 – 2.25 (m, 2H).; MS (ES+): *m/z* (%) 482 [M+H]^+^.

4-methoxy-N-(4-((4-(1-methylpiperidine-3-carbonyl)piperazin-1-yl)methyl)phenyl)benzenesulfonamide (**20**)

(75 mg, 0.15 mmol, 27%); ^1^H NMR (500 MHz, DMSO-d_6_) δ 10.70 – 9.48 (br s, 1H), 7.70 – 7.65 (m, 2H), 7.14 (d, *J* = 8.4 Hz, 2H), 7.07 – 7.01 (m, 4H), 3.79 (s, 3H), 2.81 – 2.68 (m, 2H), 2.34 – 2.13 (m, 7H), 2.05 – 1.93 (m, 1H), 1.93 – 1.79 (m, 1H), 1.69 – 1.58 (m, 2H), 1.58 – 1.46 (m, 1H), 1.31 – 1.19 (m, 1H) .; ^13^C NMR (126 MHz, DMSO-d_6_) δ 171.8, 162.8, 137.2, 133.8, 131.7, 130.0, 129.3, 120.3, 114.8, 61.6, 58.0, 56.0, 55.5, 53.5, 52.7, 46.4, 45.3, 41.4, 38.3, 27.0, 24.5. Note, three protons hidden under solvent peak.; HRMS (ES^+^): m/z [M + H]^+^ calcd for C_25_H_34_N_4_O_4_S , 487.2379; found, 487.2303.

N-(4-((4-(1-acetylazetidine-3-carbonyl)piperazin-1-yl)methyl)phenyl)-4-methoxybenzenesulfonamide (**21**)

(50 mg, 0.10 mmol, 19%); ^1^H NMR (500 MHz, DMSO-d_6_) δ 10.09 (br s, 1H), 7.70 – 7.66 (m, 2H), 7.14 (d, *J* = 8.4 Hz, 2H), 7.07 – 7.02 (m, 4H), 4.25 – 4.14 (m, 2H), 3.99 – 3.92 (m, 1H), 3.88 – 3.82 (m, 1H), 3.79 (2, 3H), 3.66 – 3.59 (m, 1H), 3.47 – 3.41 (m, 2H), 3.36 (s, 2H), 3.24 – 3.20 (m, 2H), 2.29 – 2.23 (m, 4H), 1.73 (s, 3H) .; ^13^C NMR (126 MHz, DMSO-d_6_) δ 170.1, 169.6, 162.8, 137.2, 133.8, 131.7, 130.0, 129.3, 120.3, 114.8, 61.6, 56.1, 53.0, 52.5, 52.3, 50.0, 44.9, 41.7, 30.0, 18.9.; HRMS (ES^+^): m/z [M + H]^+^ calcd for C_24_H_30_N_4_O_5_S, 487.2015; found, 487.2028.

*N*-(4-((4-(3-hydroxy-3-(trifluoromethyl)cyclobutane-1-carbonyl)piperazin-1-yl)methyl)phenyl)-4-methoxybenzenesulfonamide (**22**)

(46 mg, 0.08 mmol, 34%); ^1^H NMR (500 MHz, DMSO-d_6_) δ 10.07 (br s, 1H), 7.68 (d, *J* = 7.7 Hz, 2H), 7.15 – 7.13 (m, 2H), 7.06 – 7.02 (m, 4H), 6.51 (s, 1H), 3.80 (s, 3H), 3.44 – 3.40 (m, 2H), 3.36 (s, 2H), 3.29 – 3.26 (m, 2H), 2.98 – 2.91 (m, 1H), 2.58 – 2.53 (m, 2H), 2.39 – 2.31 (m, 2H), 2.26 – 2.23 (m, 4H); ^13^C NMR (126 MHz, DMSO-d_6_) δ 171.1, 162.8, 137.2, 133.8, 131.8, 130.1, 129.3, 120.3, 114.8, 69.3 – 68.8 (q, *J* = 31.3 Hz, C-F3), 61.7, 56.1, 53.2, 52.7, 44.9, 41.8, 34.2, 27.3; HRMS (ES^+^): m/z [M + H]^+^ calcd for C_24_H_29_F_3_N_3_O_5_S, 528.1780; found, 528.1770.

*N*-(4-((4-(1*H*-indole-2-carbonyl)piperazin-1-yl)methyl)phenyl)-4-methylbenzenesulfonamide (**23**)

(22 mgs, 0.04 mmol, 21%); ^1^H NMR (500 MHz, DMSO-d_6_) δ 11.52 (s, 1H), 10.15 (br s, 1H), 7.63 (d, *J* = 8.3 Hz, 2H), 7.58 (d, *J* = 8.0 Hz, 1H), 7.41 (d, *J* = 8.2 Hz, 1H), 7.33 (d, *J* = 8.1 Hz, 1H), 7.19 – 7.16 (m, 3H), 7.06 – 7.02 (m, 3H), 6.75 (d, *J* = 1.5 Hz, 1H), 3.76 – 3.70 (m, 4H), 3.41 (s, 2H), 2.38 – 2.36 (m, 4H), 2.33 (s, 3H); ^13^C NMR (126 MHz, DMSO-d_6_) δ 162.4, 143.6, 137.3, 137.2, 136.4, 133.9. 130.4, 130.1, 130.0, 127.3, 127.1, 123.6, 121.8, 120.4, 120.2, 112.5, 104.4, 61.7, 53.1, 21.4; m/z [M + H]^+^ calcd for C_27_H_29_N_4_O_3_S, 489.1960; found, 489.1971.

*N-*(4-((4-(2,3-dihydro-1*H*-indene-1-carbonyl)piperazin-1-yl)methyl)phenyl)-4-methylbenzenesulfonamide (**24**)

(38 mgs, 0.07 mmol, 36%); ^1^H NMR (500 MHz, DMSO-d_6_) δ 10.15 (s, 1H), 7.63 (d, *J* = 8.3 Hz, 2H), 7.34 (d, *J* = 8.0 Hz, 2H), 7.22 (d, *J* = 7.0 Hz, 1H), 7.19 – 7.11 (m, 4H), 7.09 – 7.04 (m, 3H), 4.41 – 4.38 (m, 1H), 3.69 – 3.65 (m, 2H), 3.55 – 3.46 (m, 2H), 3.41 (s, 2H), 3.00 – 2.94 (m, 1H), 2.87 – 2.80 (m, 1H), 2.45 – 2.28 (m, 7H), 2.25 – 2.15 (m, 2H); ^13^C NMR (126 MHz, DMSO-d_6_) δ 171.9. 144.6, 143.6, 143.1, 137.3, 137.1, 134.0, 130.1, 130.1, 127.2, 127.1, 126.5, 124.8, 124.7, 120.4, 61.6, 53.6, 52.9, 46.5, 45.9, 41.9, 31.9, 30.1, 21.4; m/z [M + H]^+^ calcd for C_28_H_32_N_3_O_3_S, 490.2197; found, 490.2164 (6.7 ppm).

*N*-(4-((4-(benzofuran-3-carbonyl)piperazin-1-yl)methyl)phenyl)-4-methylbenzenesulfonamide (**25**)

(28 mgs, 0.05 mmol, 26%); ^1^H NMR (500 MHz, DMSO-d_6_) δ 10.14 (s, 1H), 7.83 (s, 1H), 7.63 (d, *J* = 8.2 Hz, 2H), 7.60 (d, *J* = 7.7 Hz, 1H), 7.54 (d, *J* = 8.2 Hz, 1H), 7.33 (d, *J* = 8.2 Hz, 2H), 7.30 (d, *J* = 8.2 Hz, 1H), 7.25 – 7.21 (m, 1H), 7.14 (d, *J* = 8.4 Hz, 2H), 7.03 (d, *J* = 8.4 Hz, 2H), 3.77 (s, 2H), 3.55 – 3.43 (m, 4H), 3.34 (s, 2H), 2.33 (s, 3H), 2.27 – 2.22 (m, 4H); ^13^C NMR (126 MHz, DMSO-d_6_) δ 168.1, 154.9, 143.6, 143.5, 137.3, 137.1, 133.9, 130.1, 130.1, 128.4, 127.1, 124.7, 122.9, 120.8, 120.4, 115.2, 111.6, 61.6, 53.1, 52.6, 45.9, 41.7, 28.8, 21.4; m/z [M + H]^+^ calcd for C_28_H_30_N_3_O_4_S, 504.1957; found, 504.1967.

*N*-(4-((4-(but-2-ynoyl)piperazin-1-yl)methyl)phenyl)-4-methylbenzenesulfonamide (**26**)

(31 mgs, 0.07 mmol, 35%); ^1^H NMR (500 MHz, DMSO-d_6_) δ 10.15 (br s, 1H), 7.63 (d, *J* = 8.3 Hz, 2H), 7.32 (d, *J* = 8.1 Hz, 2H), 7.14 (d, *J* = 8.4 Hz, 2H), 7.03 (d, *J* = 8.5 Hz, 2H), 3.64 – 3.42 (m, 4H), 3.37 (s, 2H), 2.35 – 2.31 (m, 5H), 2.24 – 2.22 (m, 2H), 2.00 (s, 3H); ^13^C NMR (126 MHz, DMSO-d_6_) δ 152.3, 143.6, 137.3, 137.2, 133.7, 130.1, 130.0, 127.1, 120.4, 89.8, 73.4, 61.5, 53.1, 52.2, 46.7, 42.1, 21.4, 3.8; m/z [M + H]^+^ calcd for C_22_H_26_N_3_O_3_S, 412.1695; found, 412.1690.

4-methoxy-N-(4-((4-(1-methyl-4-oxo-1,4-dihydroquinoline-2-carbonyl)piperazin-1-yl)methyl)phenyl)benzenesulfonamide (**27**)

(5 mgs, 0.01 mmol, 5%); ^1^H NMR (500 MHz, DMSO-d_6_) δ 10.13 (s, 1H), 8.17 – 8.16 (m, 1H), 7.96 – 7.94 (m, 1H), 7.81 – 7.78 (m, 1H), 7.63 – 7.61 (m, 3H), 7.33 – 7.32 (m, 2H), 7.16 (d, *J* = 8.4 Hz, 2H), 7.12 (s, 1H), 7.03 (d, *J* = 8.4 Hz, 2H), 4.08 (s, 3H), 3.69 – 3.66 (m, 2H), 3.42 – 3.40 (m, 4H), 2.43 – 2.41 (m, 2H), 3.33 – 3.32 (m, 5H).; MS (ES+): *m/z* (%) 531 (100) [M+H]^+^.

*N*-(4-((1-(3-chlorobenzoyl)piperidin-4-yl)oxy)phenyl)-4-methoxybenzenesulfonamide (**28**)

(90 mg, 0.18 mmol, 46%); ^1^H NMR (500 MHz, DMSO-d_6_) δ 9.77 (br s, 1H), 7.61 (d, *J* = 8.9 Hz, 2H), 7.53 – 7.45 (m, 3H), 7.38 – 7.35 (m, 1H), 7.04 (d, *J* = 8.9 Hz, 2H), 6.96 (d, *J* = 8.9 Hz, 2H), 6.85 (d, *J* = 8.9 Hz, 2H), 4.55 – 4.49 (m, 1H), 3.98 – 3.88 (m, 1H), 3.79 (s, 3H), 3.50 – 3.38 (m, 2H), 3.29 – 3.18 (m, 1H), 1.99 – 1.52 (m, 4H); ^13^C NMR (126 MHz, DMSO-d_6_) δ 167.8, 162.7, 154.4, 138.8, 133.7, 131.8, 131.3, 130.9, 129.8, 129.3, 127.0, 125.7, 123.6, 116.9, 114.7, 72.5, 56.1, 44.6, 39.1, 31.1, 30.5. Note, piperazine carbon peaks are weak and broad; m/z [M + H]^+^ calcd for C_25_H_26_ClN_2_O_5_S, 501.1251; found, 501.1255.

*N*-(4-(5-(3-chlorobenzoyl)-5,6-dihydropyrrolo[3,4-*c*]pyrazol-2(4*H*)-yl)phenyl)-4-methoxybenzenesulfonamide (**29**)

(17 mg, 0.03 mmol, 29%); ^1^H NMR (500 MHz, DMSO-d_6_) δ 10.18 (br s, 1H), 7.70 – 7.47 (m, 7H), 7.13 – 7.11 (m, 2H), 7.06 – 7.03 (m, 4H), 5.18 (s, 2H), 4.58 – 4.43 (m, 4H), 3.79 (s, 3H); ^13^C NMR (126 MHz, DMSO-d_6_) 168.3, 168.2, 162.9, 153.9, 152.9, 139.3, 139.2, 137.8, 133.6, 133.5, 131.7, 130.9, 130.8, 130.2, 130.1, 129.3, 129.0, 129.0, 127.2, 127.1, 125.9, 124.6, 124.5, 120.2, 119.0, 117.8, 114.9, 56.1, 55.0, 48.4, 48.1, 45.9, 45.8. Note, some of the peaks are doubled due to restricted rotation.; m/z [M + H]^+^ calcd for C_26_H_24_ClN_4_O_4_S, 523.1207; found, 523.1202.

(*S*)-3-chloro-*N*-(1-(4-((4-methoxyphenyl)sulfonamido)benzyl)pyrrolidin-3-yl)benzamide (**30**)

(64 mg, 0.12 mmol, 47%); ^1^H NMR (500 MHz, DMSO-d_6_) δ 10.05 (br s, 1H), 8.55 (d, *J* = 8.5 Hz, 1H), 7.90 – 7.89 (m, 1H), 7.80 (d, *J* = 7.8 Hz, 1H), 7.67 (d, *J* = 8.9 Hz, 2H), 7.59 – 7.57 (m, 1H), 7.50 – 7.47 (m, 1H), 7.16 (d, *J* = 8.4 Hz, 2H), 7.05 – 7.02 (m, 4H), 4.38 – 4.30 (m, 1H), 3.78 (s, 3H), 3.47 (s, 2H), 2.75 – 2.70 (m, 1H), 2.57 – 2.52 (m, 1H), 2.47 – 2.41 (m, 1H), 2.36 – 2.33 (m, 1H), 2.16 – 2.08 (m, 1H), 1.79 – 1.73 (m, 1H); ^13^C NMR (126 MHz, DMSO-d_6_) δ 165.1, 162.8, 137.0, 136.8, 135.1, 133.5, 131.8, 131.4, 130.6, 129.8, 129.3, 127.6, 126.6, 120.4, 114.8, 60.1, 59.7, 59.2, 56.1, 52.9, 49.3, 31.1; m/z [M + H]^+^ calcd for C_25_H_27_ClN_3_O_4_S, 500.1411; found, 500.1417.

(*S*)-N-(4-(((1-(3-chlorobenzoyl)pyrrolidin-3-yl)amino)methyl)phenyl)-4-methylbenzenesulfonamide (**31**)

(113 mg, 0.23 mmol, 53%); ^1^H NMR (500 MHz, DMSO-d_6_) δ 10.07 (br s, 1H), 7.62 (dd, *J* = 8.2 and 8.2 Hz, 2H), 7.54 – 7.49 (m, 2H), 7.47 – 7.41 (m, 2H), 7.34 – 7.31 (m, 2H), 7.22 – 7.19 (m, 1H), 7.13 – 7.11 (m, 1H), 7.05 – 6.97 (m, 2H), 3.61 – 3.40 (m, 5H), 3.31 – 3.27 (m, 1H, under water peak), 3.26 – 3.21 (m, 1H), 3.18 – 3.11 (m, 1H), 2.32 (s, 3H), 1.98 – 1.84 (m, 1H), 1.77 – 1.67 (m, 1H); ^13^C NMR (126 MHz, DMSO-d_6_) δ 167.2, 167.2, 143.5, 139.6, 139.5, 137.3, 137.1, 137.0, 136.7, 136.6, 133.5, 130.7, 130.1, 130.0, 129.2, 129.0, 127.3, 127.2, 126.1, 120.5, 120.4, 57.4, 55.7, 54.8, 52.2, 51.0, 50.9, 47.6, 44.9, 32.1, 30.2, 21.4. Note, some of the peaks are doubled due to restricted rotation.; m/z [M + H]^+^ calcd for C_25_H_27_ClN_3_O_3_S, 484.1462; found, 484.1488 (5.4 ppm).

(*R*)-*N*-(4-(((1-(3-chlorobenzoyl)pyrrolidin-3-yl)amino)methyl)phenyl)-4-methylbenzenesulfonamide (**32**)

(305 mg, 0.63 mmol, 58%); ^1^H NMR (500 MHz, DMSO-d_6_) δ 10.02 (br s, 1H), 7.62 (dd, *J* = 8.2, 8.2 Hz, 2H), 7.54 – 7.49 (m, 2H), 7.47 – 7.41 (m, 2H), 7.34 – 7.31 (m, 2H), 7.20 (d, *J* = 8.3 Hz, 1H), 7.12 (d, *J* = 8.4 Hz, 1H), 7.04 – 6.98 (m, 2H), 3.61 (s, 1H), 3.59 – 3.40 (m, 4H), 3.26 – 3.21 (m, 1H), 3.18 – 3.12 (m, 1H), 2.33 (br s, 4H), 1.97 – 1.85 (m, 1H), 1.77 – 1.67 (m, 1H); ^13^C NMR (126 MHz, DMSO-d_6_) δ 167.2, 167.2, 143.6, 139.6, 139.5, 137.3, 137.1, 137.1, 136.7, 136.6, 133.5, 130.7, 130.1, 130.0, 129.2, 129.0, 127.3, 127.2, 126.1, 120.5, 120.4, 57.4, 55.7, 54.8, 52.2, 51.0, 50.9, 47.6, 44.9, 32.1, 30.1, 21.4. Note, some of the peaks are doubled due to restricted rotation.; m/z [M + H]^+^ calcd for C_25_H_27_ClN_3_O_3_S, 484.1462; found, 484.1479.

(*S*)-*N*-(4-(((1-(3-chlorobenzoyl)pyrrolidin-3-yl)(methyl)amino)methyl)phenyl)-4-methylbenzenesulfonamide (**33**)

(85 mg, 0.17 mmol, 58%); ^1^H NMR (500 MHz, DMSO-d_6_) δ 10.11 (br s, 1H), 7.62 (dd, *J* = 7.7, 7.7 Hz, 2H), 7.54 – 7.51 (m, 2H), 7.47 – 7.44 (m, 2H), 7.35 – 7.31 (m, 2H), 7.18 – 7.08 (m, 2H), 7.06 – 6.99 (m, 2H), 3.74 – 3.56 (m, 5H), 3.07 – 2.96 (m, 1H), 2.33 (s, 3H), 2.13 – 1.93 (m, 4H), 1.86 – 1.73 (m, 1H). Note, one proton hidden under solvent peak.; ^13^C NMR (126 MHz, DMSO-d_6_) δ 167.2, 143.6, 139.3, 139.1, 137.3, 137.0, 135.1, 133.5, 130.7, 130.2, 130.1, 129.9, 129.8, 127.4, 127.3, 127.1, 126.1, 126.1, 120.5, 120.4, 63.9, 62.4, 59.2, 59.1, 52.6, 50.0, 48.2, 45.3, 30.1, 28.0, 21.4. Note, some of the peaks are doubled due to restricted rotation.; m/z [M + H]^+^ calcd for C_26_H_29_ClN_3_O_3_S, 498.1618; found, 498.1627.

(*S*)-*N*-(4-(((1-(3-chlorobenzoyl)pyrrolidin-3-yl)(methyl)amino)methyl)phenyl)-4-methylbenzenesulfonamide (**34**)

(435 mg, 0.87 mmol, 69%); ^1^H NMR (500 MHz, DMSO-d_6_) δ 10.11 (br s, 1H), 7.62 (dd, *J* = 7.7, 7.7 Hz, 2H), 7.54 – 7.51 (m, 2H), 7.47 – 7.44 (m, 2H), 7.35 – 7.31 (m, 2H), 7.17 – 7.08 (m, 2H), 7.05 – 6.99 (m, 2H), 3.75 – 3.37 (m, 5H), 3.08 – 2.96 (m, 1H), 2.33 (s, 3H), 2.14 – 1.93 (m, 4H), 1.86 – 1.73 (m, 1H). Note, one proton hidden under solvent peak.; ^13^C NMR (126 MHz, DMSO-d_6_) δ 167.2, 143.6, 139.3, 139.1, 137.3, 137.0, 136.9, 135.1, 133.5, 130.7, 130.2, 130.1, 129.9, 129.8, 127.4, 127.3, 127.1, 126.1, 126.0, 120.5, 120.4, 63.9, 62.4, 59.2, 59.2, 52.6, 50.0, 48.2, 45.3, 30.1, 28.0, 21.4. Note, some of the peaks are doubled due to restricted rotation.; m/z [M + H]^+^ calcd for C_26_H_29_ClN_3_O_3_S, 498.1618; found, 498.1632.

N-(4-(((3aR,6aS)-5-(3-chlorobenzoyl)hexahydropyrrolo[3,4-c]pyrrol-2(1H)-yl)methyl)phenyl)-4-methylbenzenesulfonamide (**35**)

(90 mg, 0.18 mmol, 21%); ^1^H-NMR (500 MHz, DMSO-d_6_) δ 10.19 (br s, 1H), 7.65 (d, *J* = 7.6 Hz, 2H), 7.54 – 7.45 (m, 3H), 7.42 (d, *J*=7.4, 1H), 7.34 (d, *J* = 8.0 Hz, 2H), 7.30 – 7.02 (m, 4H), 4.45 – 2.12 (m, 15H). ^13^C NMR (126 MHz, DMSO-d_6_) δ 166.76, 158.39, 158.14, 143.82, 139.31, 137.26, 133.51, 132.57, 130.77, 130.17, 127.34, 127.16, 126.18, 120.15, 57.71, 21.40. Note, aliphatic peaks are weak and broad.; HRMS (ES^+^): m/z [M + H]^+^ calcd for C_27_H_29_N_3_O_3_SCl, 510.1618; found, 510.1625.

N-(4-((1-(3-chlorobenzoyl)piperidin-4-yl)amino)phenyl)-4-methylbenzenesulfonamide (**36**)

(40 mg, 0.08 mmol, 50%); ^1^H-NMR (500 MHz, DMSO-d_6_) δ 9.42 (s, 1H), 7.53 (m, 3H), 7.48 (t, *J* = 7.8 Hz, 1H), 7.42 (s, 1H), 7.32 (m, 3H), 6.74 (d, *J* = 8.9 Hz, 2H), 6.45 (d, *J* = 8.8 Hz, 2H), 5.42 (d, *J* = 8.0 Hz, 1H), 4.29 (br s, 1H), 3.49 (br s, 1H), 3.41 (m, 1H), 3.15 (br s, 1H), 3.03 (m, 1H), 2.34 (s, 3H), 1.92 (br s, 1H), 1.82 (br s, 1H), 1.28 (br s, 2H).; HRMS (ES^+^): m/z [M + H]^+^ calcd for C_25_H_26_N_3_O_3_SCl, 484.1462; found, 484.1484.

N-(4-(((1-(3-chlorobenzoyl)azetidin-3-yl)amino)methyl)phenyl)-4-methylbenzenesulfonamide (**38**)

(40 mg, 0.08 mmol, 17%); ^1^H-NMR (500 MHz, DMSO-d_6_) δ 10.07 (br s, 1H), 7.62 – 7.53 (m, 5H), 7.48 (t, *J* = 7.8 Hz, 1H), 7.32 (d, *J* = 8.0 Hz, 2H), 7.16 (d, *J* = 8.4 Hz, 2H), 7.01 (d, *J* = 8.4 Hz, 2H), 4.34 (t, *J* = 7.8 Hz, 1H), 4.06 (t, *J* = 8.7 Hz, 1H), 3.93 (m, 1H), 3.65 (m, 1H), 3.55 – 3.48 (m, 3H), 2.80 (br s, 1H), 2.33 (s, 3H). ;^13^C NMR (126 MHz, DMSO-d_6_) δ 167.80, 143.57, 137.21, 136.80, 136.72, 136.00, 133.67, 131.11, 130.85, 130.08, 129.29, 127.84, 127.14, 126.67, 120.60, 60.58, 56.48, 50.26, 47.99, 21.40.; HRMS (ES^+^): m/z [M + H]^+^ calcd for C_24_H_25_N_3_O_3_SCl, 470.1305; found, 470.1323.

N-(5-((4-(3-chlorobenzoyl)piperazin-1-yl)methyl)pyrimidin-2-yl)-4-methoxybenzenesulfonamide (**42**)

(30 mg, 0.06 mmol, 2%); ^1^H-NMR (500 MHz, DMSO-d_6_) δ 11.83 (br s, 1H), 8.56 (s, 2H), 7.84 (d, *J* = 8.9 Hz, 2H), 7.56 (d, *J* = 8.1 Hz, 1H), 7.50 (m, 2H), 7.39 (d, *J* = 7.5 Hz, 1H), 7.11 (d, *J* = 8.9 Hz, 2H), 4.24 – 2.90 (m, 13H). ^13^C NMR (126 MHz, DMSO-d_6_) δ 167.92, 163.15, 158.62, 158.36, 157.59, 137.54, 133.74, 132.17, 131.04, 130.48, 130.31, 127.33, 126.16, 114.51, 56.18, 54.60, 51.00. Note, piperazine carbon peaks are weak and broad.; HRMS (ES^+^): m/z [M + H]^+^ calcd for C_23_H_25_N_5_O_4_SCl, 502.1316; found, 502.1332.

N-(5-((4-(3-chlorobenzoyl)piperazin-1-yl)methyl)pyrazin-2-yl)-4-methoxybenzenesulfonamide (**45**)

(40 mg, 0.08 mmol, 11%); ^1^H-NMR (500 MHz, DMSO-d_6_) δ 11.26 (br s, 1H), 8.28 (s, 1H), 8.23 (s, 1H), 7.87 (d, *J* = 8.9 Hz, 2H), 7.52 (m, 1H), 7.46 (t, *J* = 7.8 Hz, 1H), 7.43 (s, 1H), 7.33 (d, *J* = 7.5 Hz, 1H), 7.09 (d, *J* = 8.9 Hz, 2H), 3.81 (s, 3H), 3.63 – 3.54 (m, 4H), 2.49 – 2.34 (m, 4H). Note, two protons hidden under solvent peak.; ^13^C NMR (126 MHz, DMSO-d_6_) δ 167.75, 163.13, 147.58, 147.24, 142.34, 138.46, 134.04, 133.66, 132.15, 130.89, 129.88, 127.13, 125.92, 114.82, 60.61, 56.16, 53.01, 52.53, 47.49, 42.03. Note, piperazine carbon peaks are weak and broad.; HRMS (ES^+^): m/z [M + H]^+^ calcd for C_23_H_25_N_5_O_4_SCl, 502.1316; found, 502.1335.

N-(5-((4-(3-chlorobenzoyl)piperazin-1-yl)methyl)-3-methoxypyrazin-2-yl)-4-methoxybenzenesulfonamide (**46**)

(11 mg, 0.02 mmol, 30%); ^1^H NMR (500 MHz, DMSO-d_6_) δ 10.63 (br s, 1H), 7.93 – 7.91 (m, 2H), 7.72 – 7.71 (m, 1H), 7.53 – 7.50 (m, 1H), 7.48 – 7.44 (m, 1H), 7.43 – 7.42 (m, 1H), 7.09 – 7.07 (m, 2H), 3.89 (s, 3H), 3.82 (s, 3H), 3.61 – 3.59 (m, 2H), 3.49 (s, 2H), 2.45 – 2.41 (m, 4H). Note, two piperazine protons hidden under solvent peak.; MS (ES+): *m/z* 532 [M+H]^+^.

**6. ^1^H, ^13^C NMR spectra and HRMS trace of final compounds**

**^1^H, ^13^C NMR and HRMS spectra of compound 1**

**
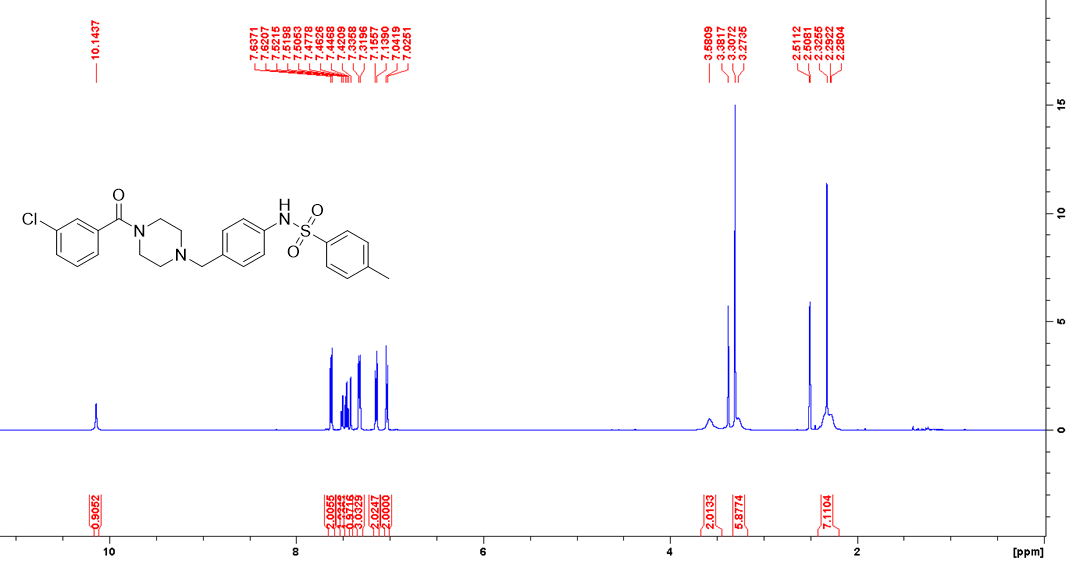
**

**
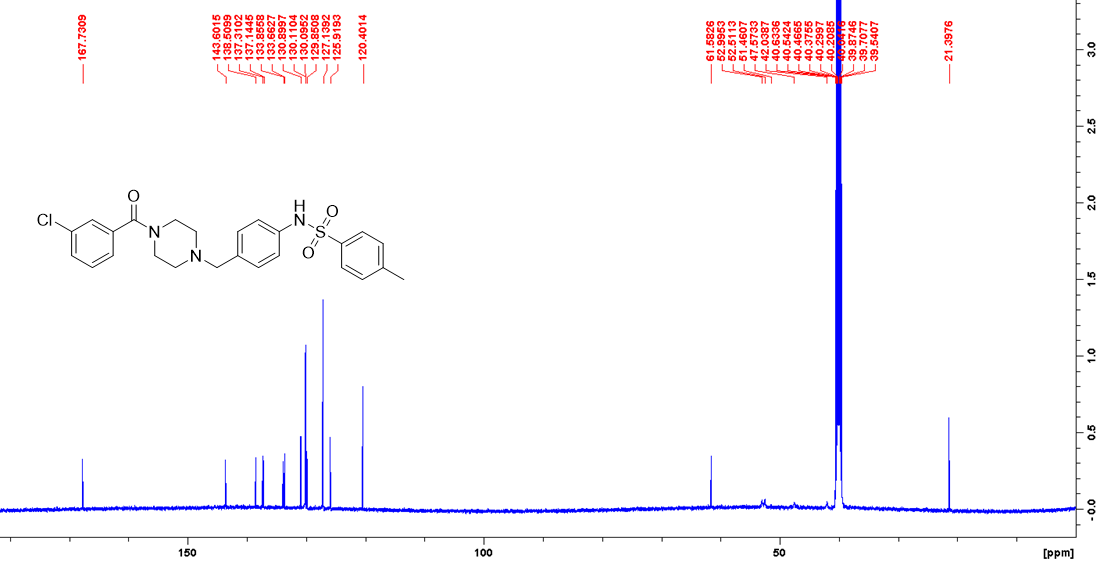
**

**
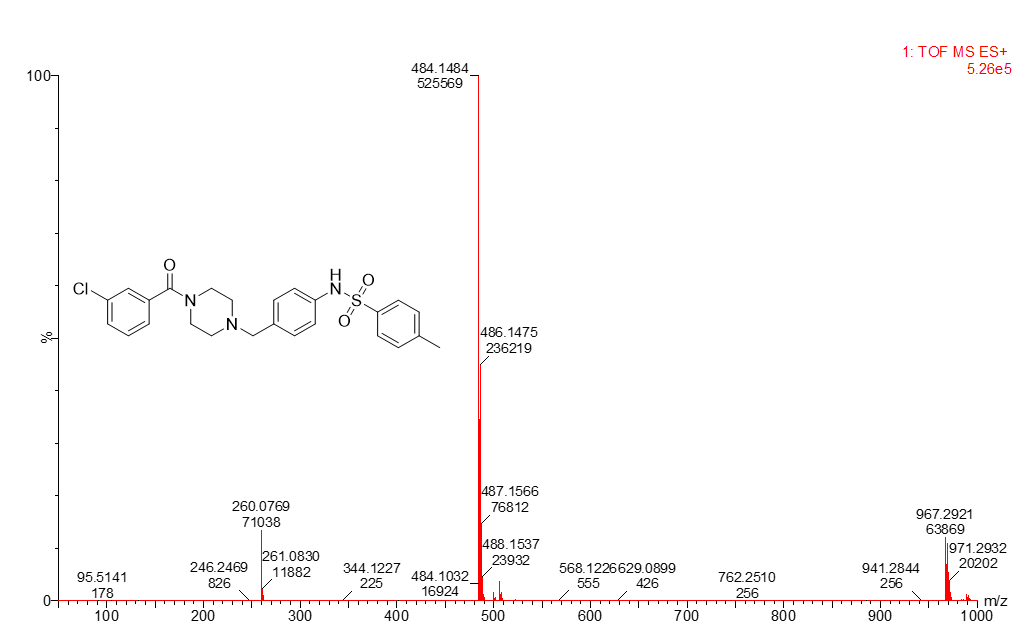
**

**^1^H, ^13^C NMR and HRMS spectra of compound 2**

**
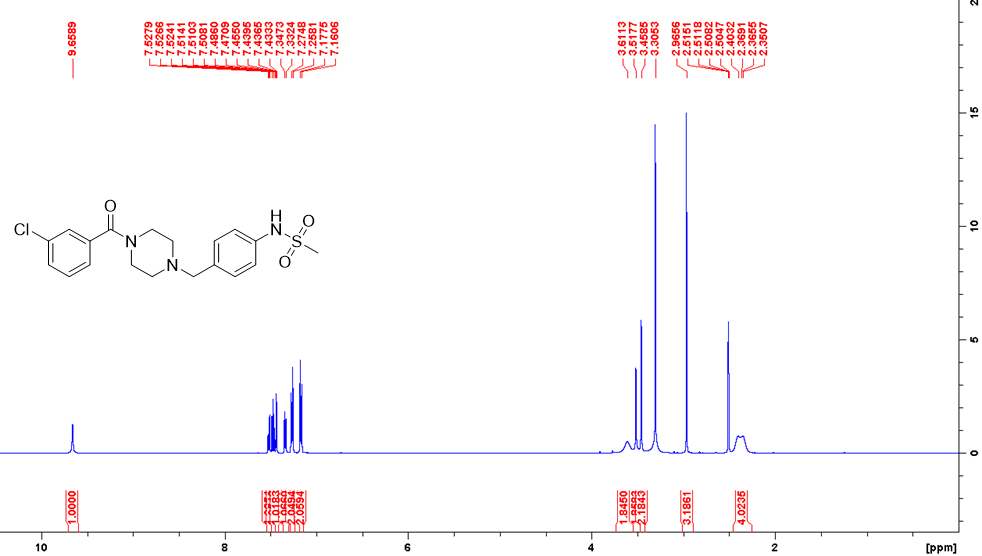
**

**
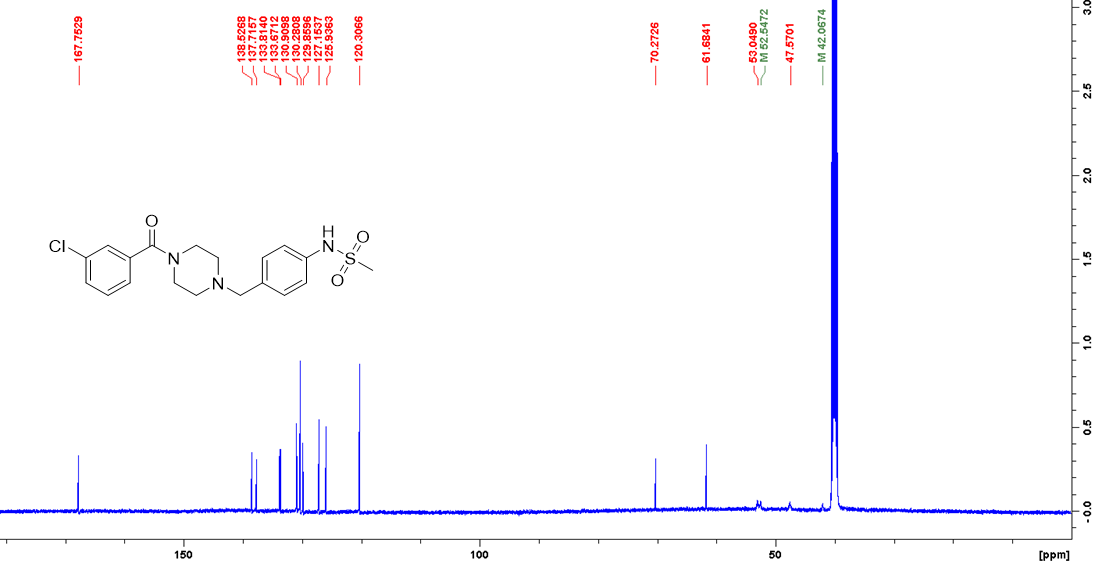
**

**
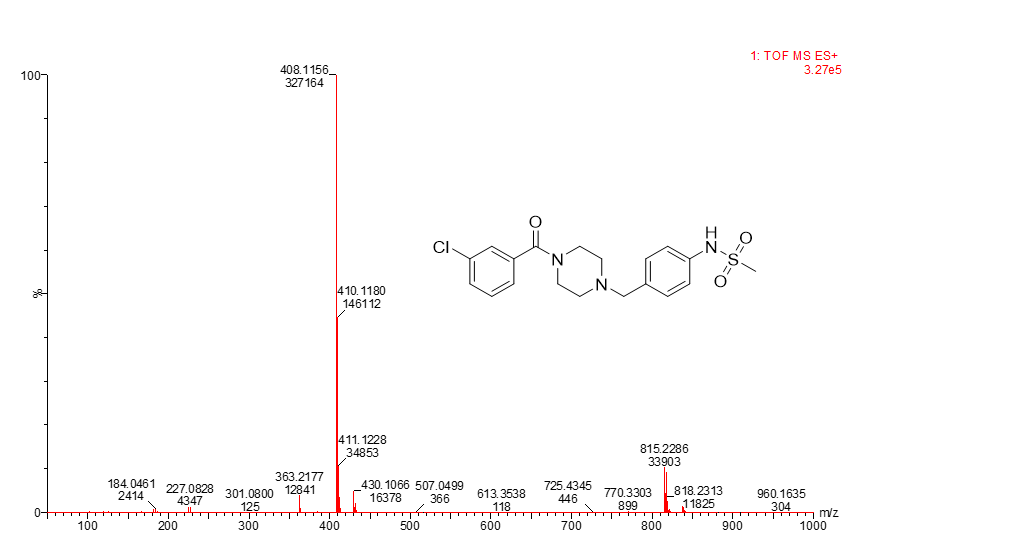
**

**^1^H, ^13^C NMR and HRMS spectra of compound 3**

**
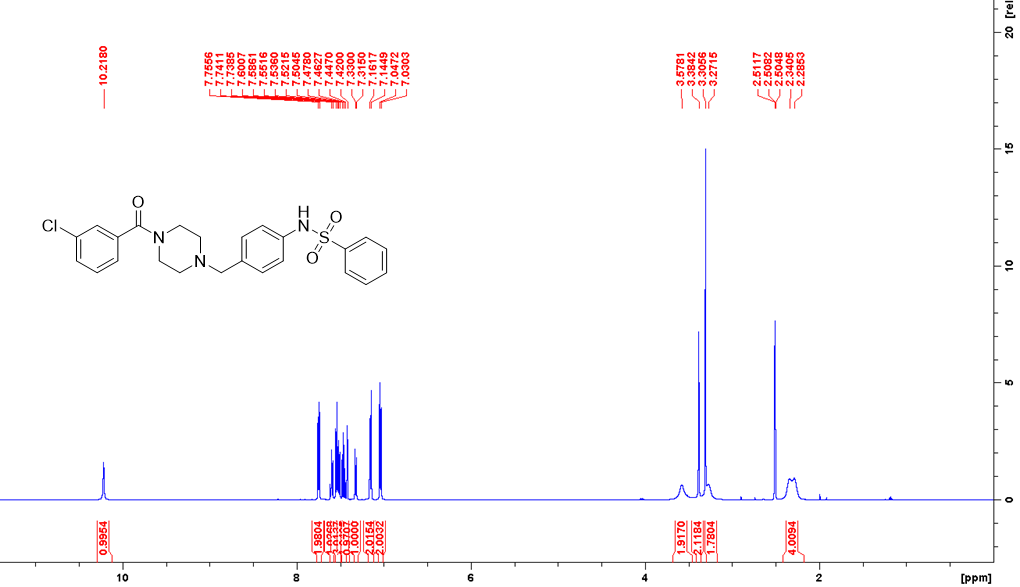
**

**
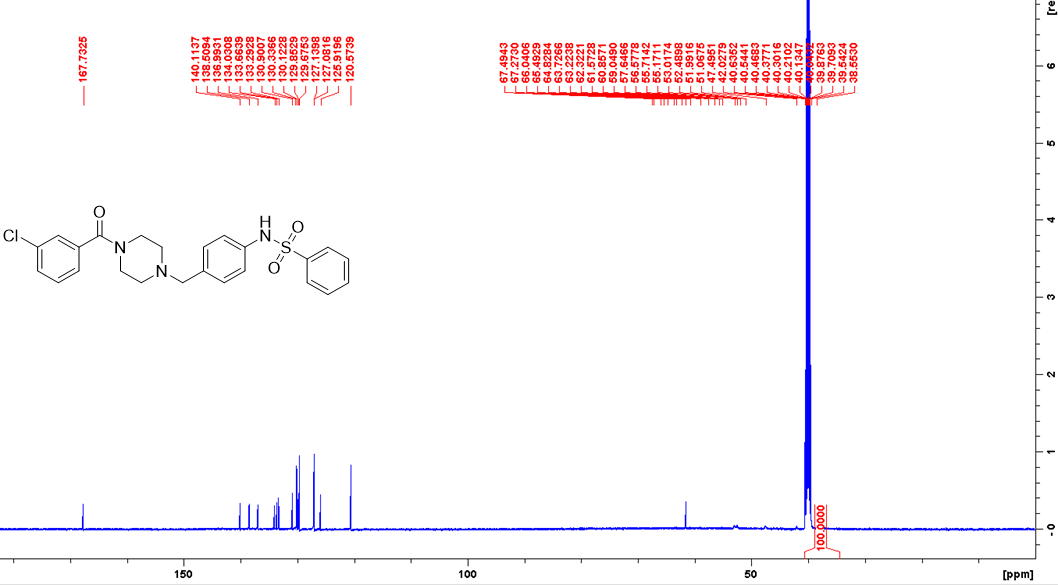
**

**
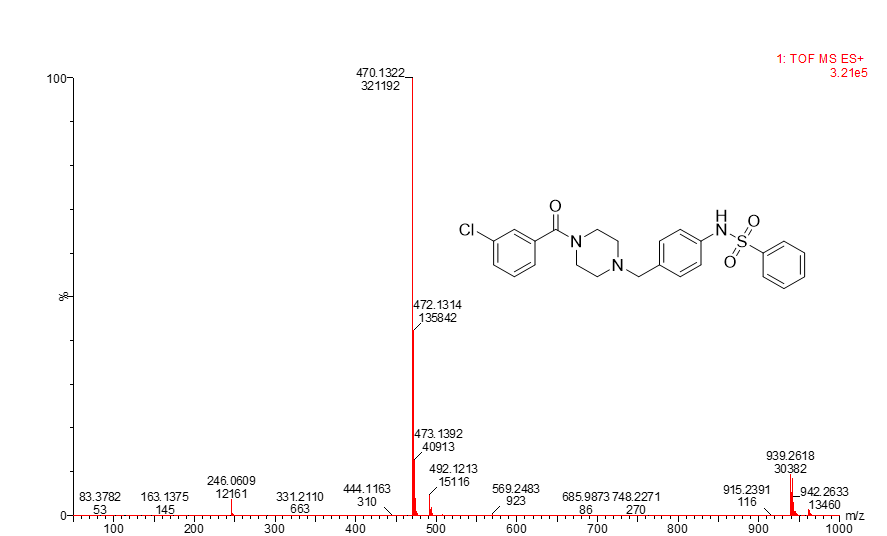
**

**^1^H, ^13^C NMR and HRMS spectra of compound 4**

**
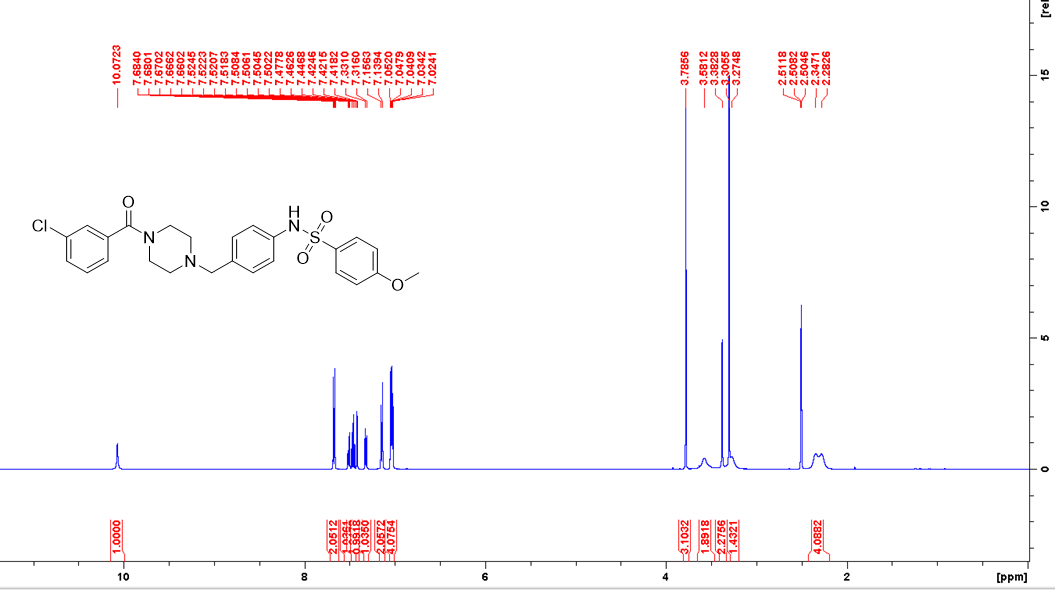
**

**
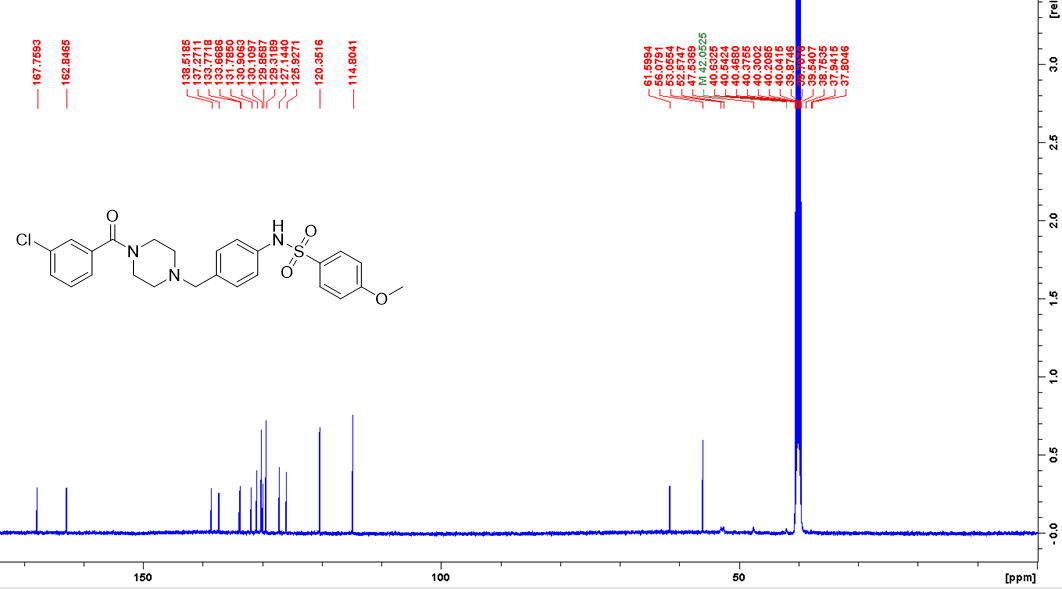
**

**
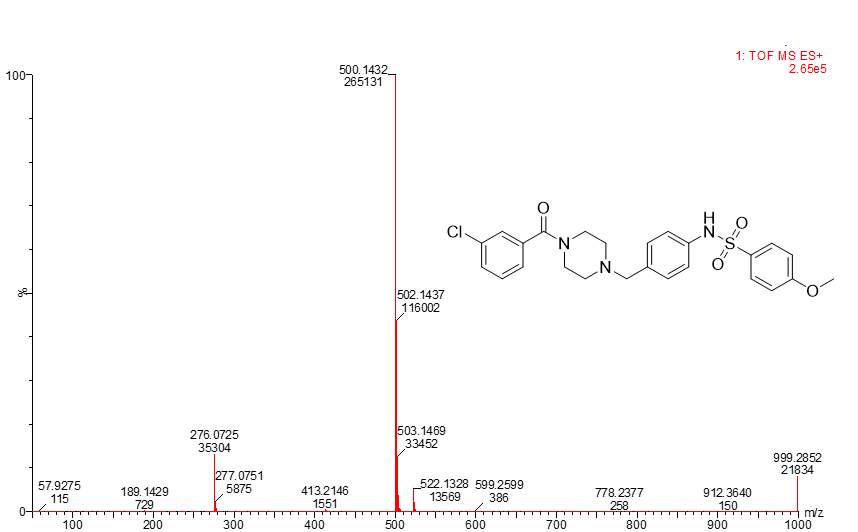
**

**^1^H NMR and HRMS spectra of compound 5**

**
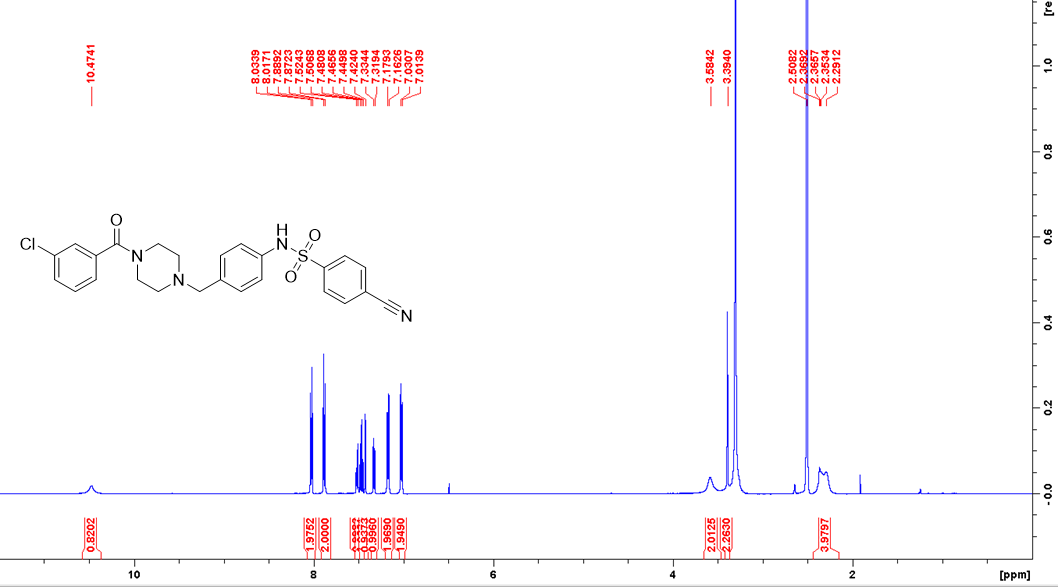
**


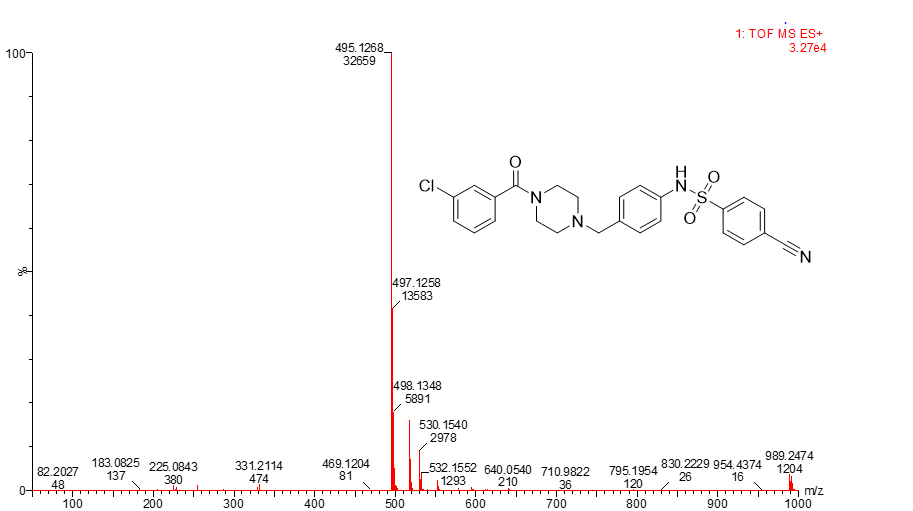


**^1^H, ^13^C NMR and HRMS spectra of compound 6**


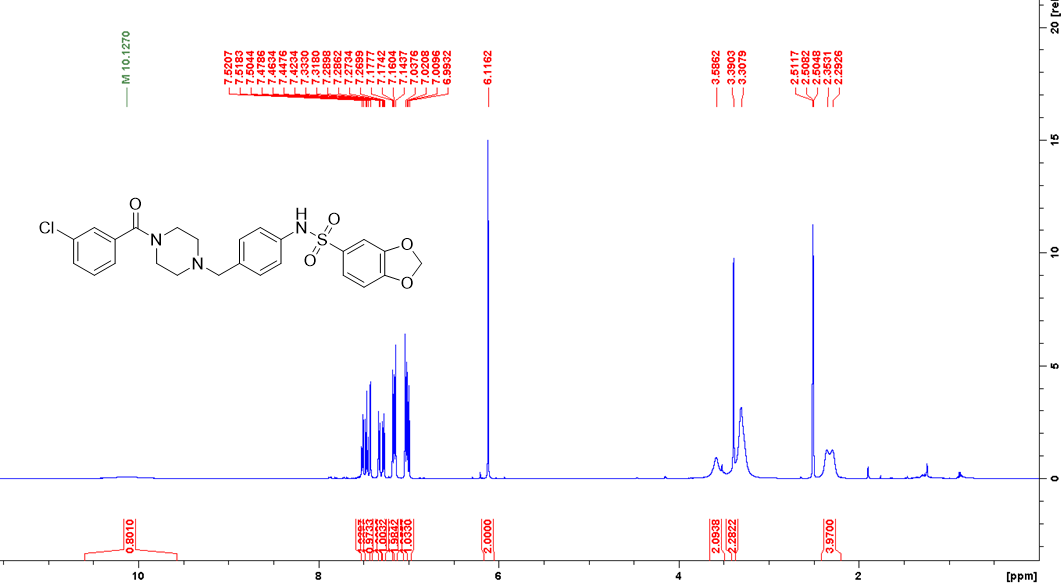


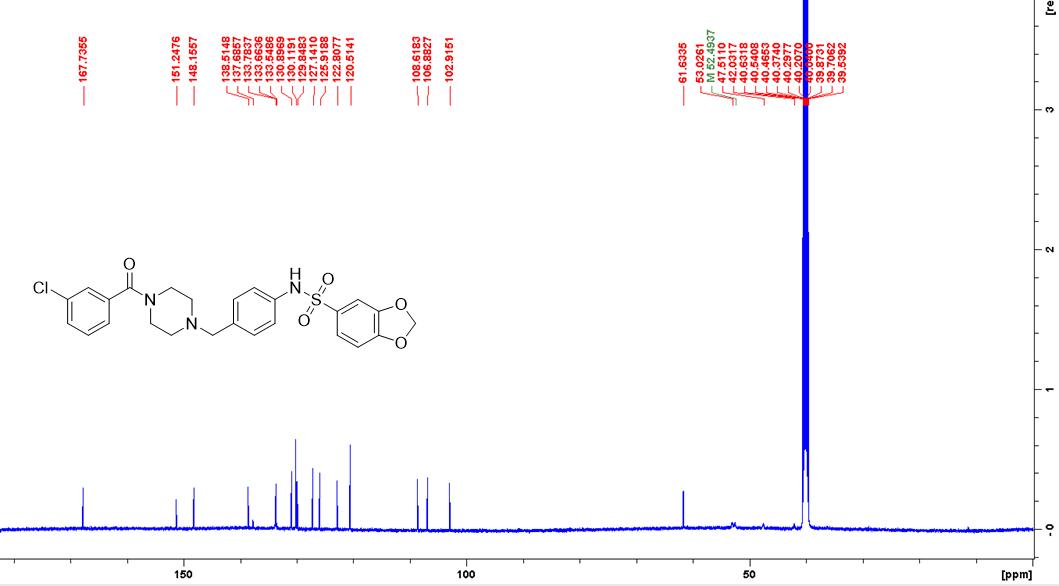


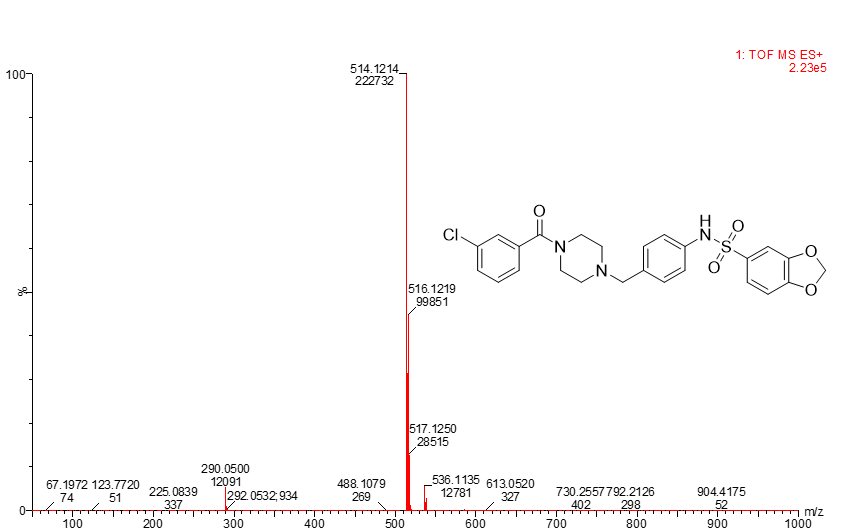


**^1^H, ^13^C NMR and HRMS spectra of compound 7**

**
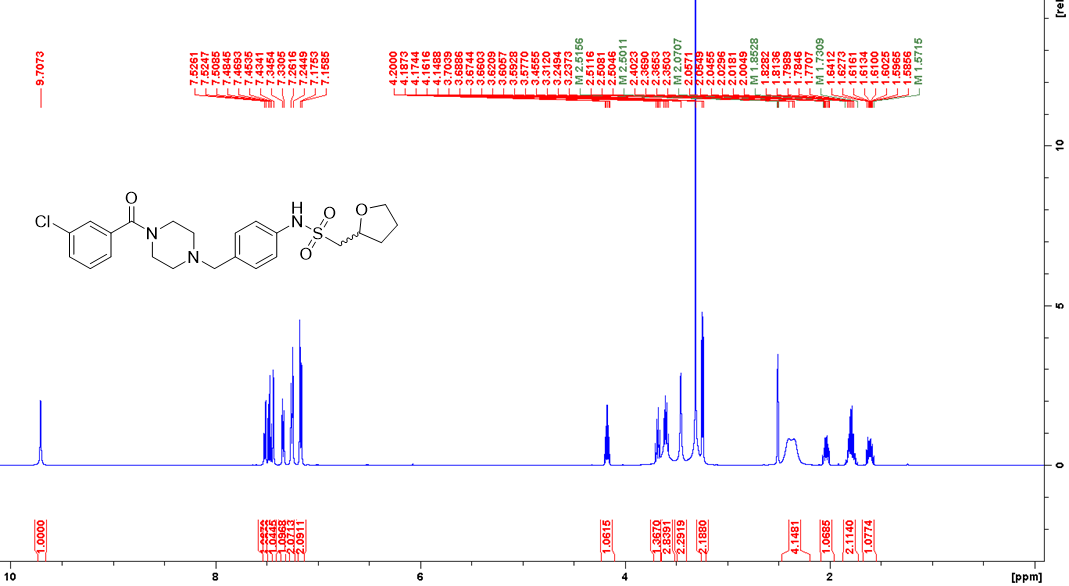
**

**
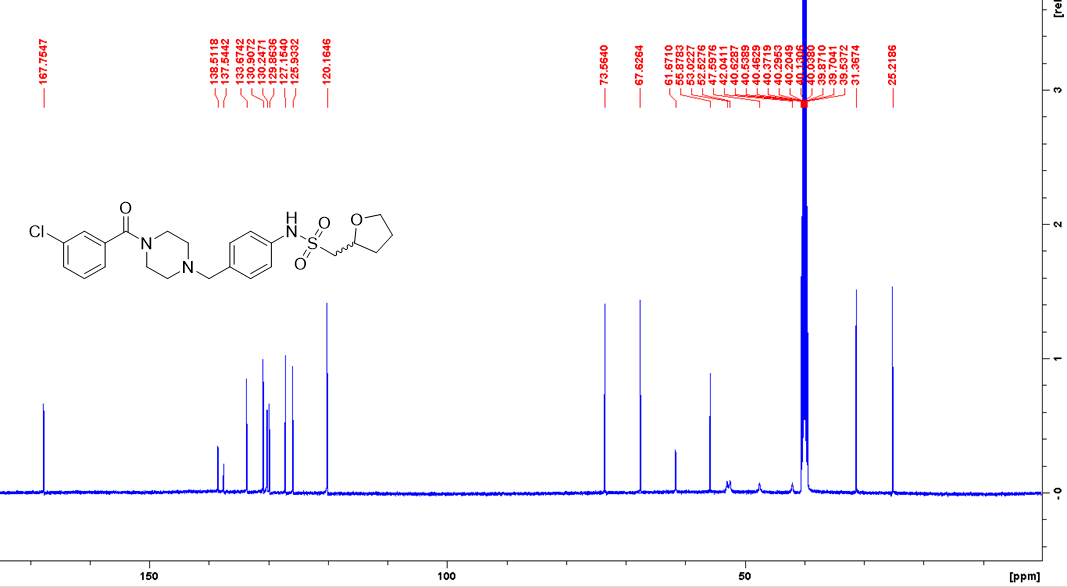
**


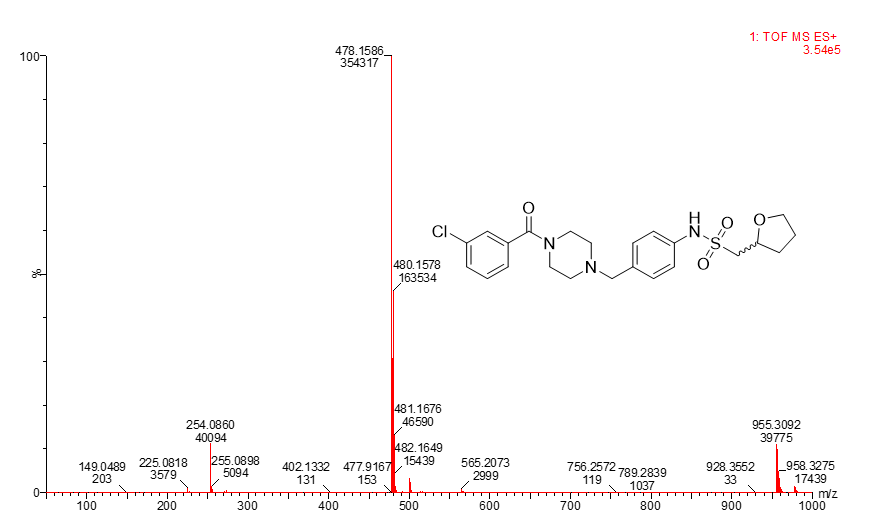


**^1^H, ^13^C NMR and HRMS spectra of compound 8**

**^
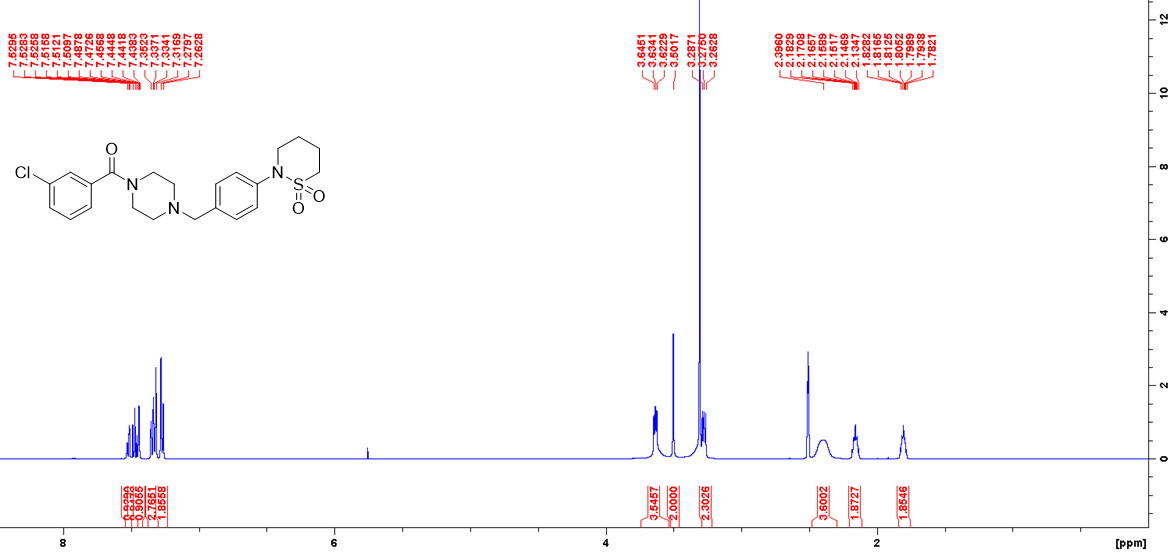
^**

**^
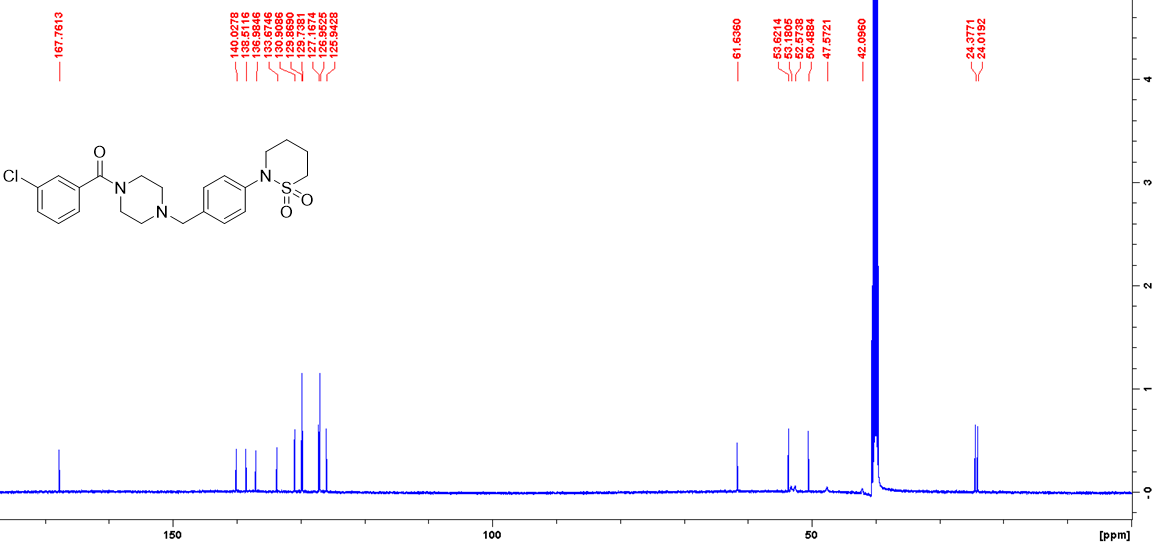
^**

**^
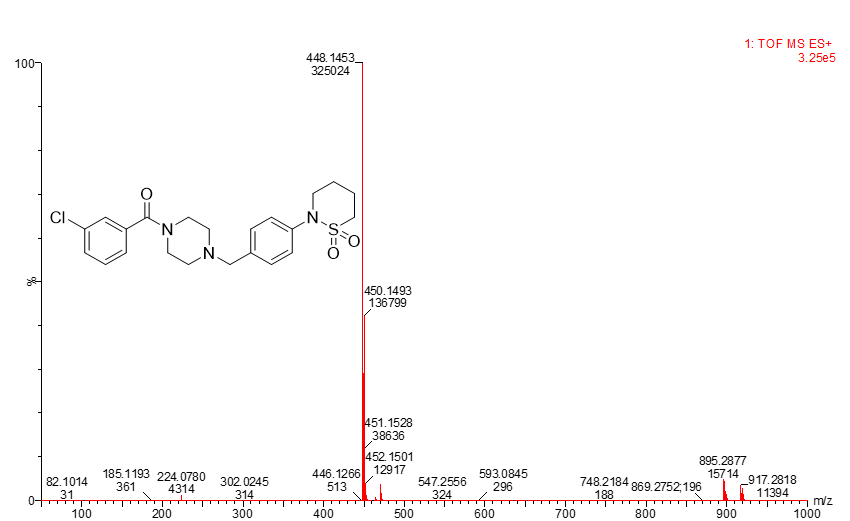
^**

**^1^H, ^13^C NMR and HRMS spectra of compound 9**

**
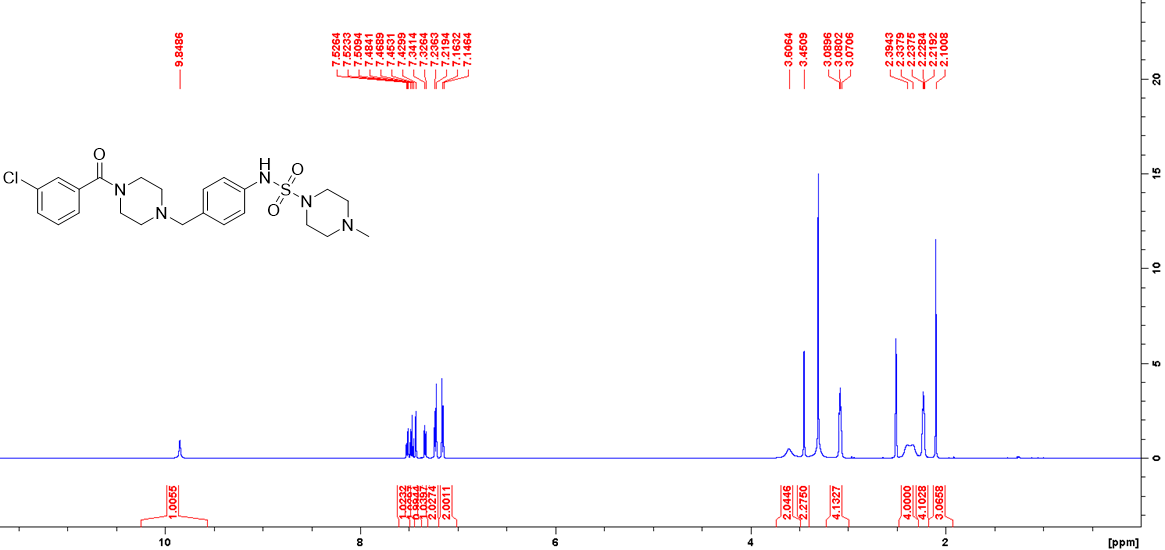
**

**^
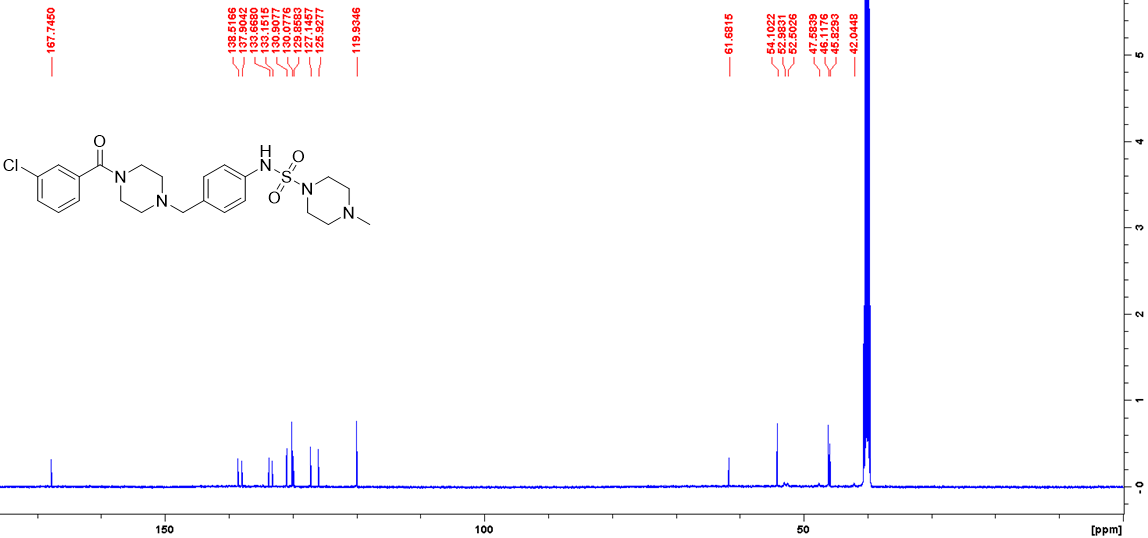
^**

**^
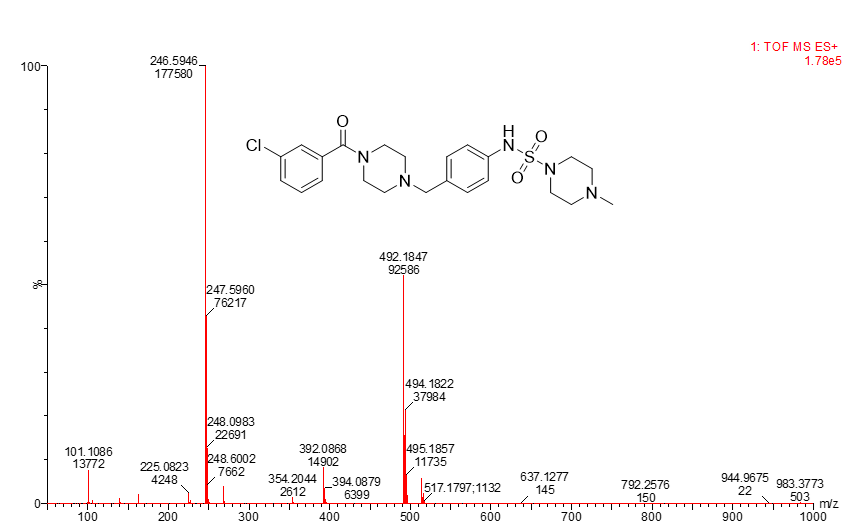
^**

**^1^H, ^13^C NMR and HRMS spectra of compound 10**

**
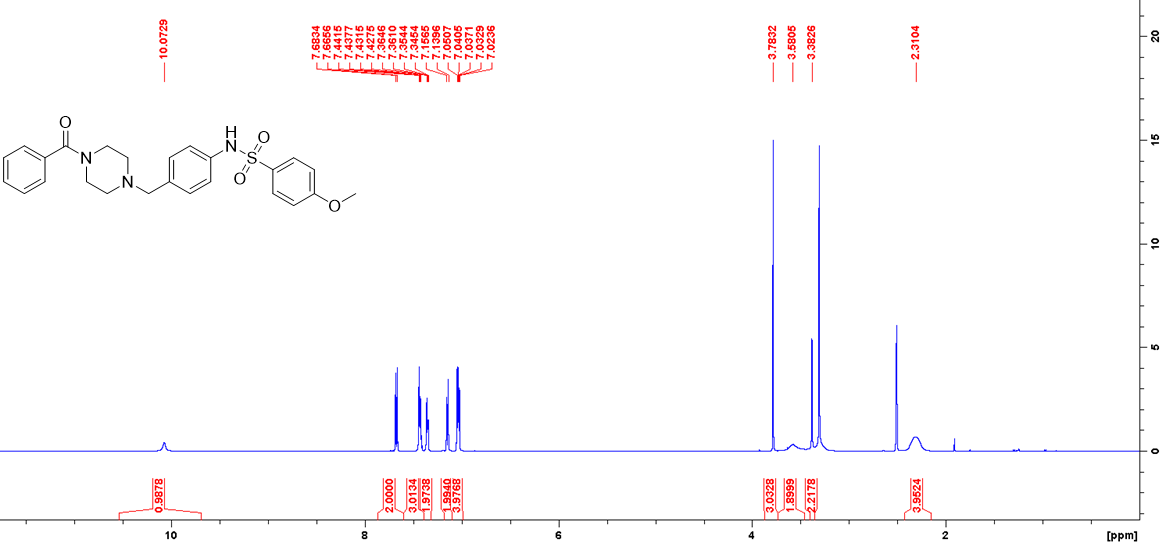
**

**
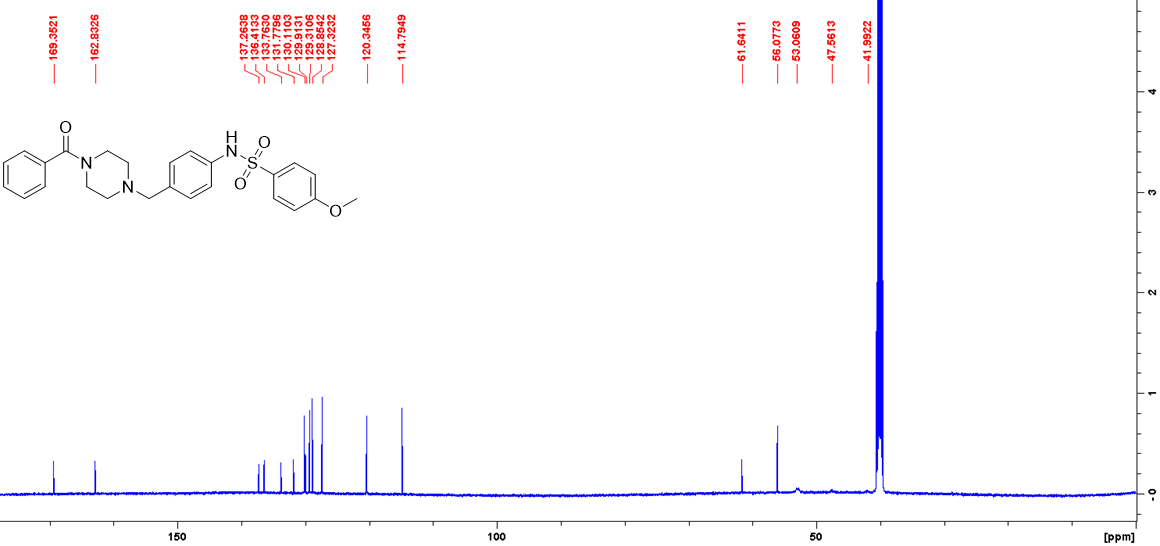
**

**^
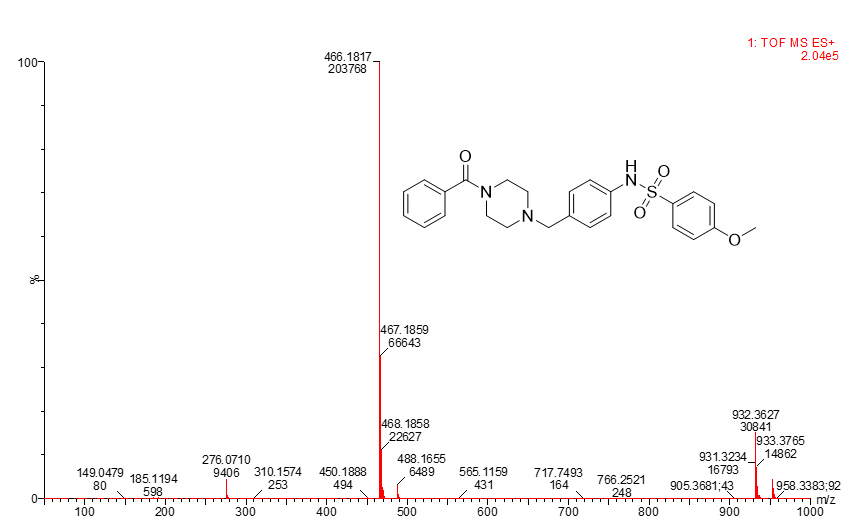
^**

**^1^H, ^13^C NMR and HRMS spectra of compound 13**

**^
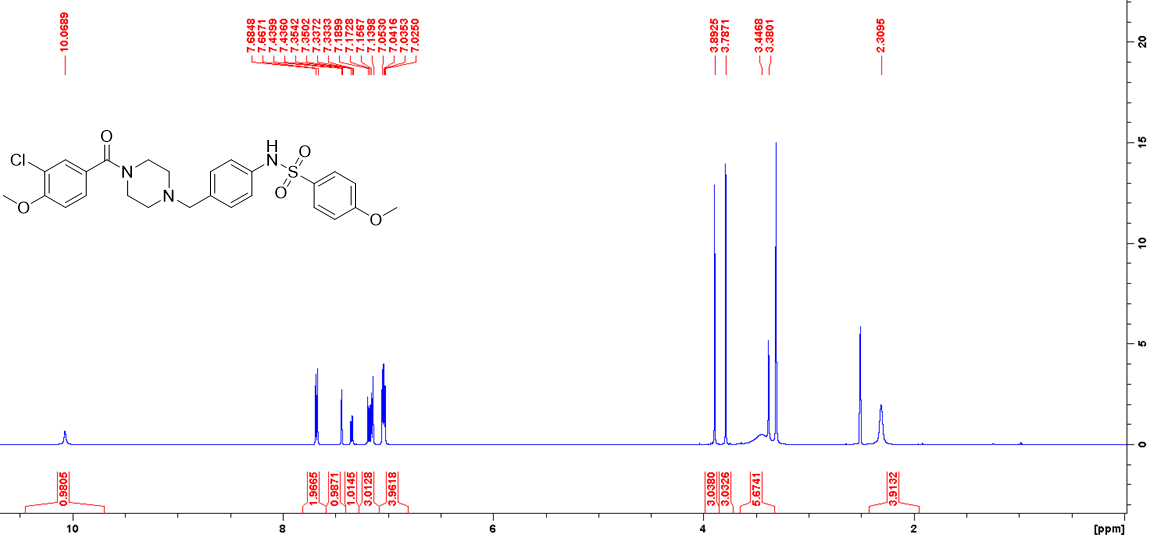
^**

**^
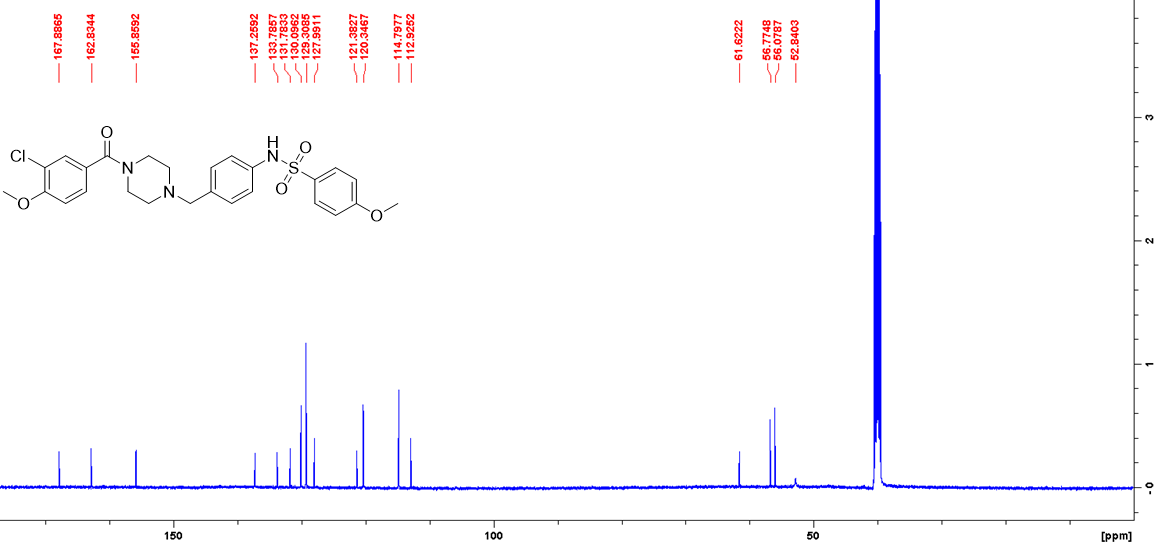
^**

**^
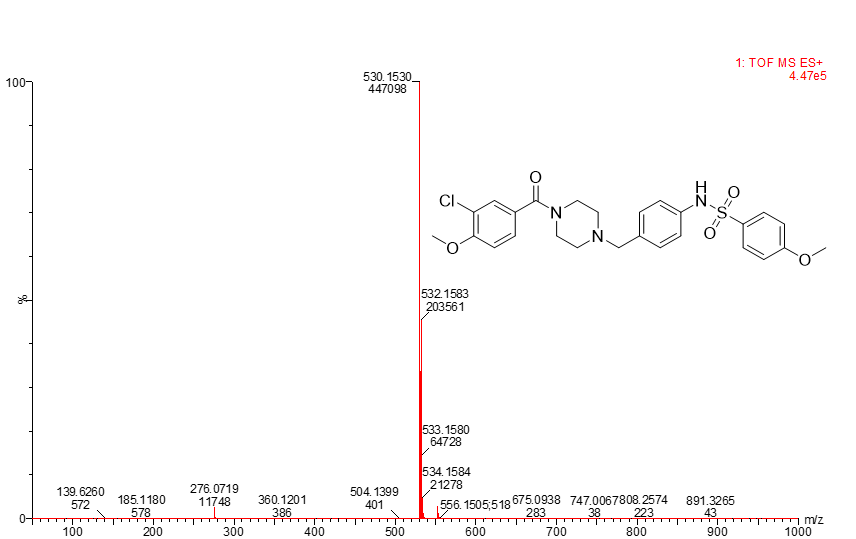
^**

**^1^H, ^13^C NMR and HRMS spectra of compound 14**

**^
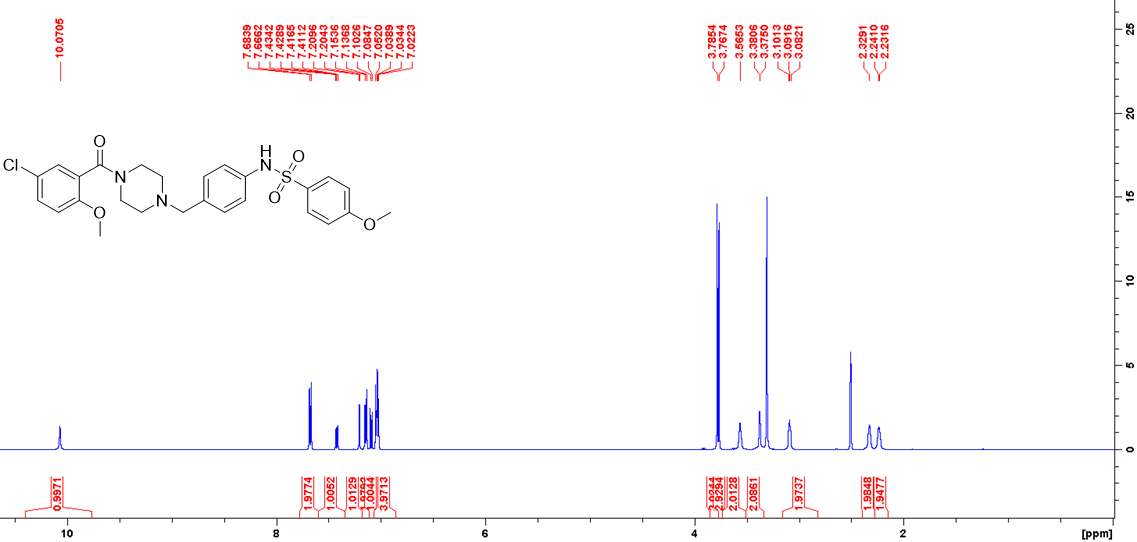
^**

**^
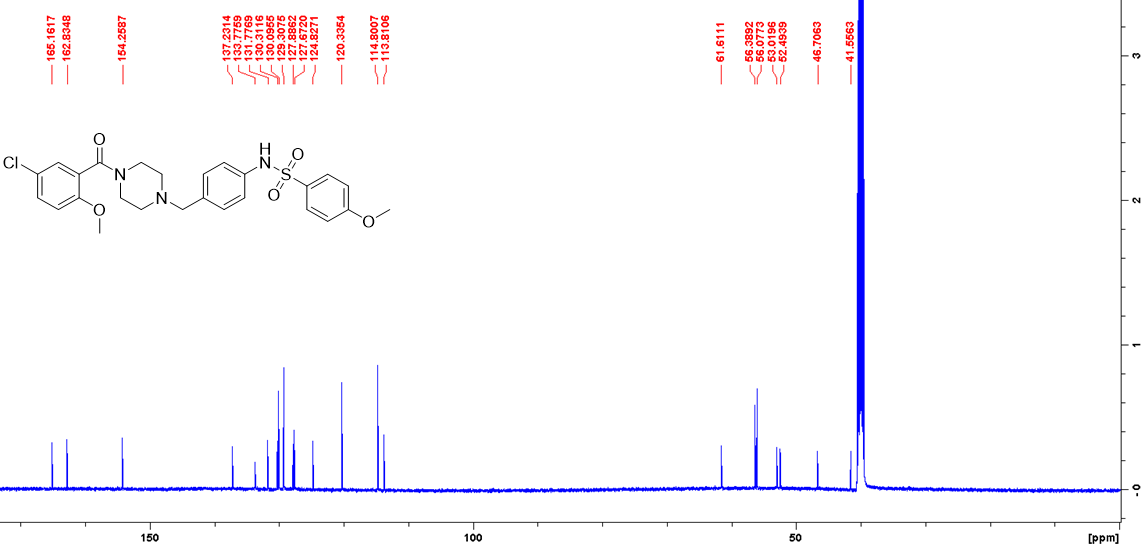
^**

**^
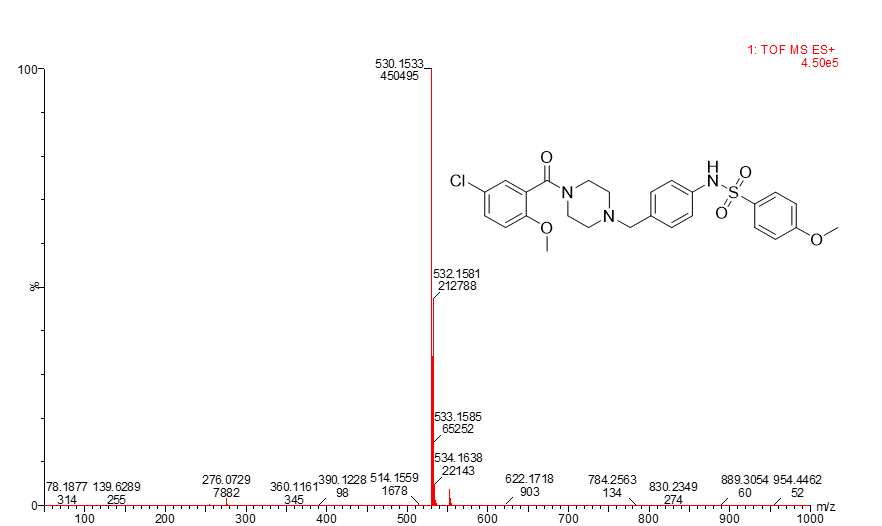
^**

**^1^H, ^13^C NMR and HRMS spectra of compound 15**

**^
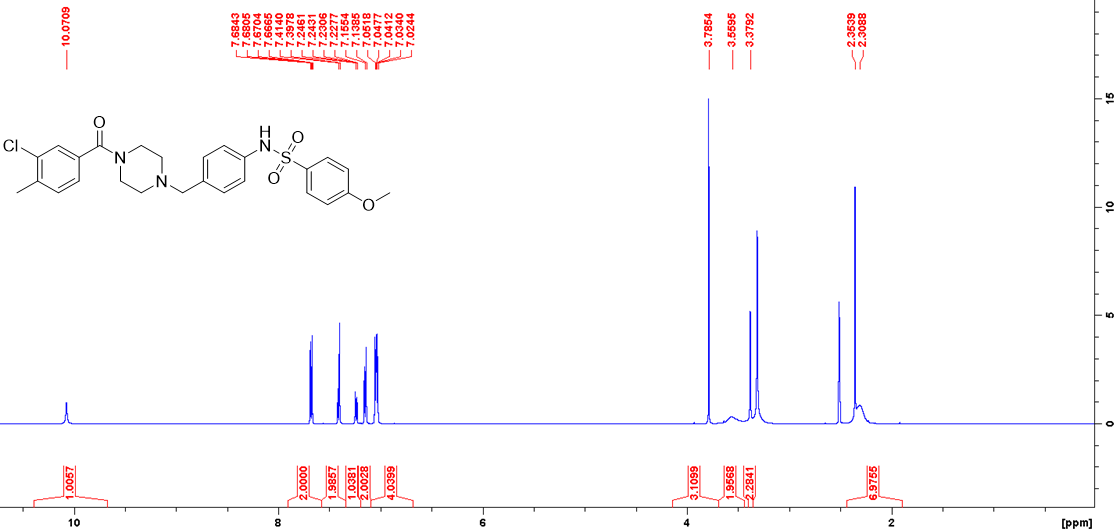
^**

**^
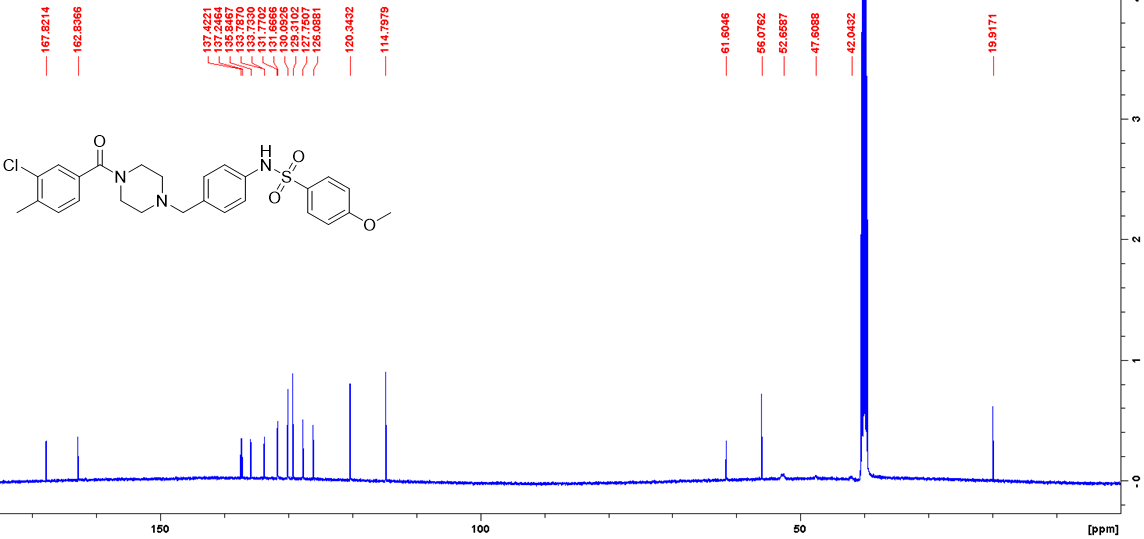
^**

**^
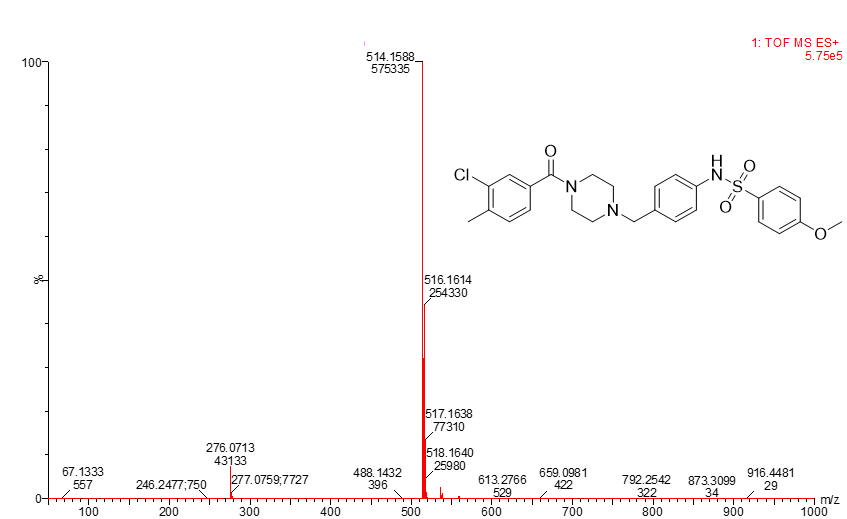
^**

**^1^H, ^13^C NMR and HRMS spectra of compound 16**

**^
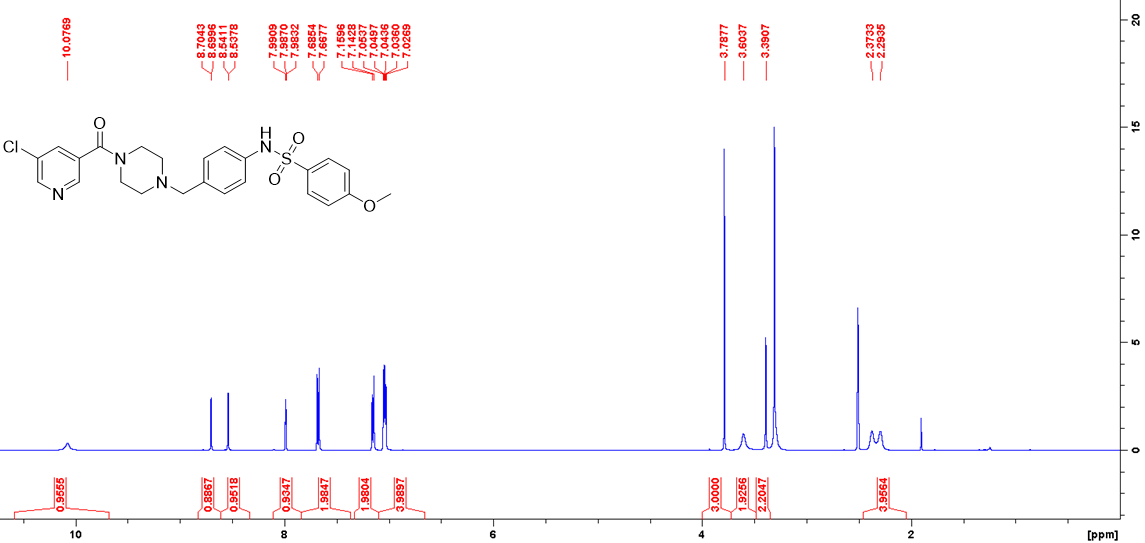
^**

**^
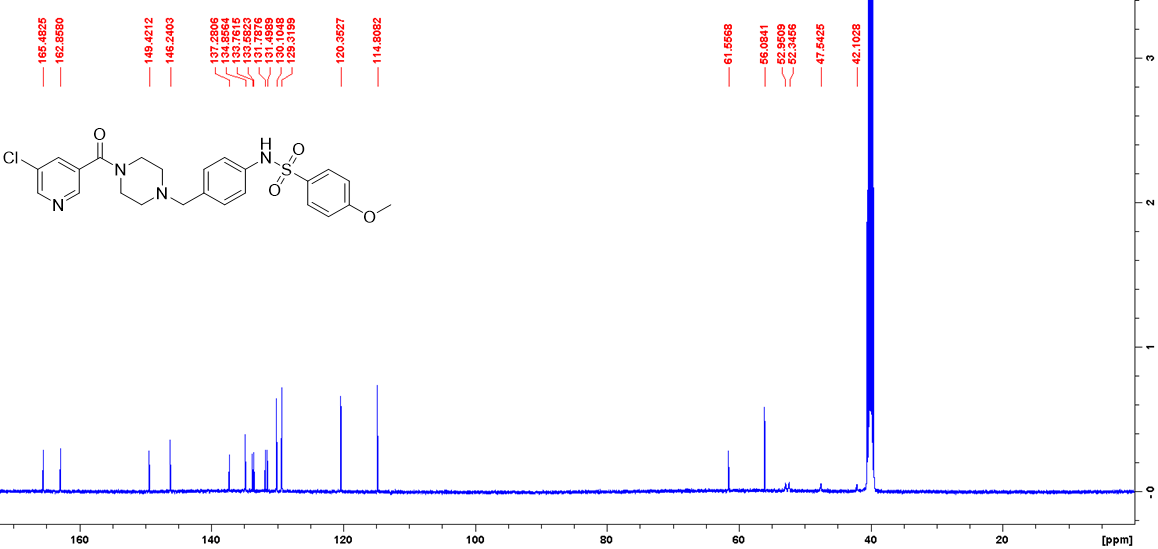
^**

**^
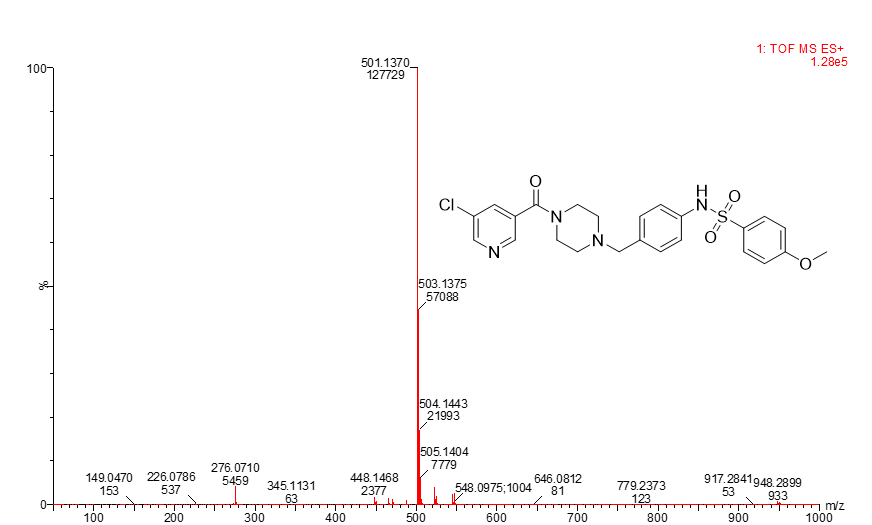
^**

**^1^H NMR spectrum of compound 17**

**
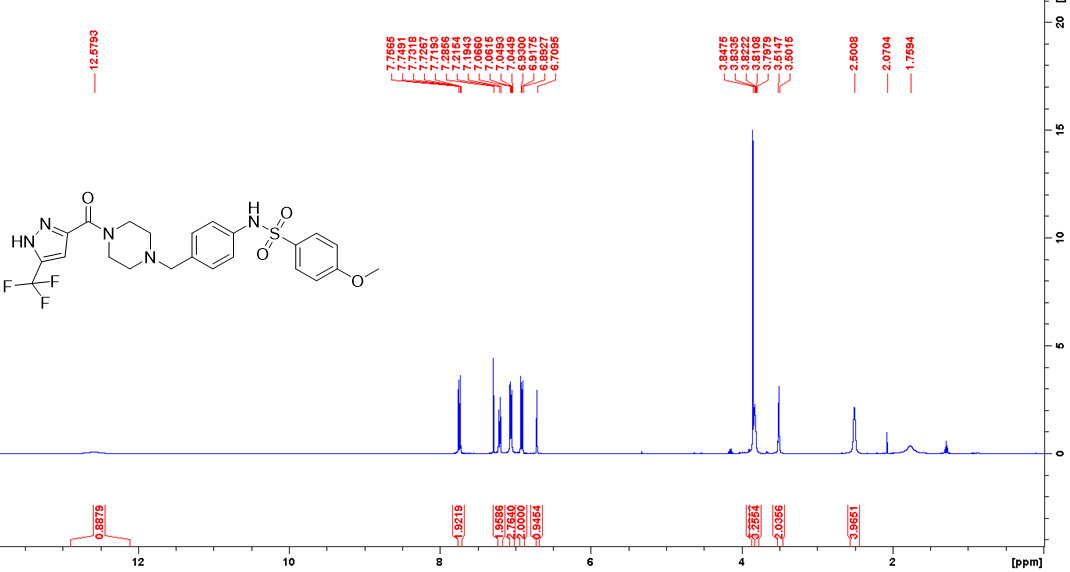
**

**^1^H, ^13^C NMR and HRMS spectra of compound 18**

**^
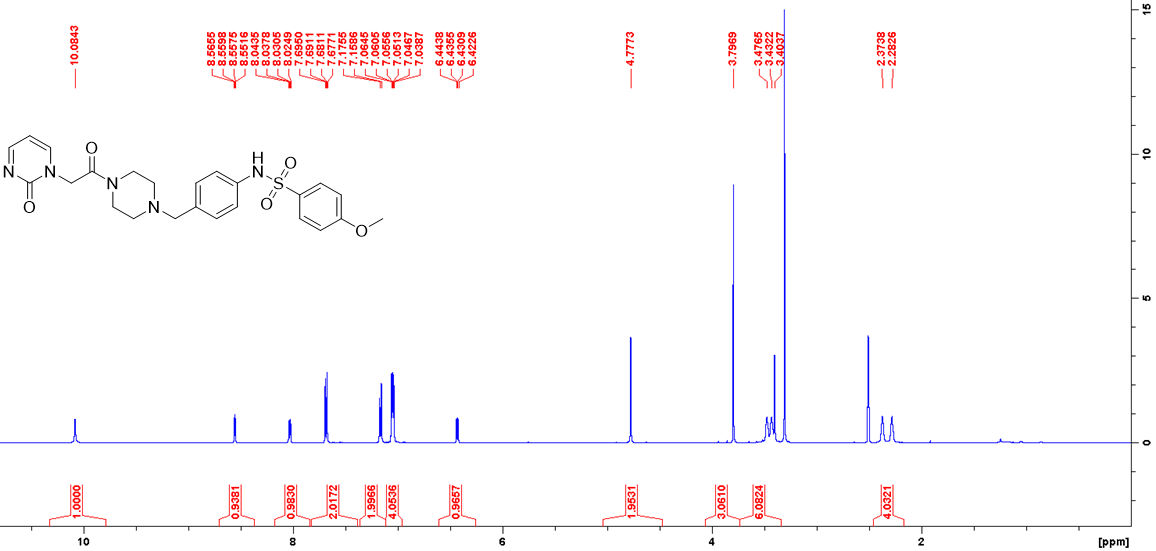
^**

**^
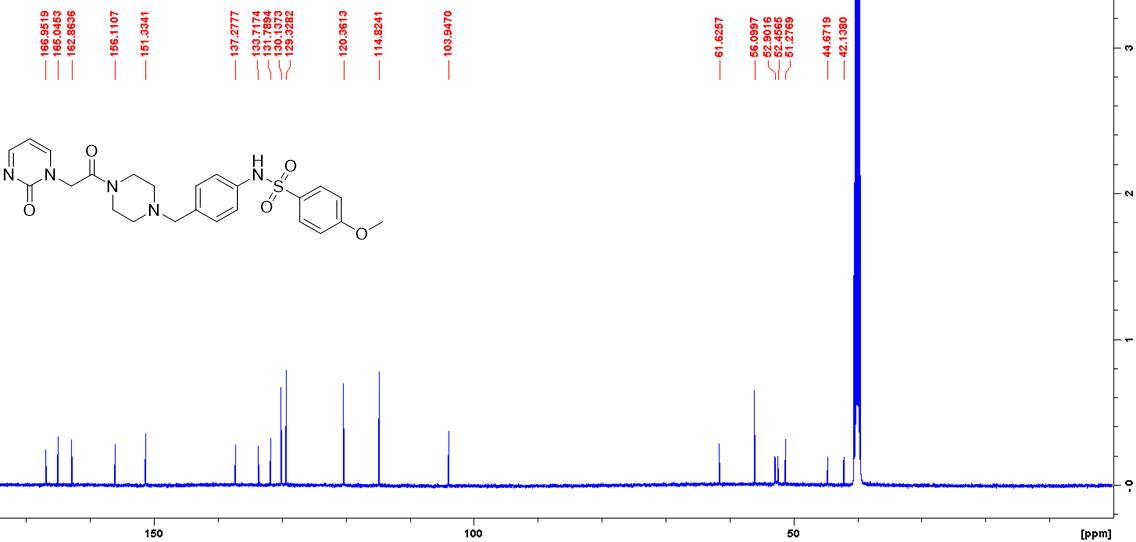
^**

**^
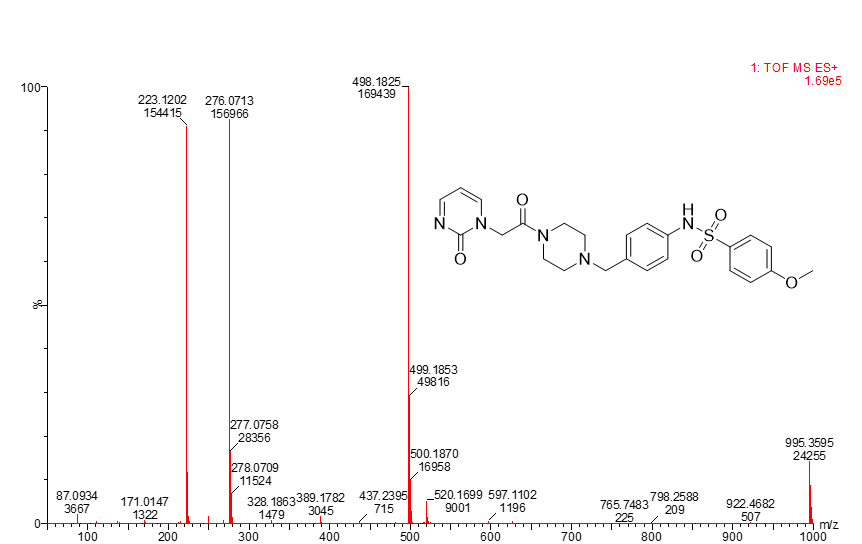
^**

**^1^H NMR spectrum of compound 19**


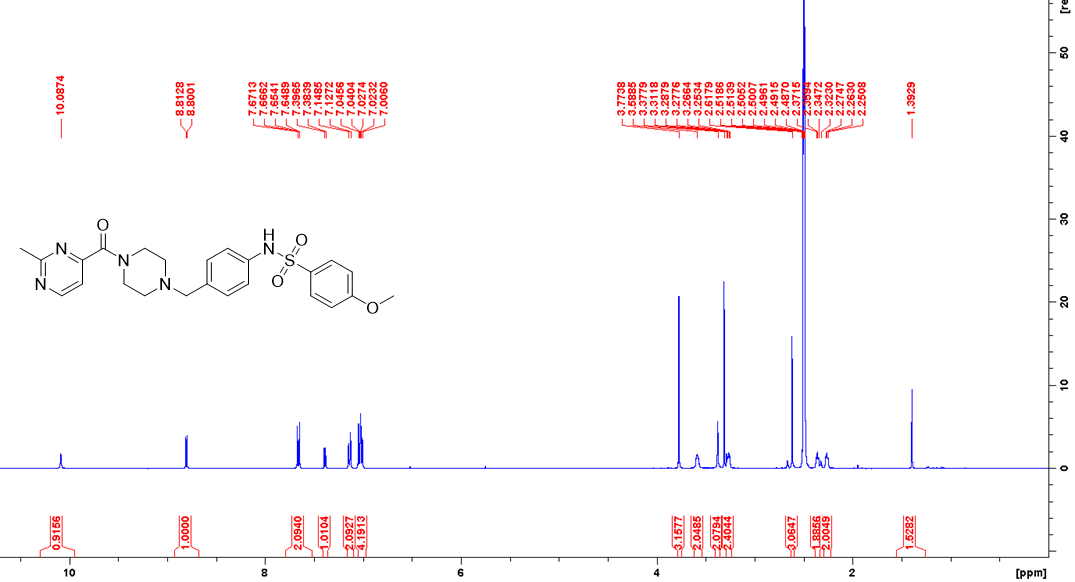


**^1^H, ^13^C NMR and HRMS spectra of compound 20**

**
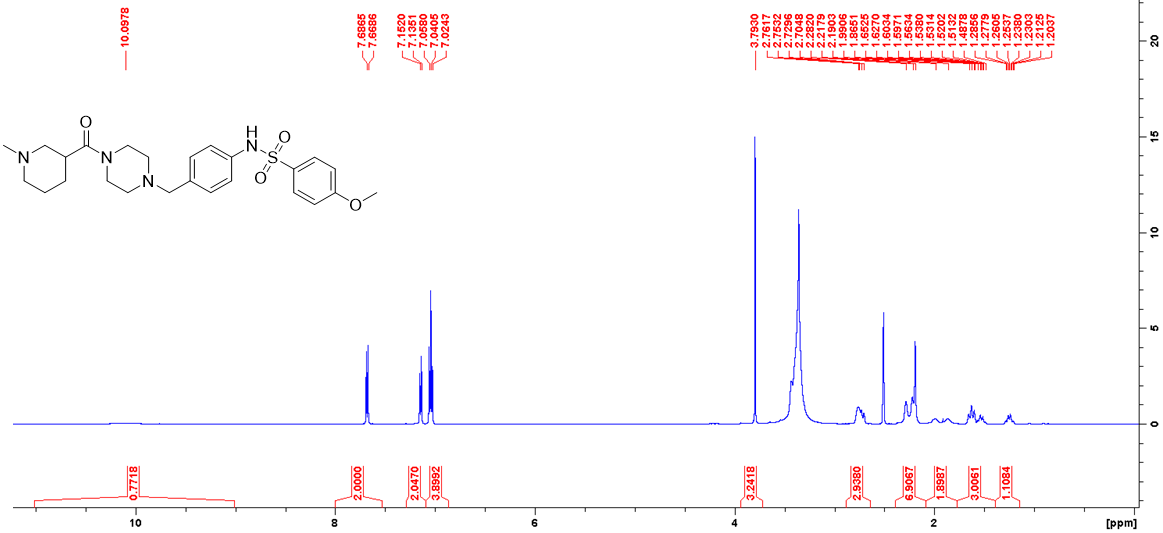
**

**
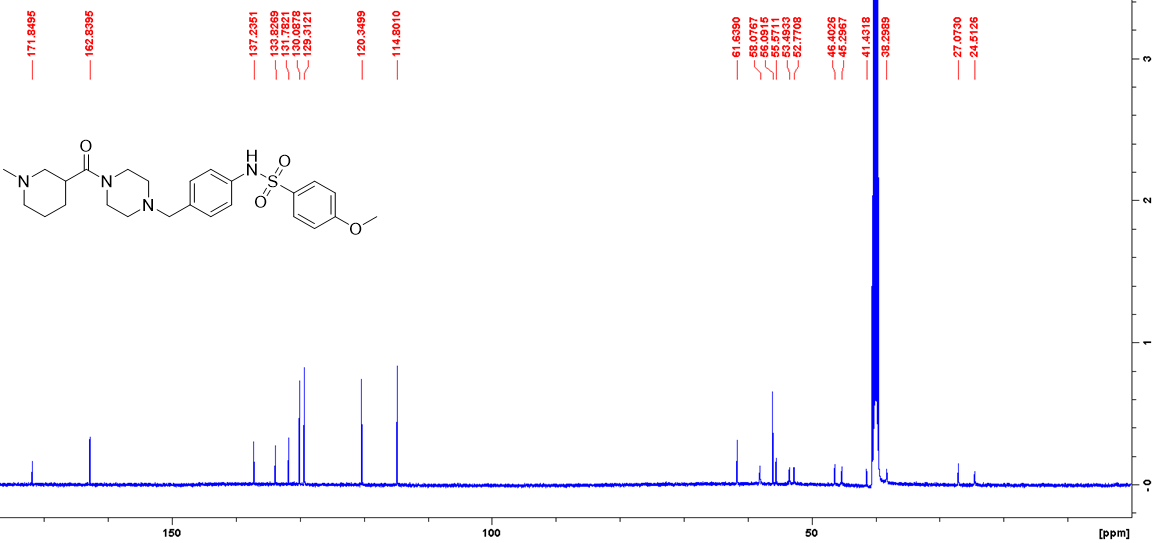
**

**
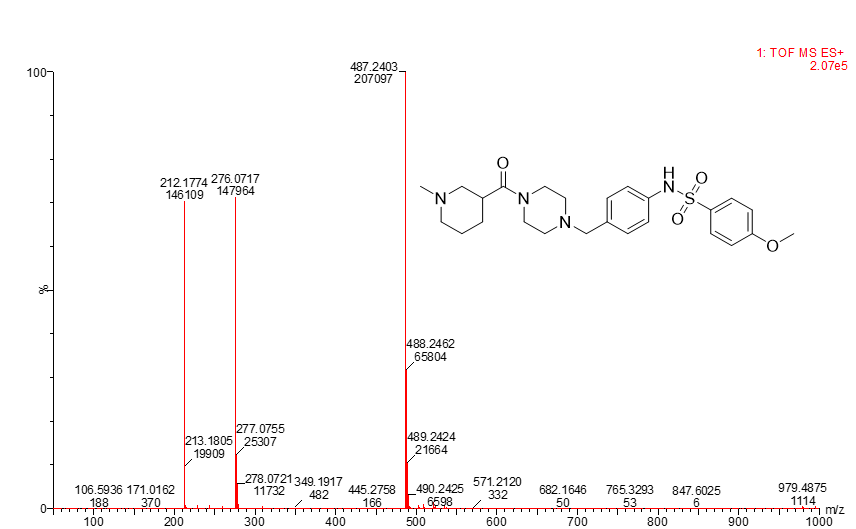
**

**^1^H, ^13^C NMR and HRMS spectra of compound 21**

**^
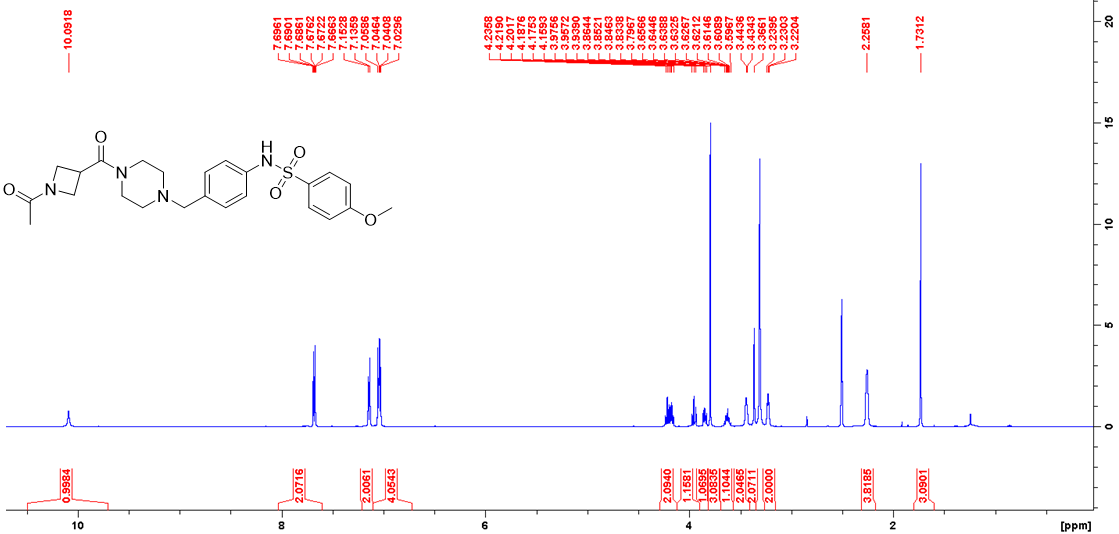
^**

**^
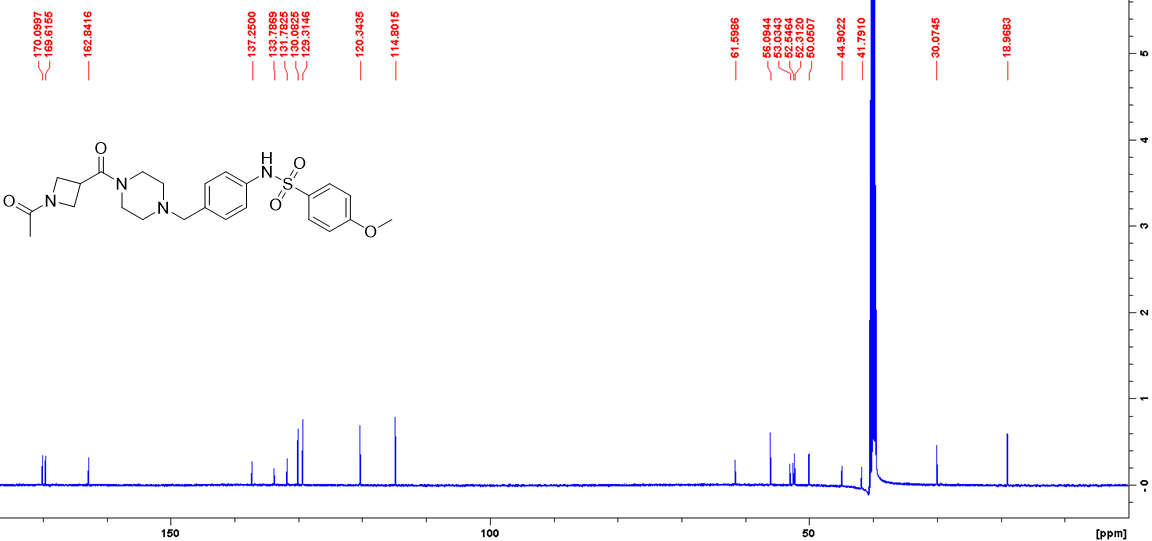
^**

**^
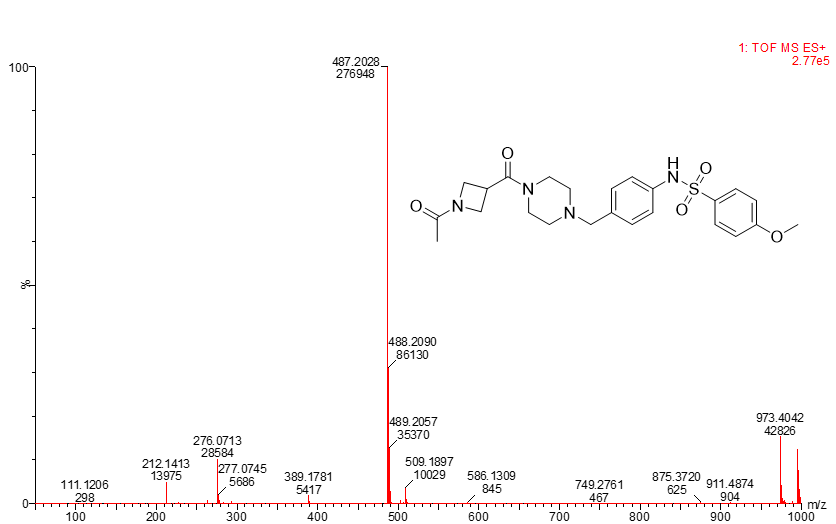
^**

**^1^H, ^13^C NMR and HRMS spectra of compound 22**

**
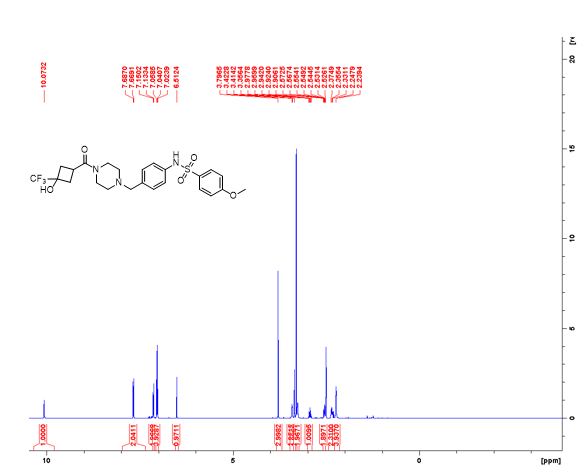
**

**
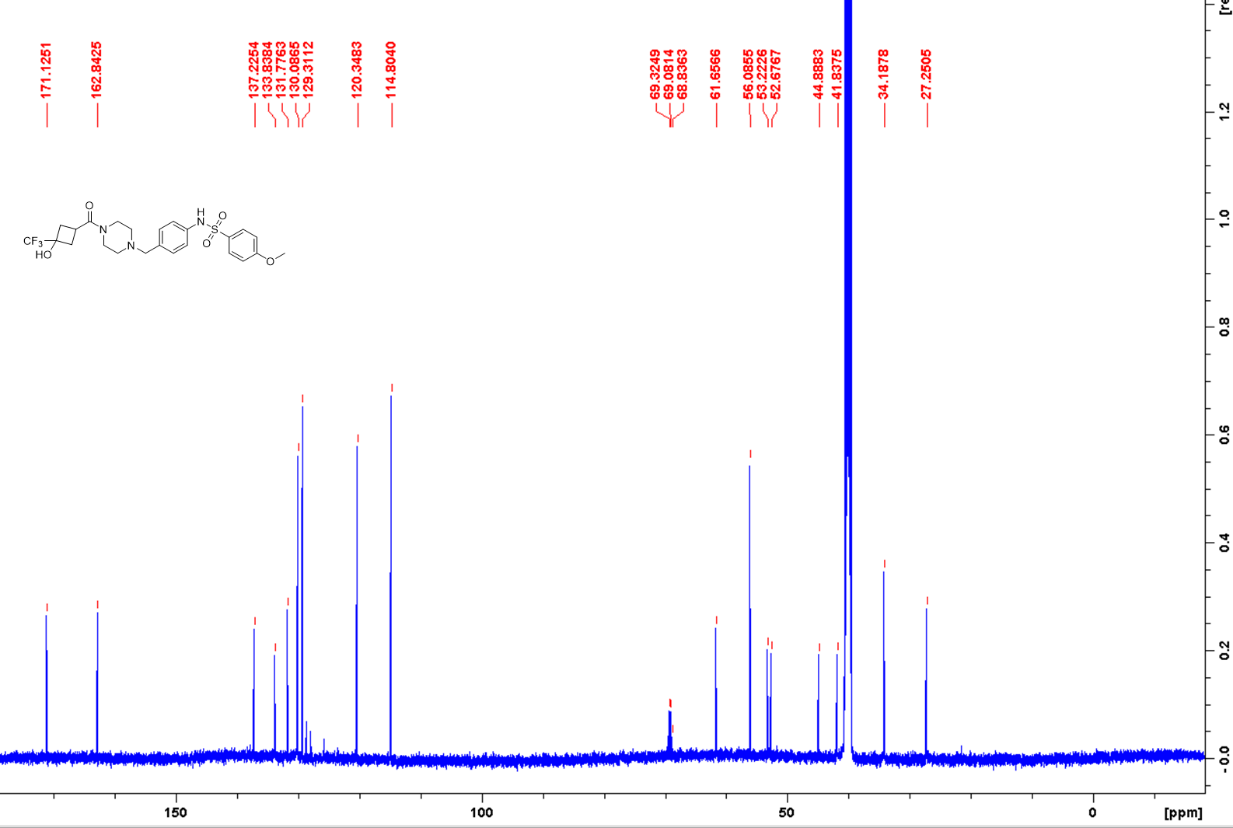
**

**
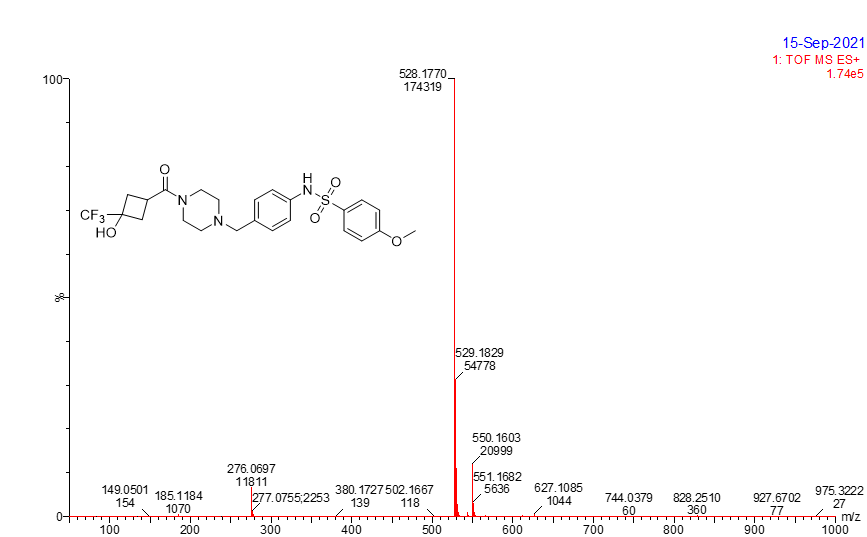
**

**^1^H, ^13^C NMR and HRMS spectra of compound 23**

**
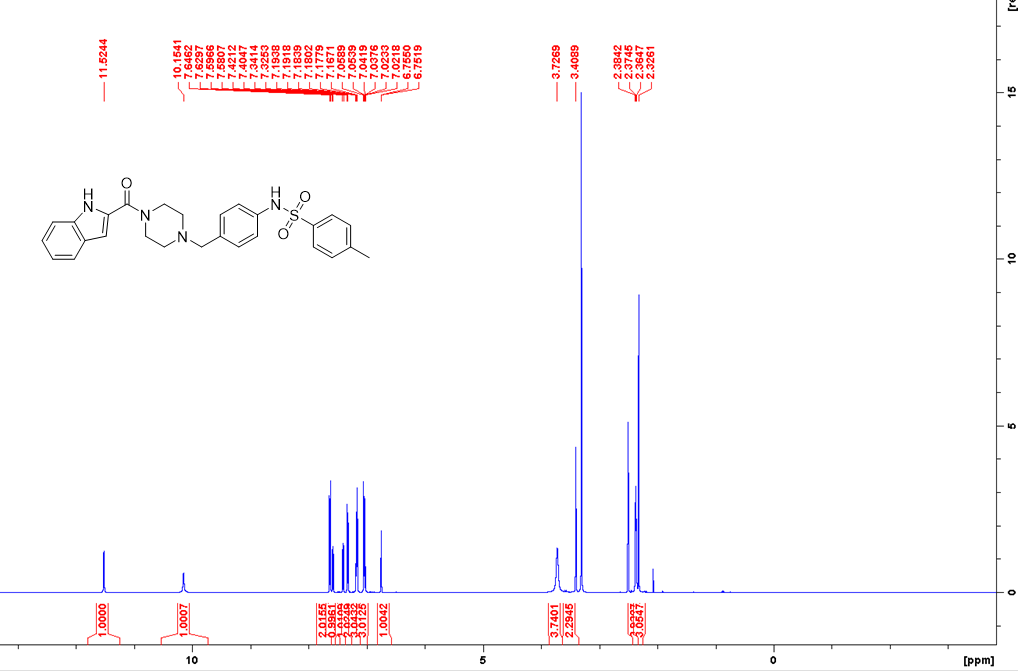
**

**
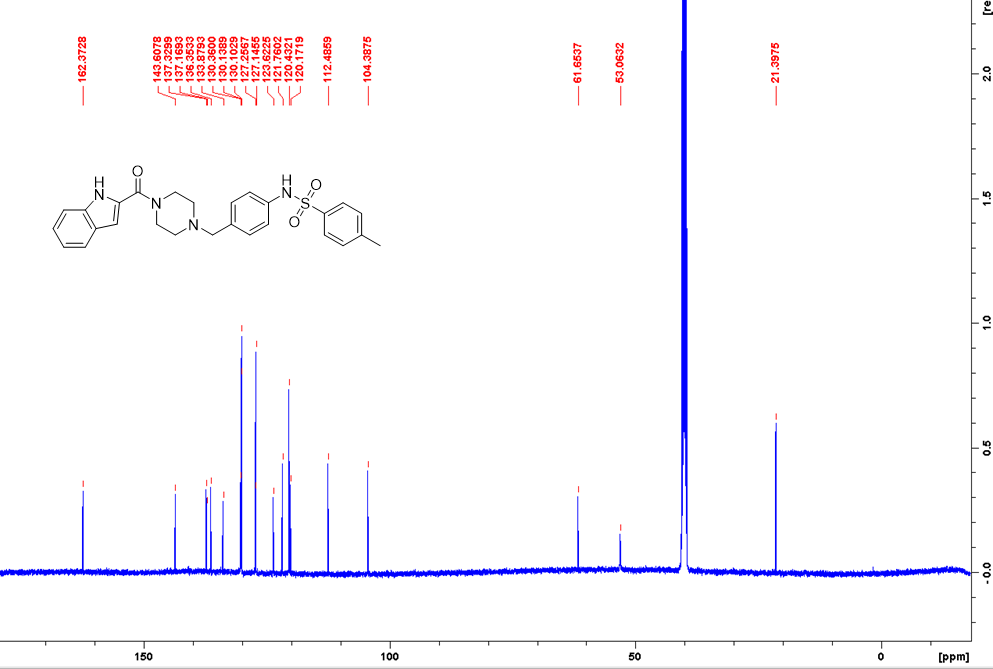
**

**
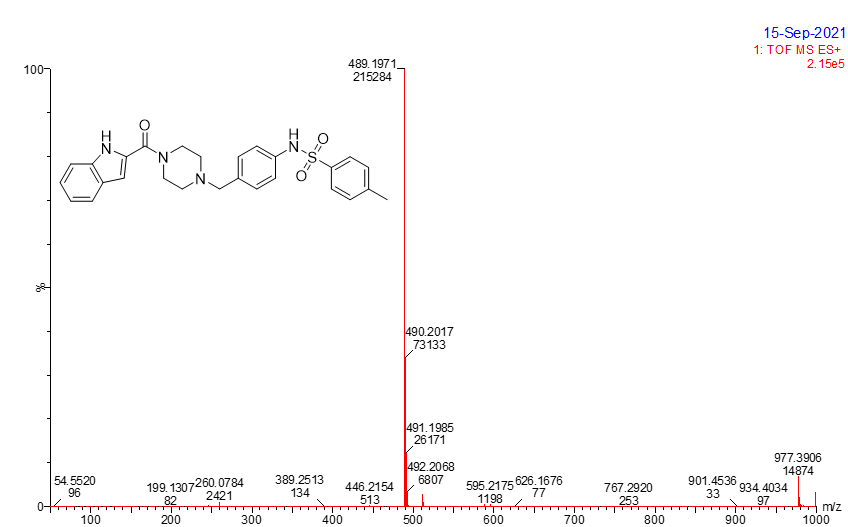
**

**^1^H, ^13^C NMR and HRMS spectra of compound 24**


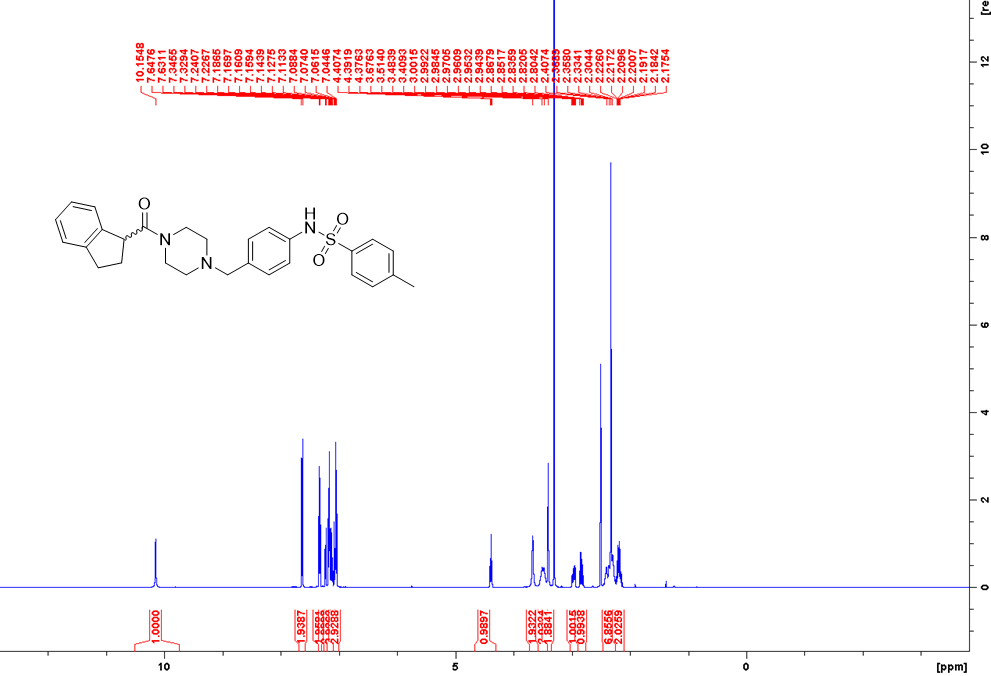


**
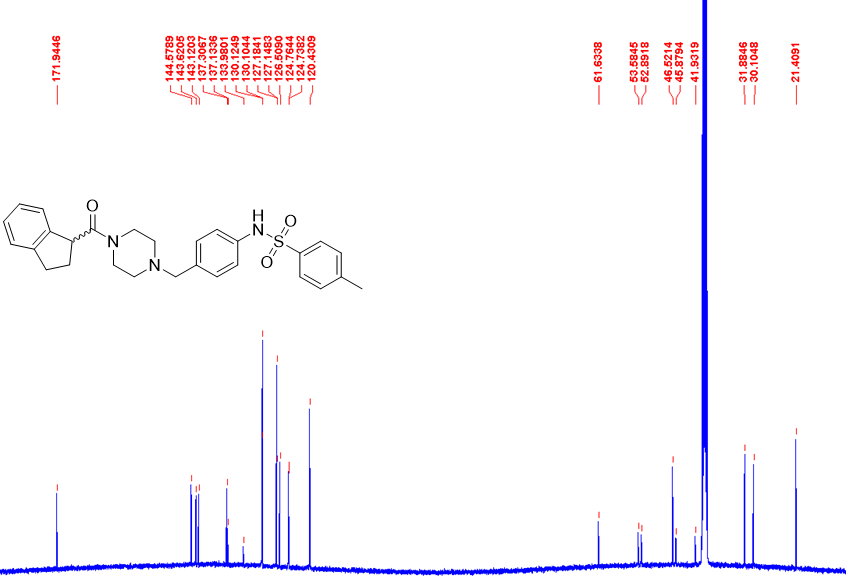
**

**
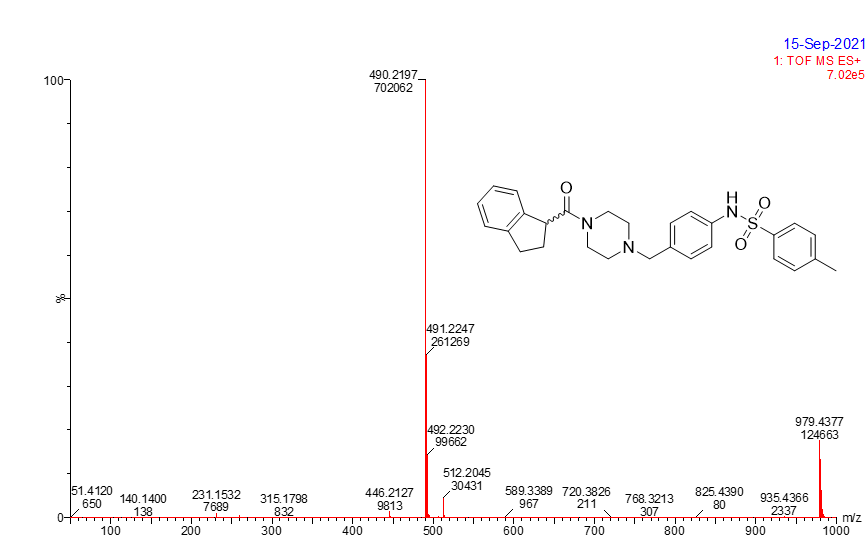
**

**^1^H, ^13^C NMR and HRMS spectra of compound 25**


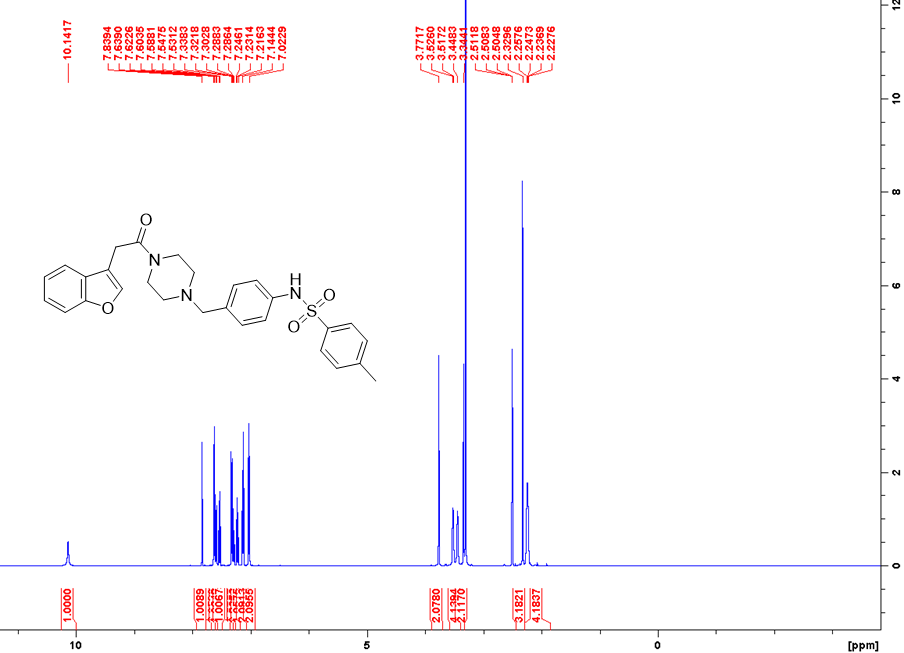


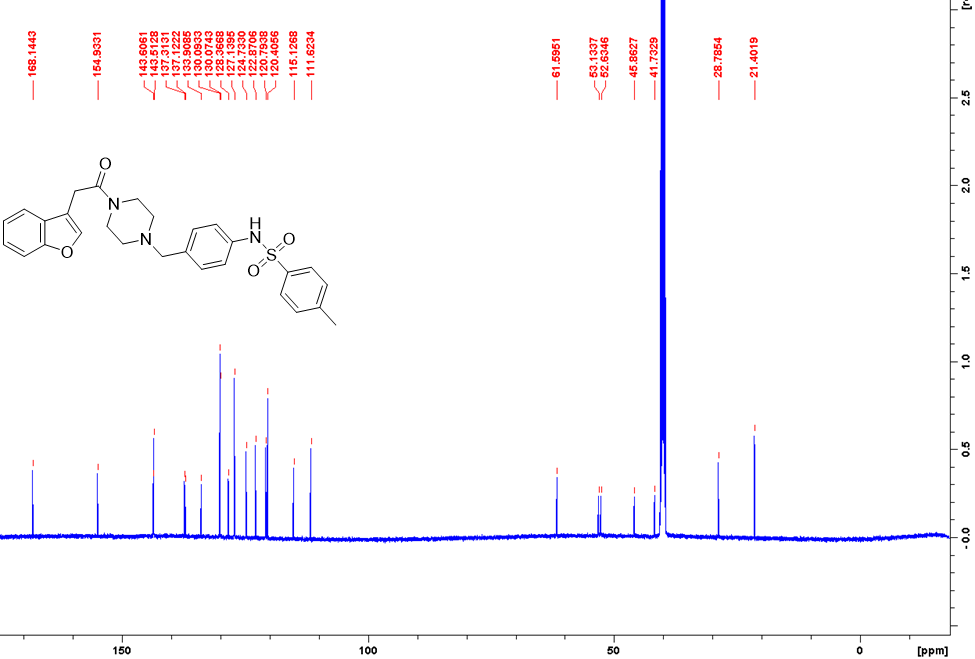


**
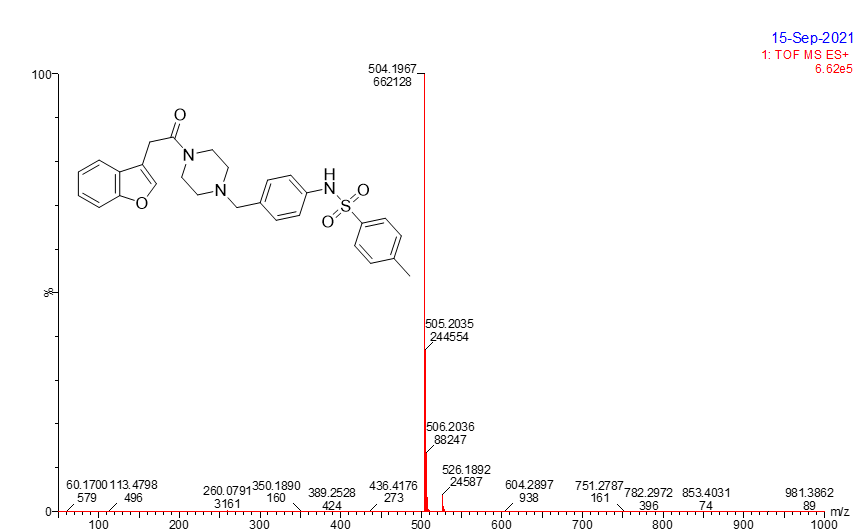
**

**^1^H, ^13^C NMR and HRMS spectra of compound 26**


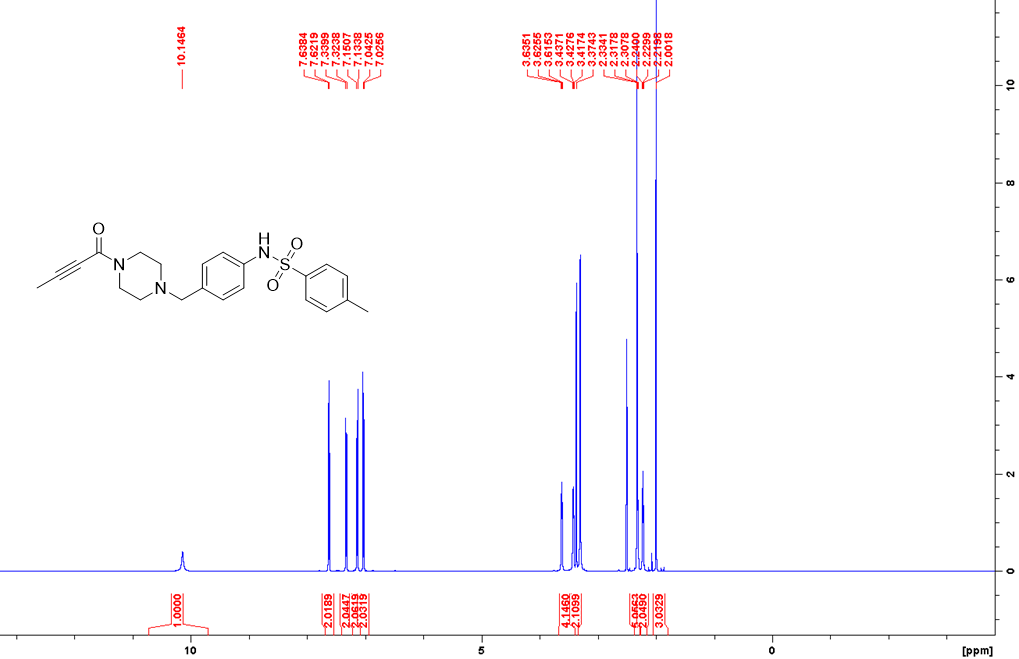


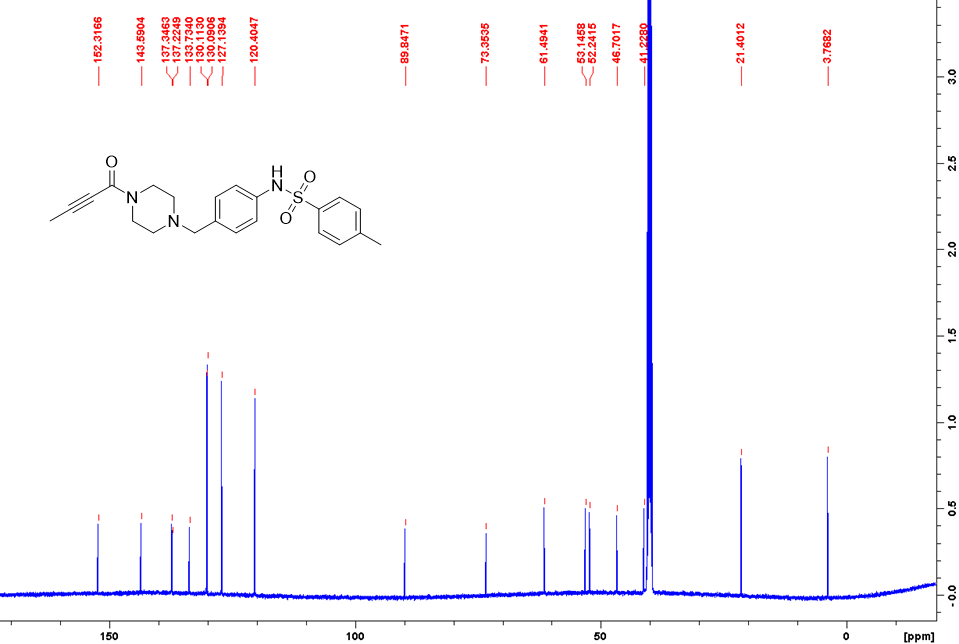


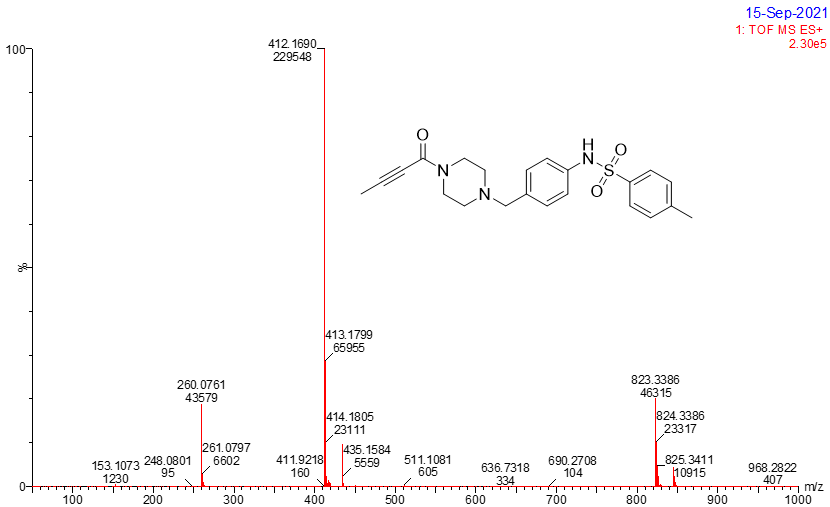


**^1^H NMR spectrum of compound 27**


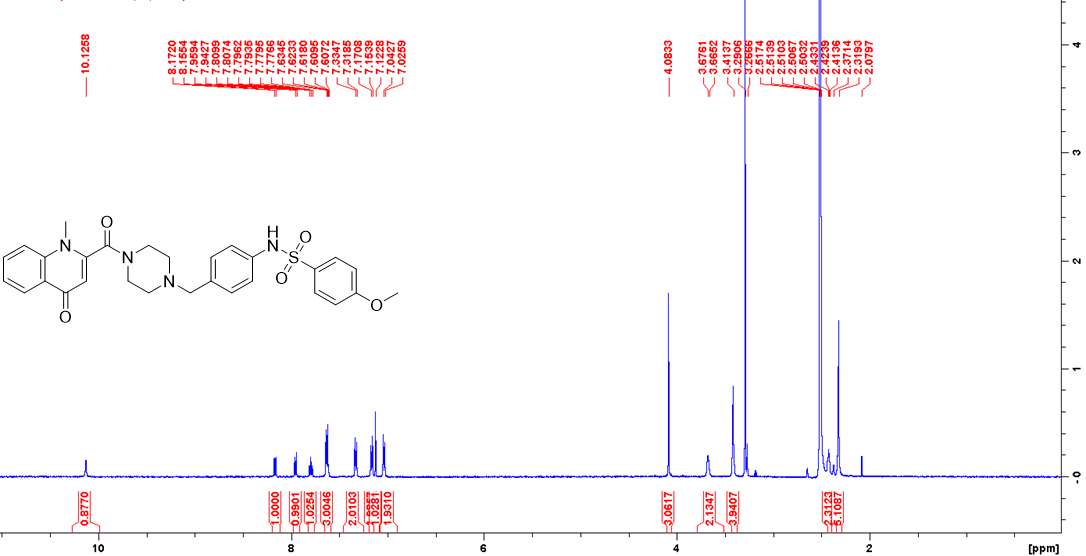


**^1^H, ^13^C NMR and HRMS spectra of compound 28**


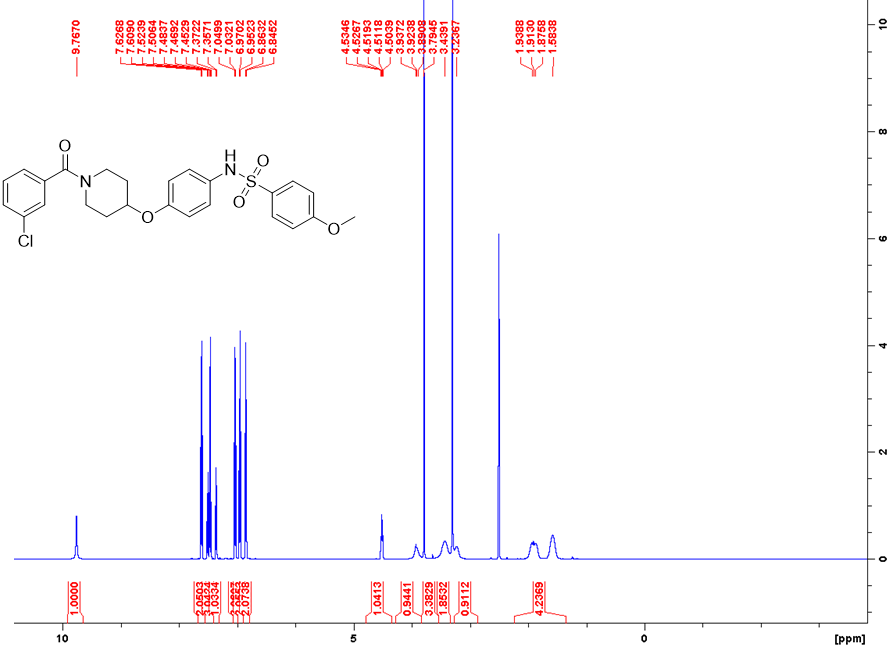


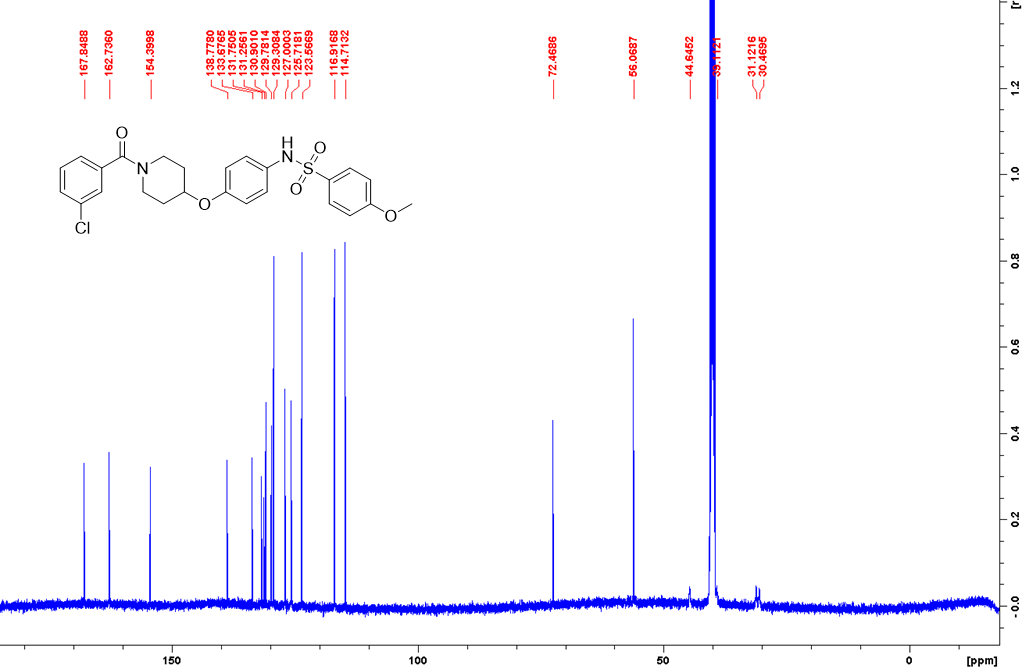
**
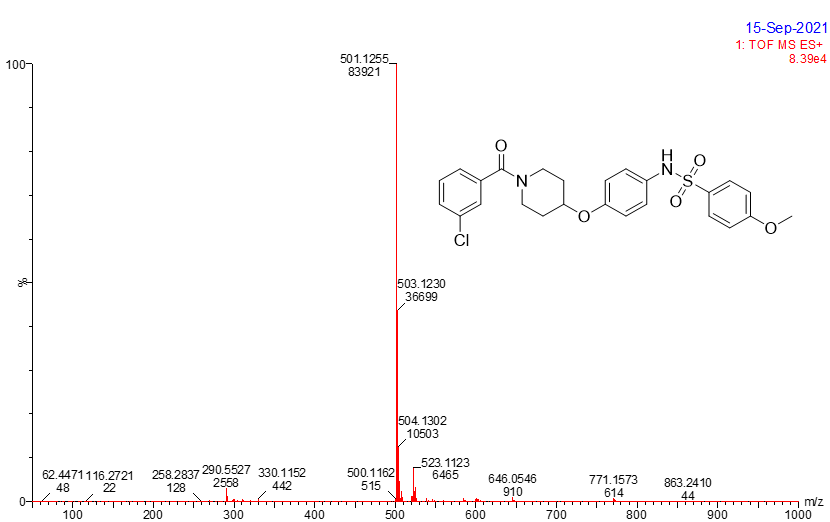
**

**^1^H, ^13^C NMR and HRMS spectra of compound 29**


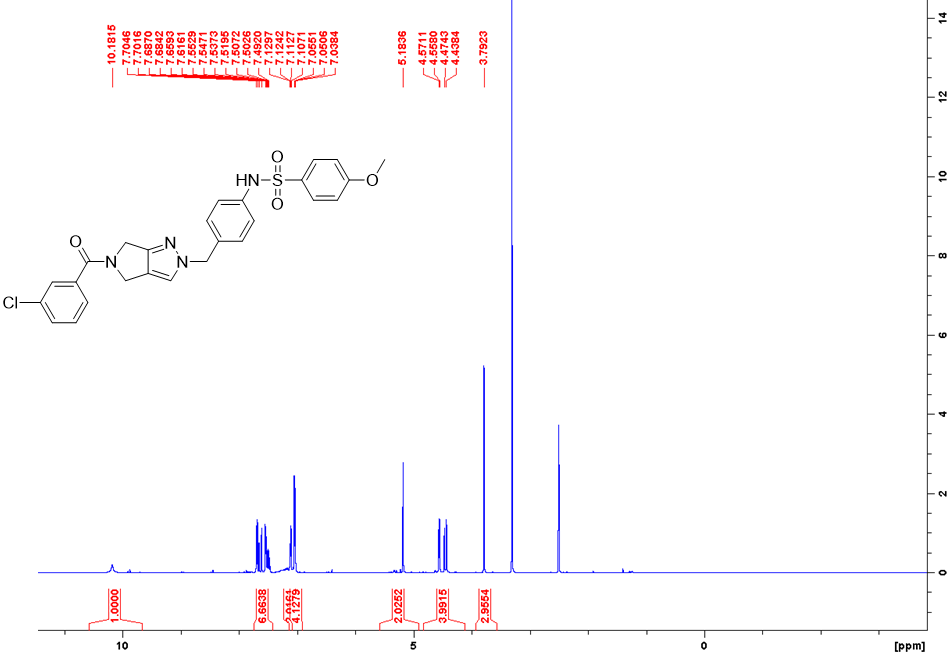


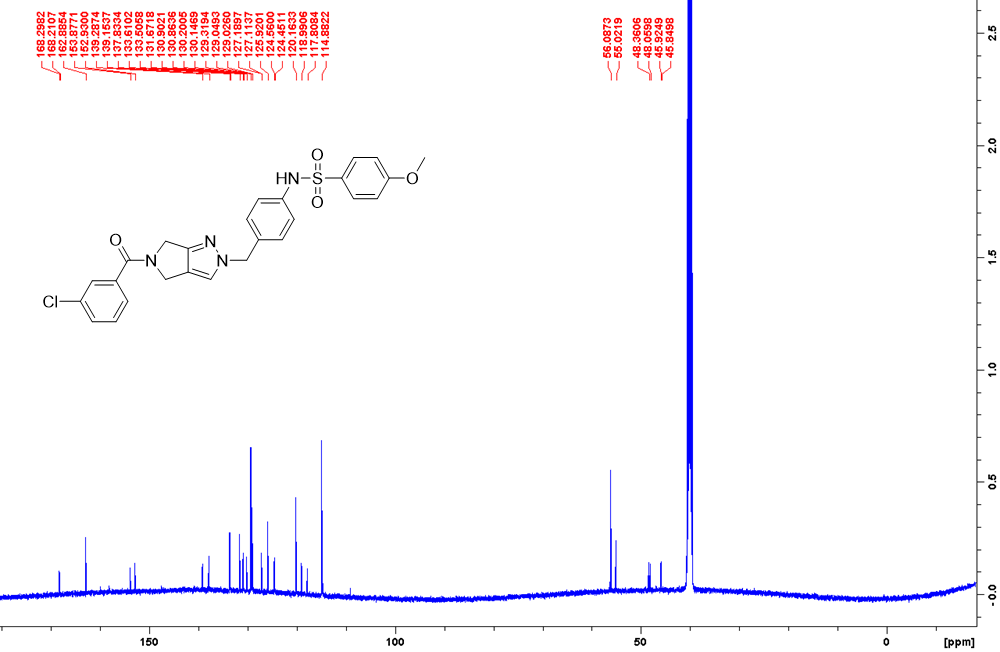


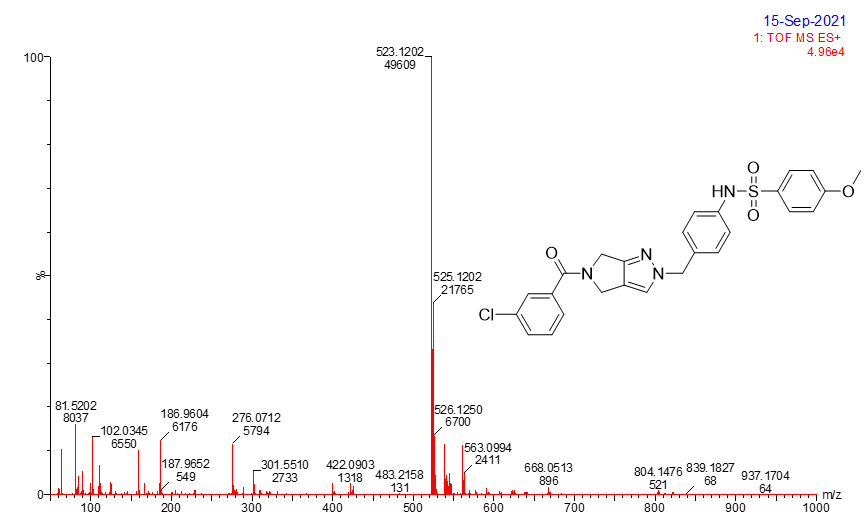


**^1^H, ^13^C NMR and HRMS spectra of compound 30**


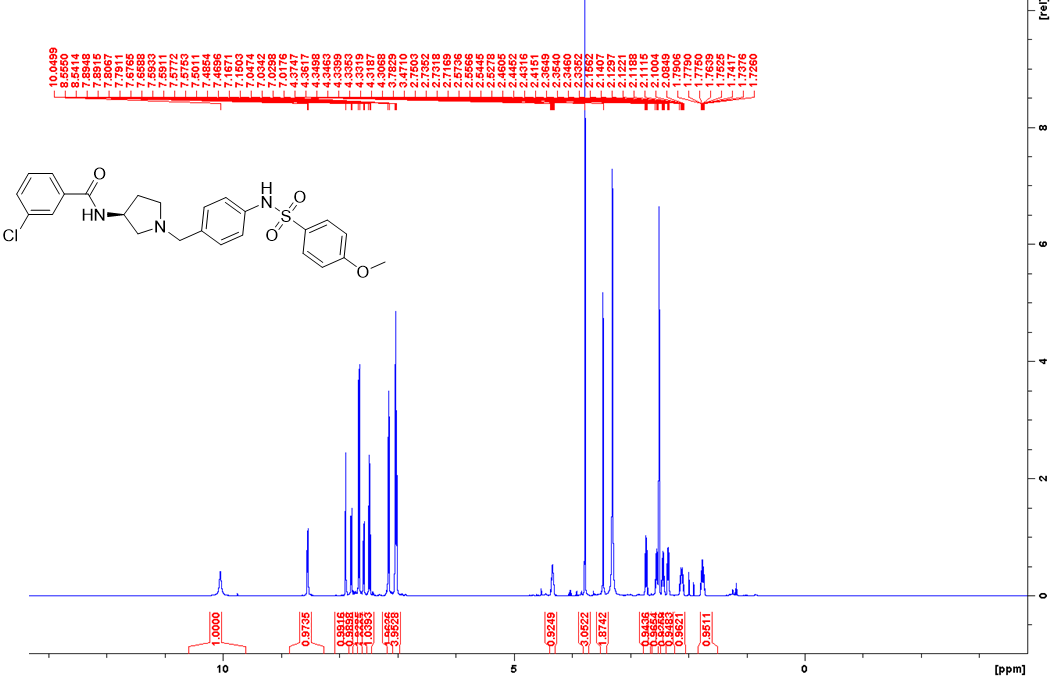


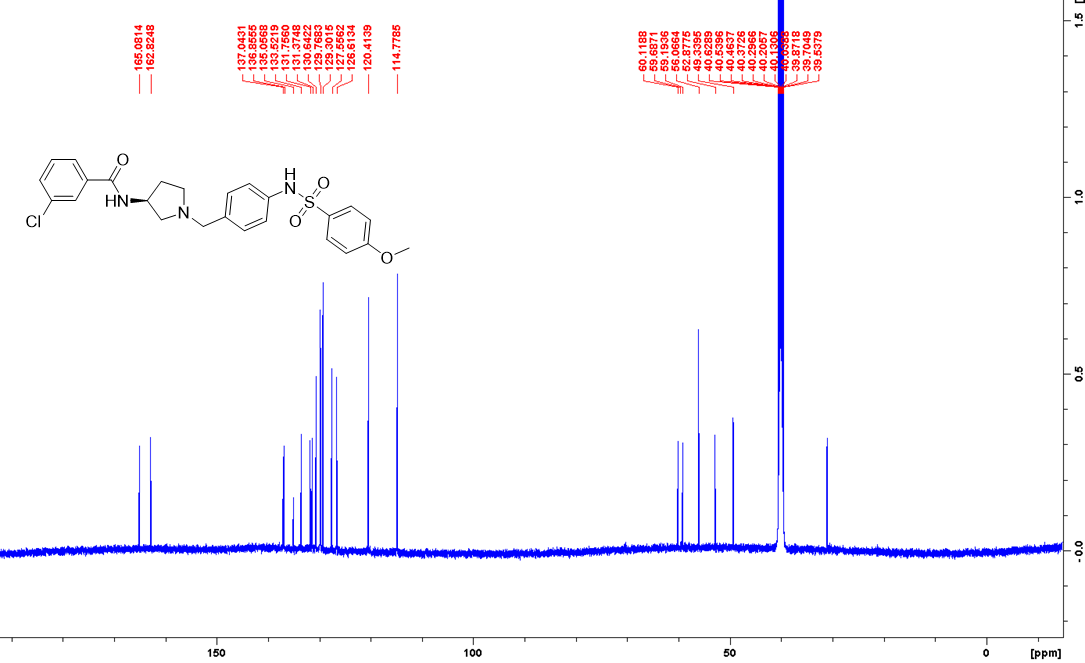


**
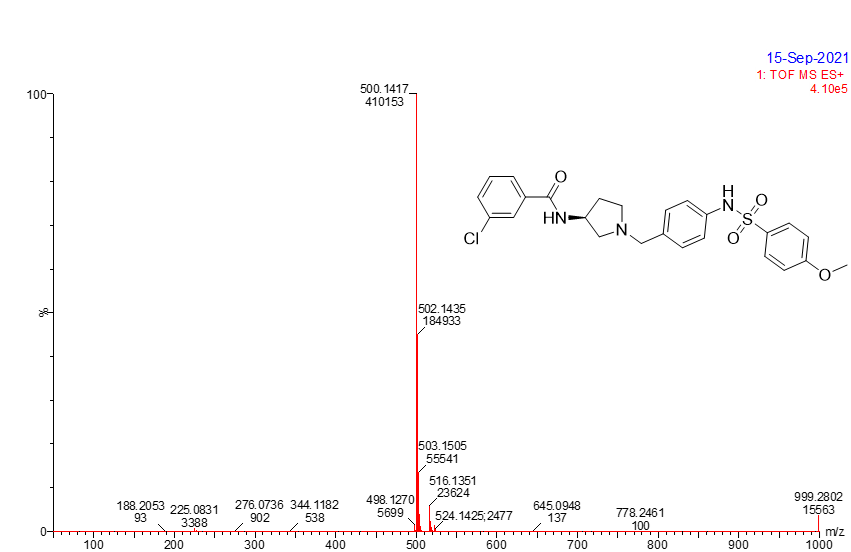
**

**^1^H, ^13^C NMR and HRMS spectra of compound 31**


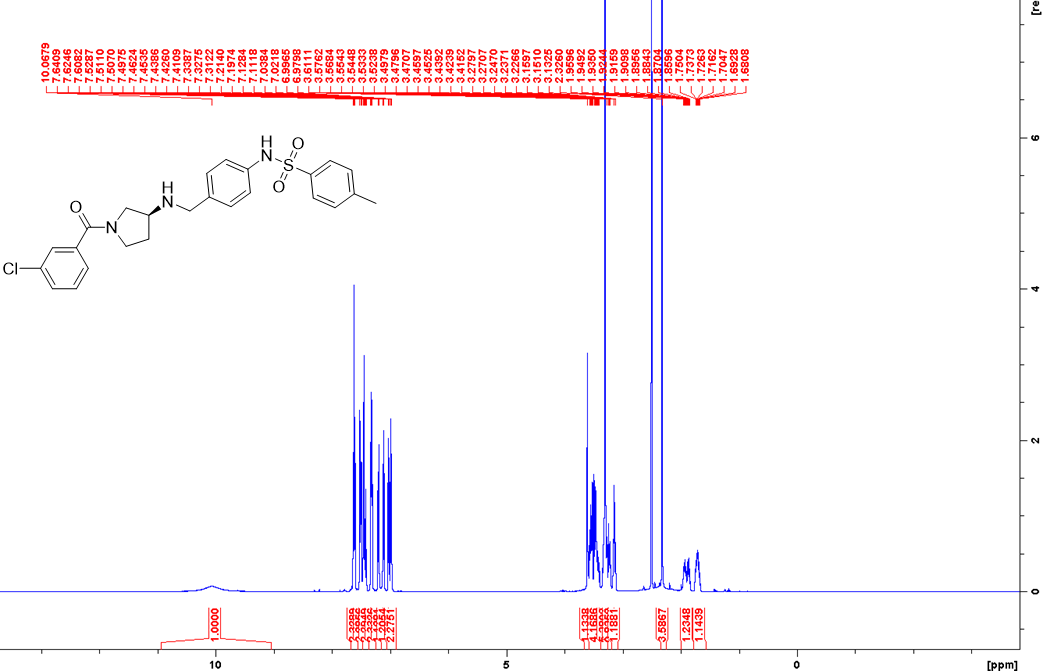


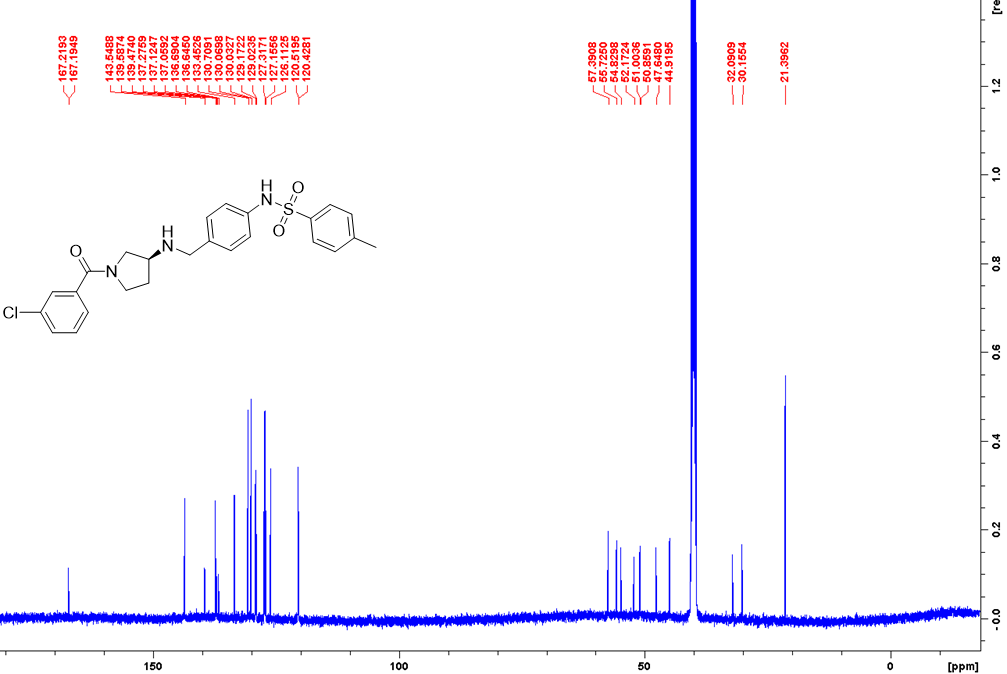


**
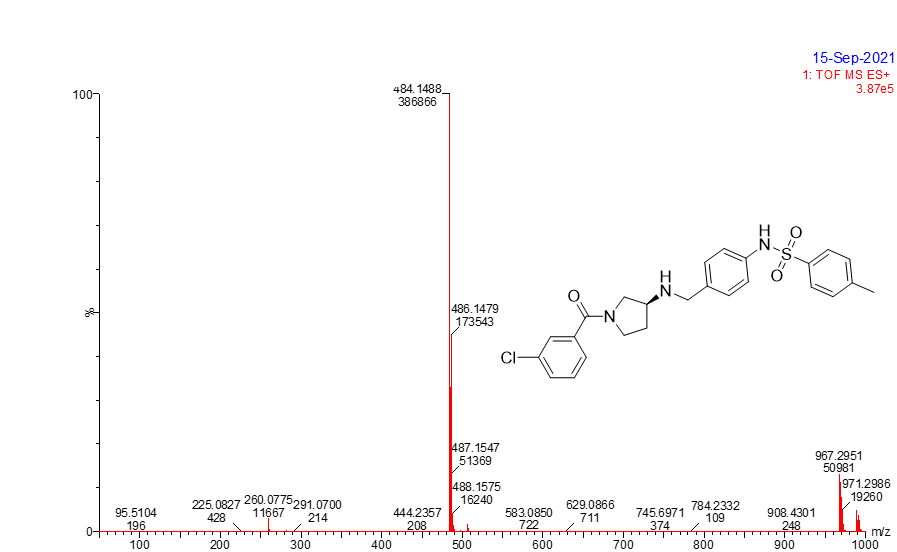
**

**^1^H, ^13^C NMR and HRMS spectra of compound 32**

**
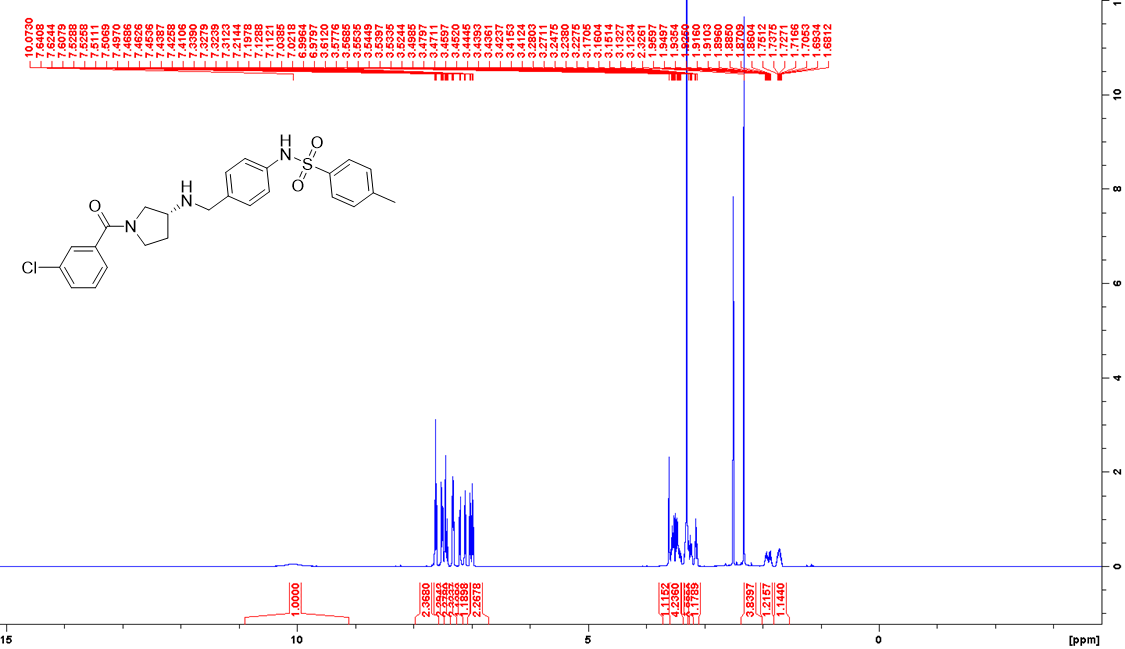
**


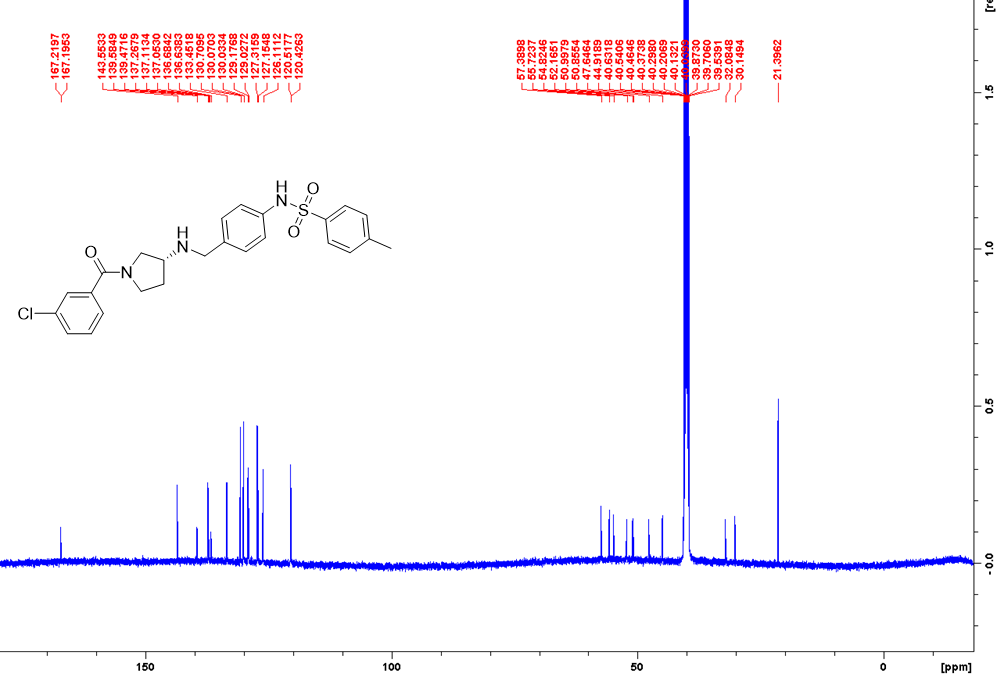


**
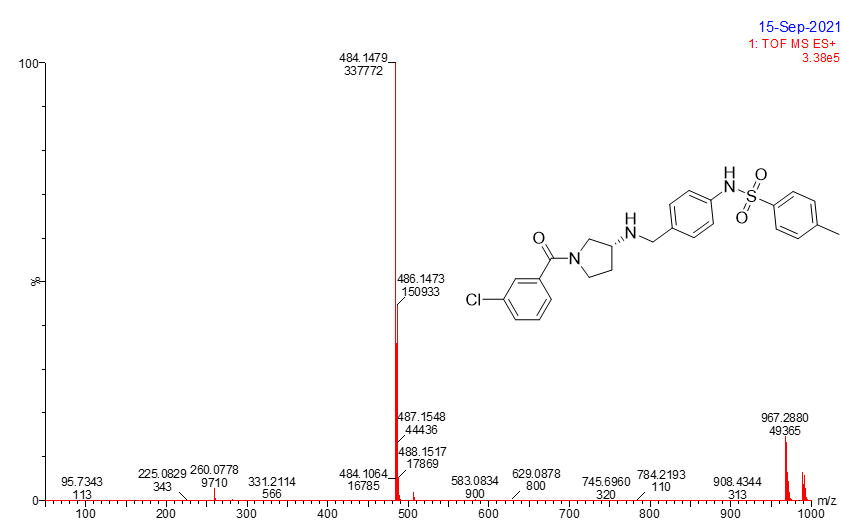
**

**^1^H, ^13^C NMR and HRMS spectra of compound 33**

**
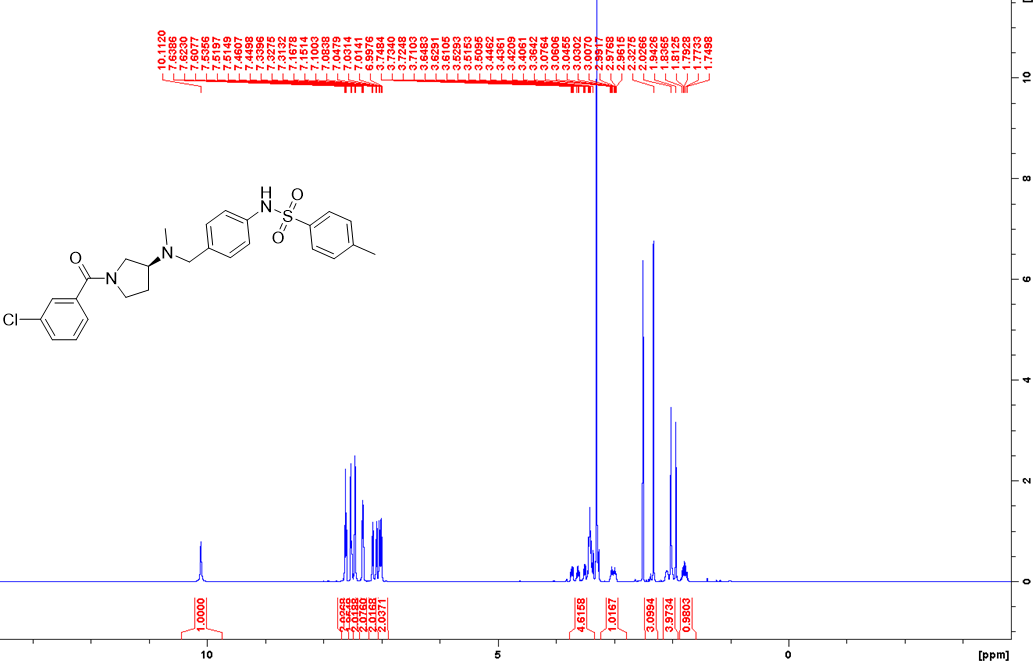
**

**
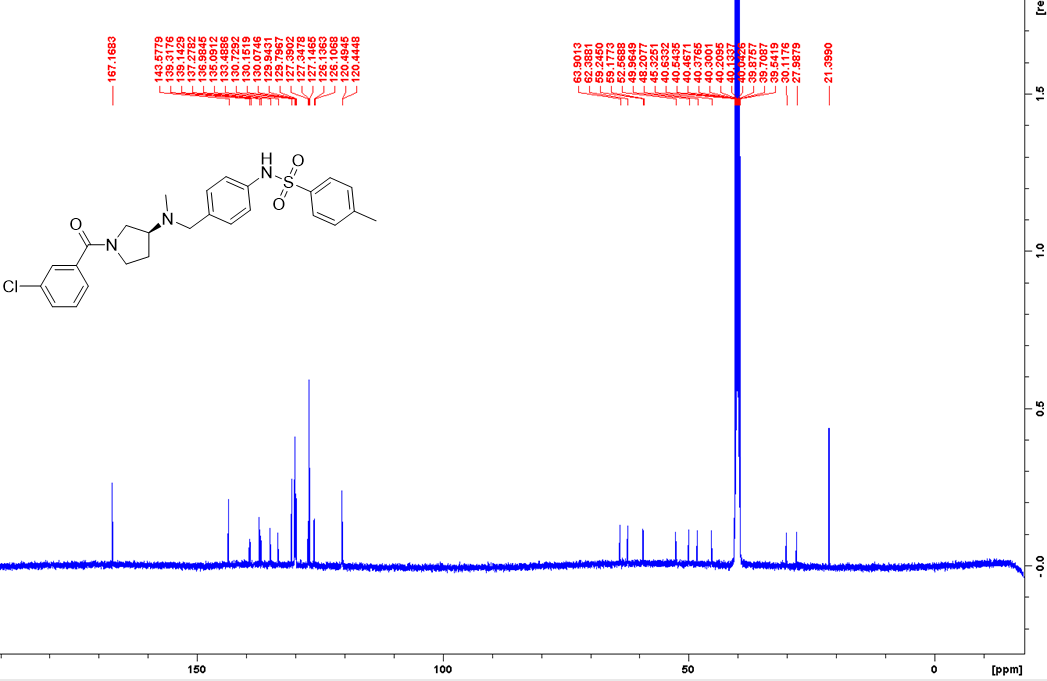
**

**
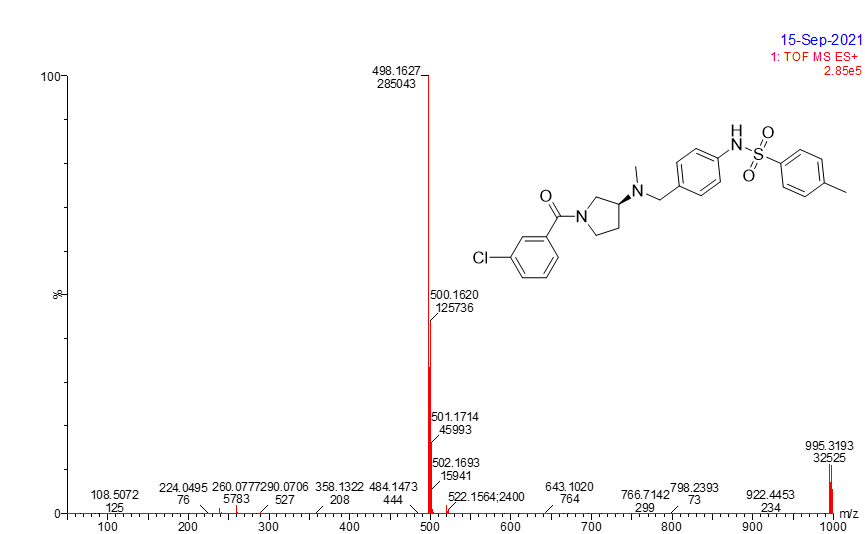
**

**^1^H, ^13^C NMR and HRMS spectra of compound 34**

**
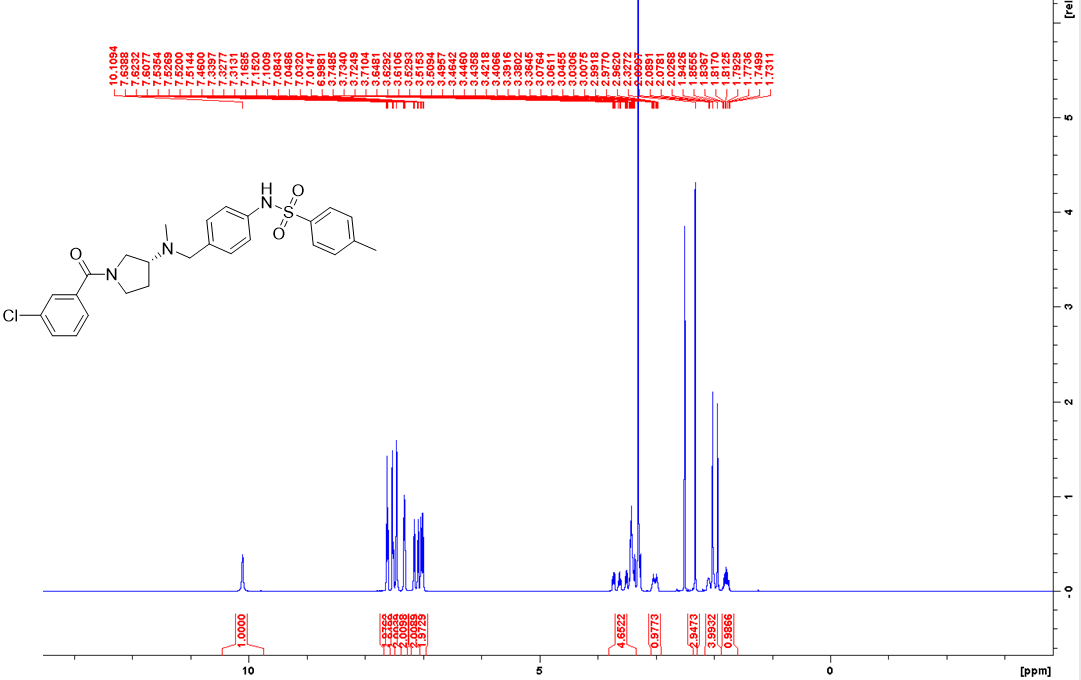
**

**^
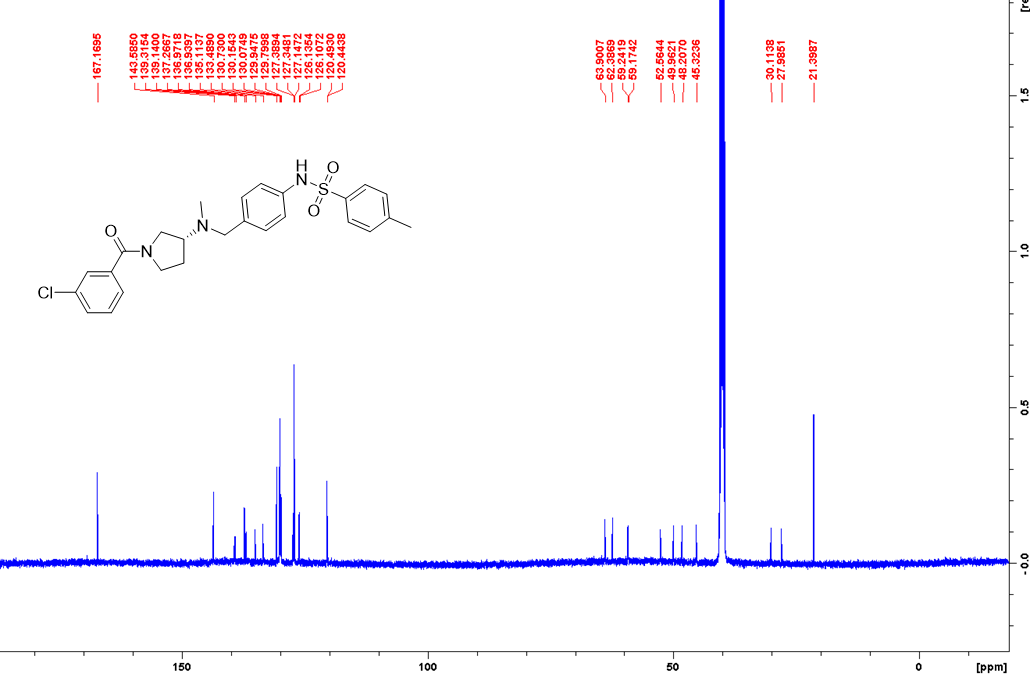
^**

**^
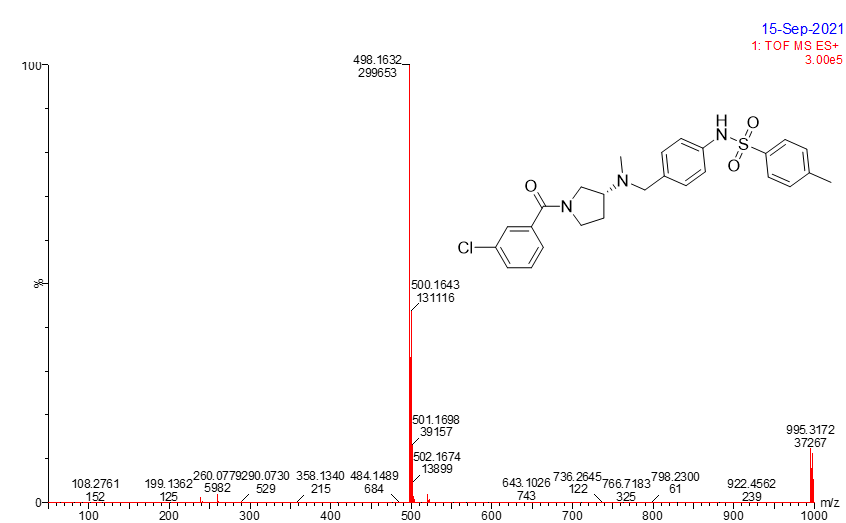
^**

**^1^H, ^13^C NMR and HRMS spectra of compound 35**


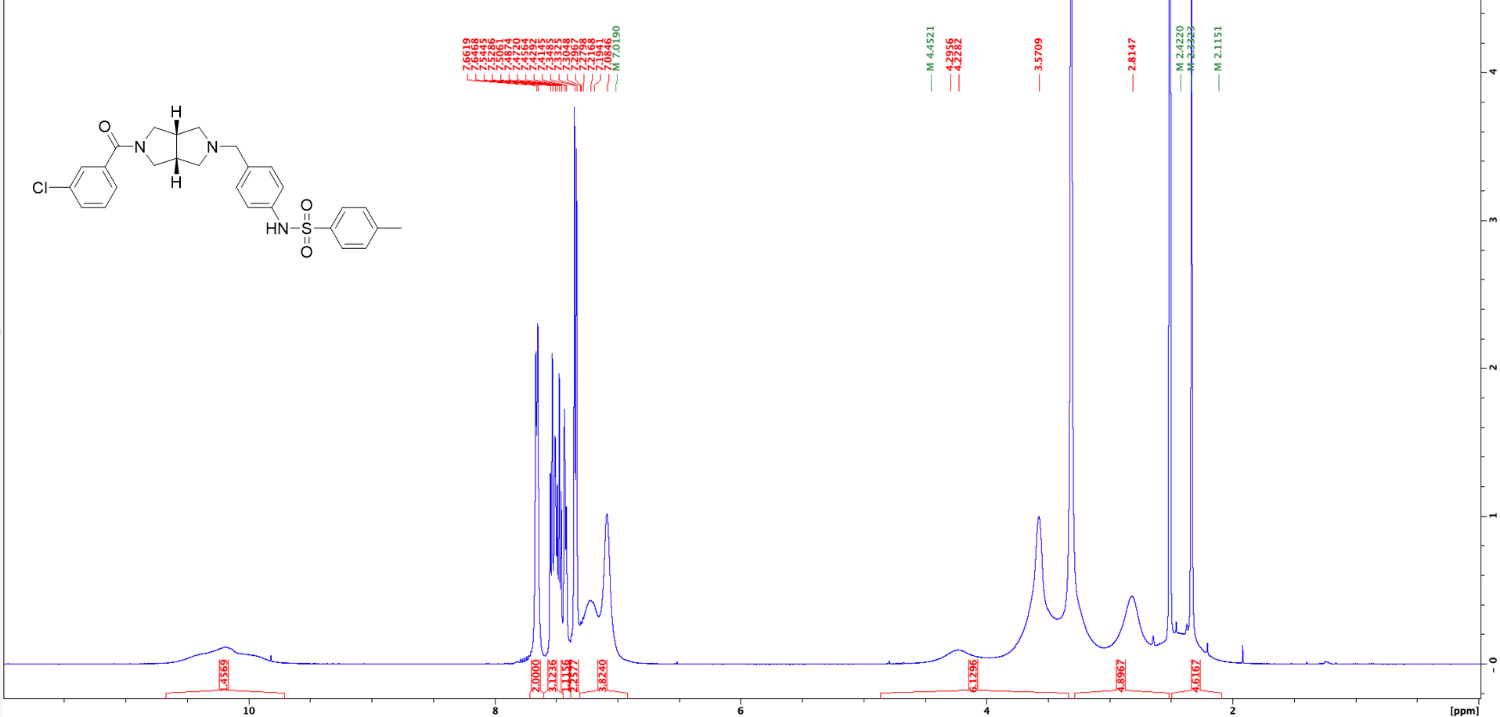


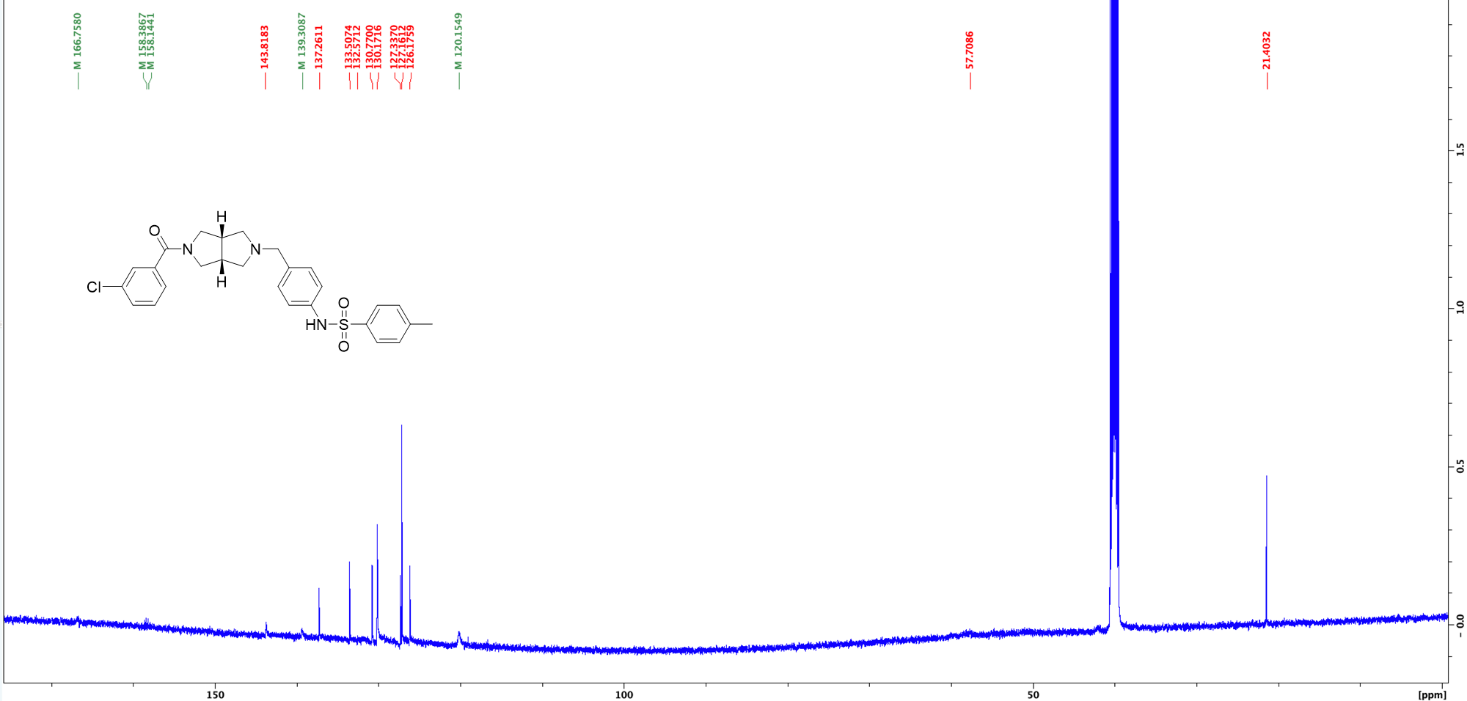


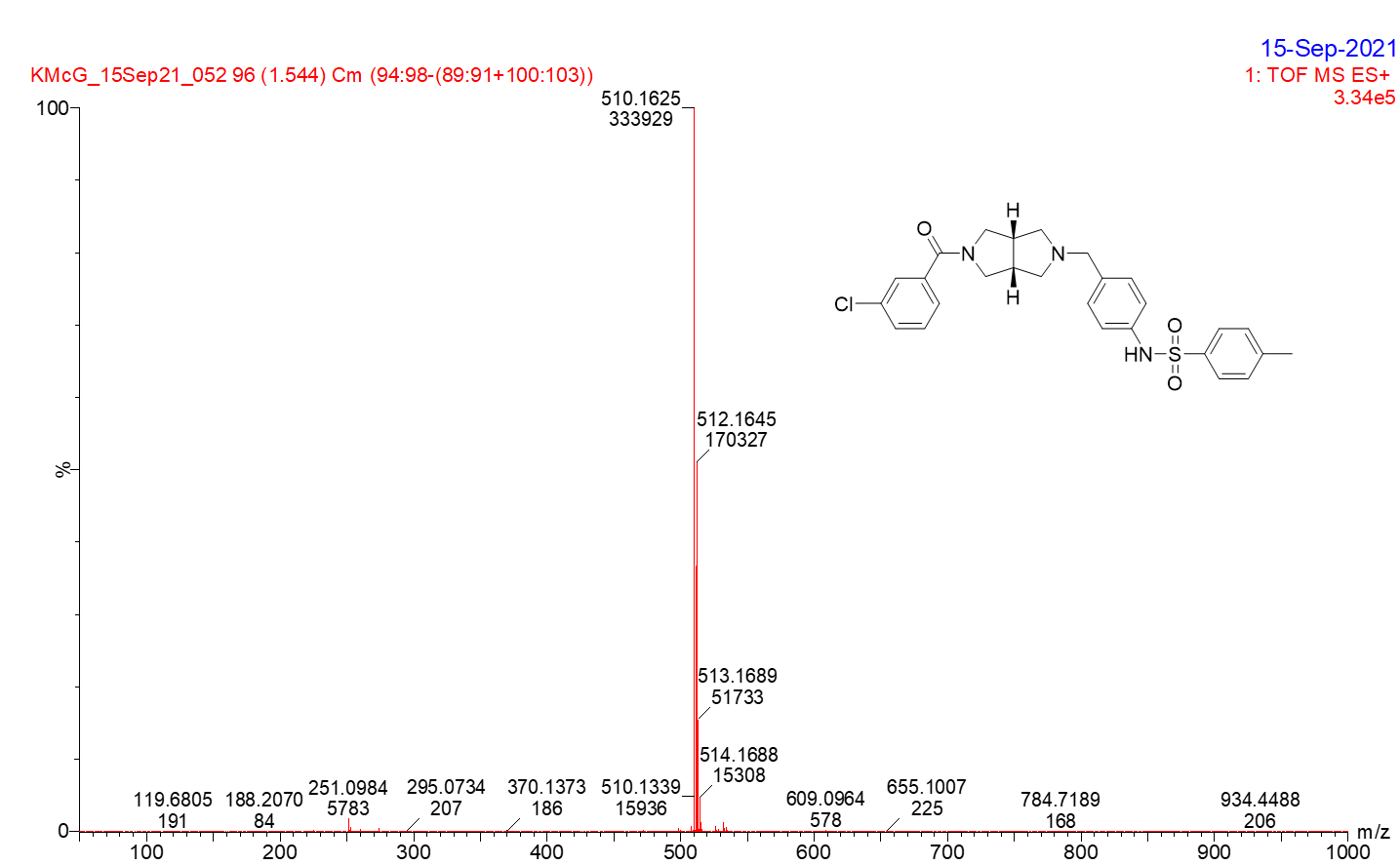


**^1^H, ^13^C NMR and HRMS spectra of compound 36**

**
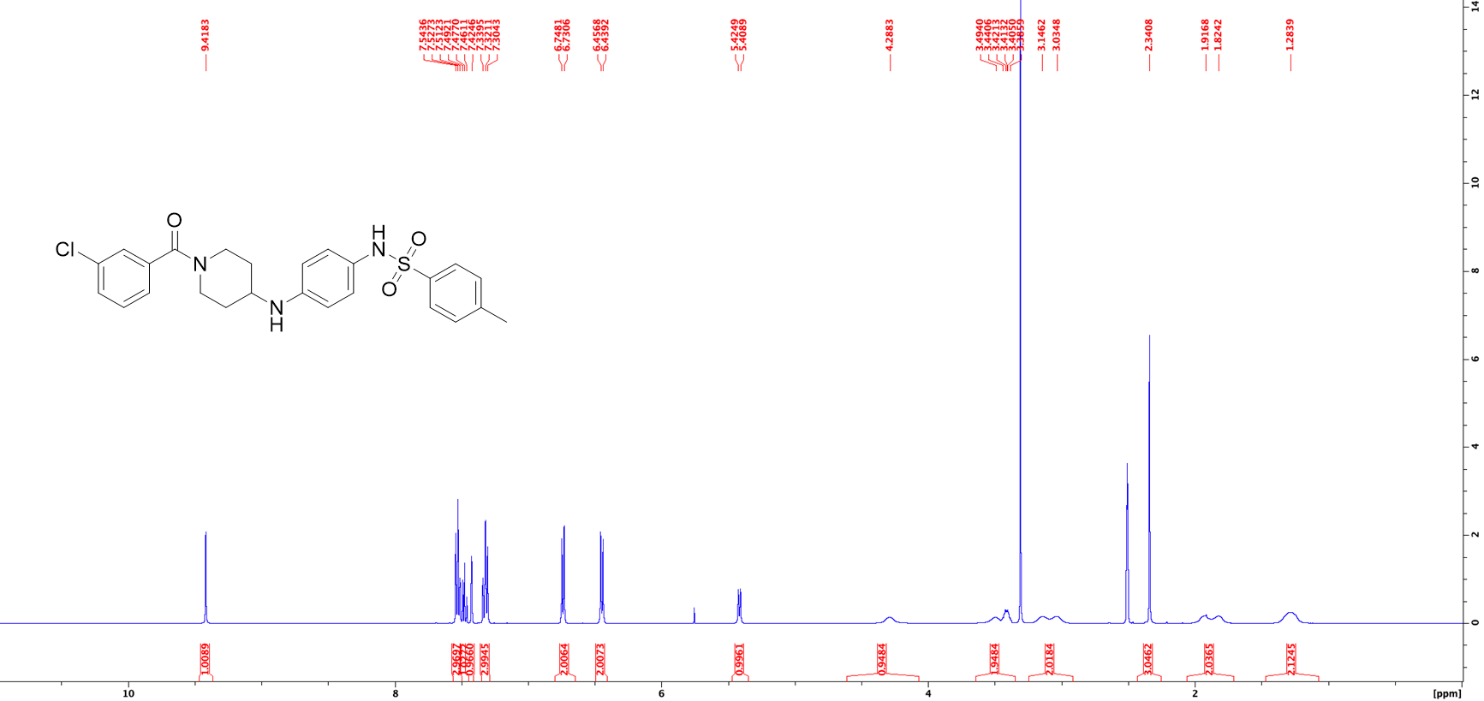
**

**
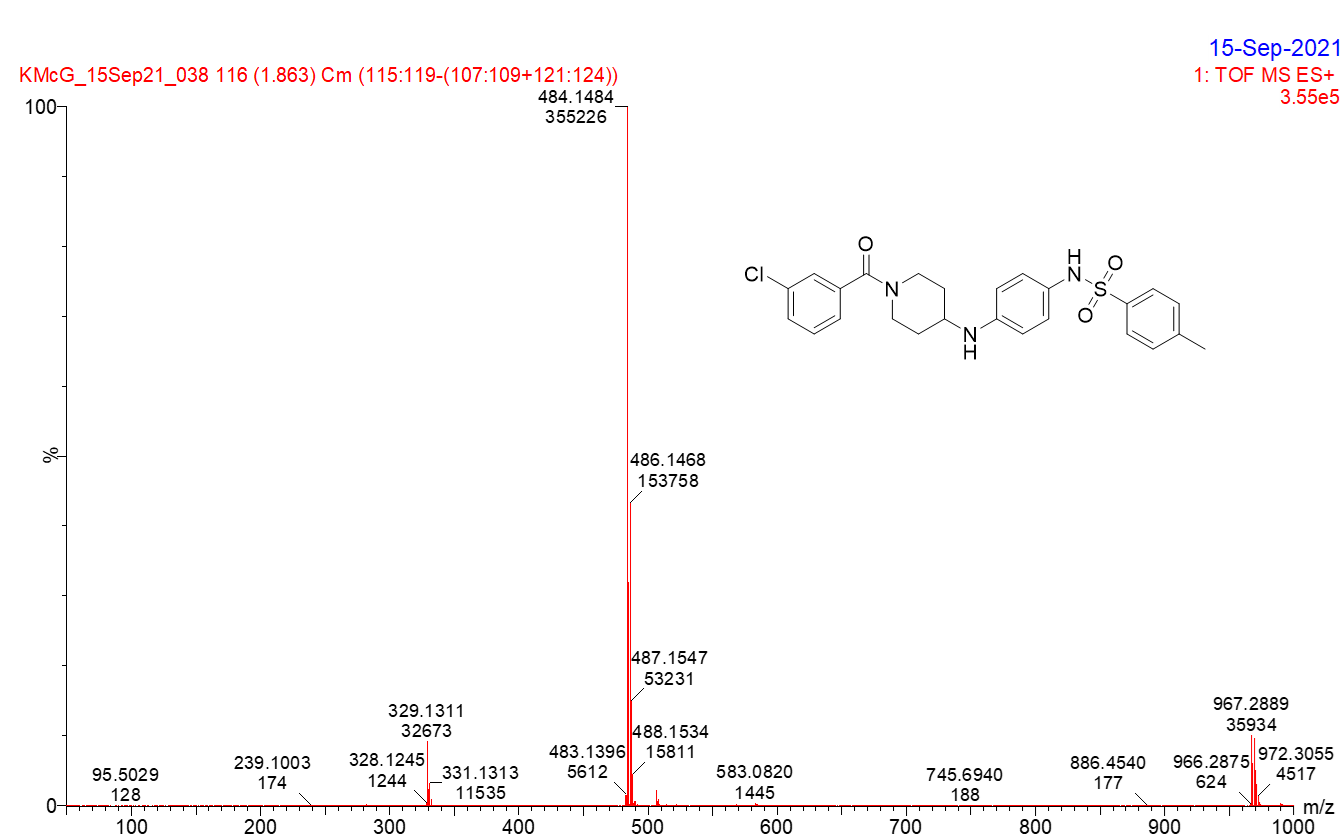
**

**^1^H, ^13^C NMR and HRMS spectra of compound 37**

**
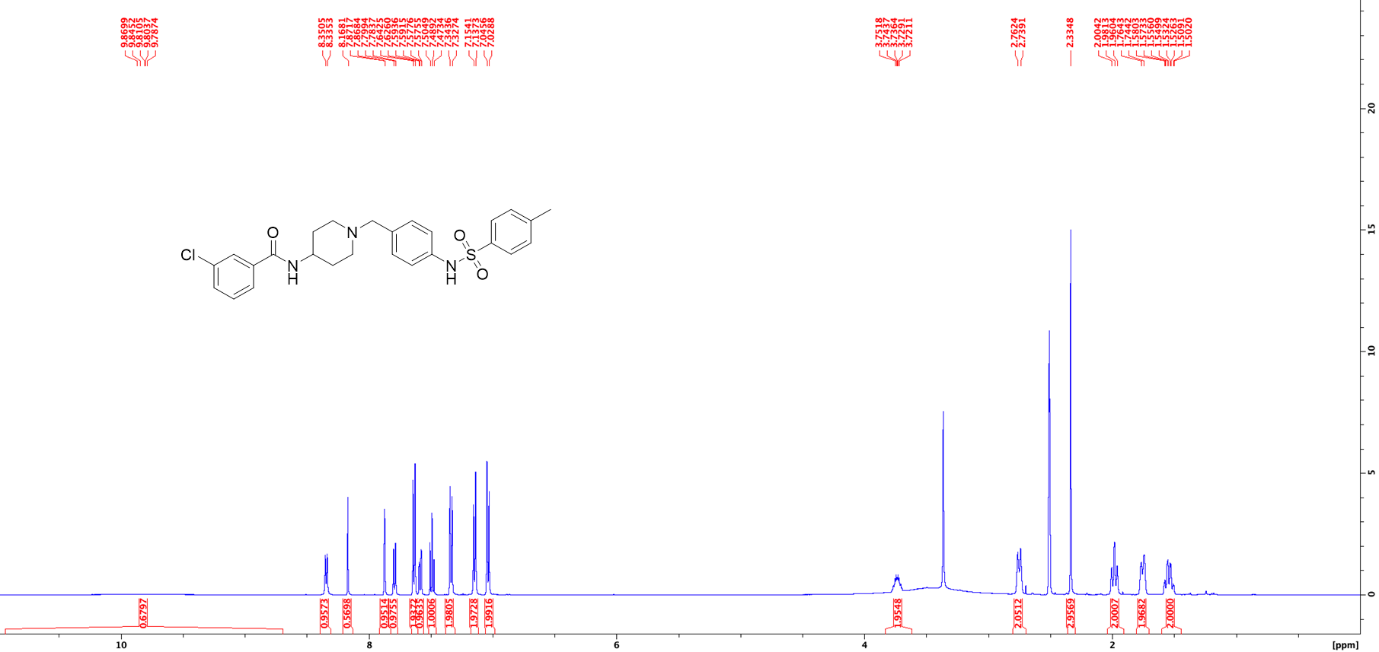
**

**
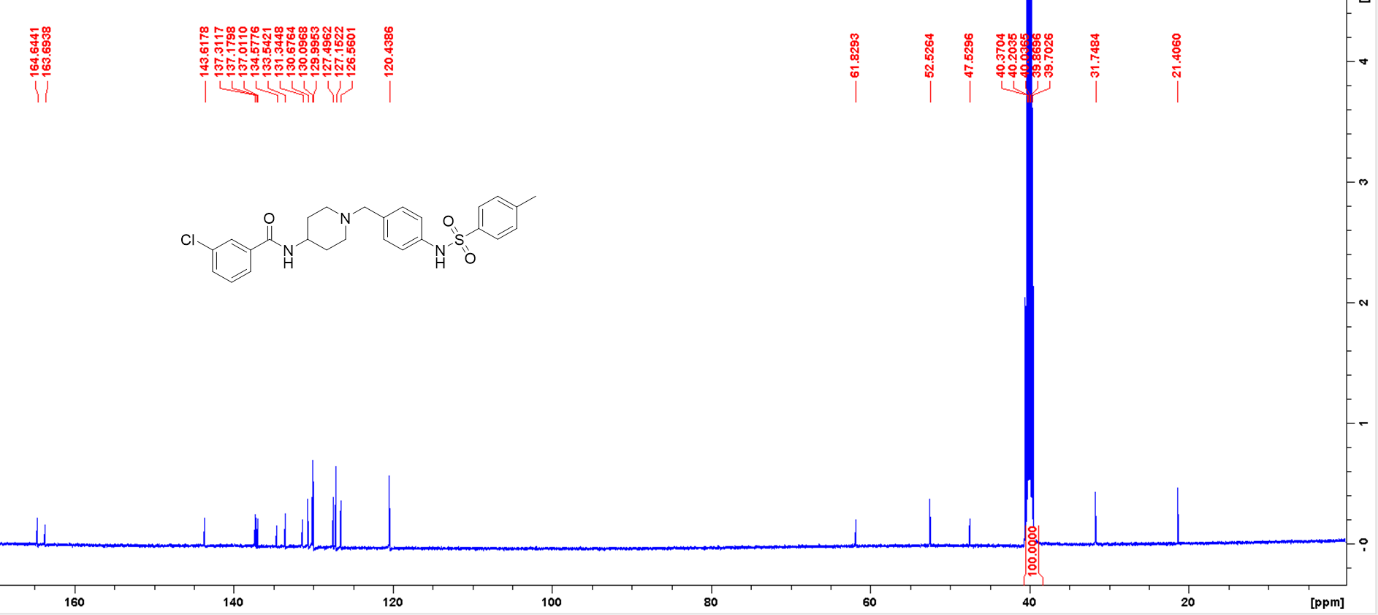
**

**
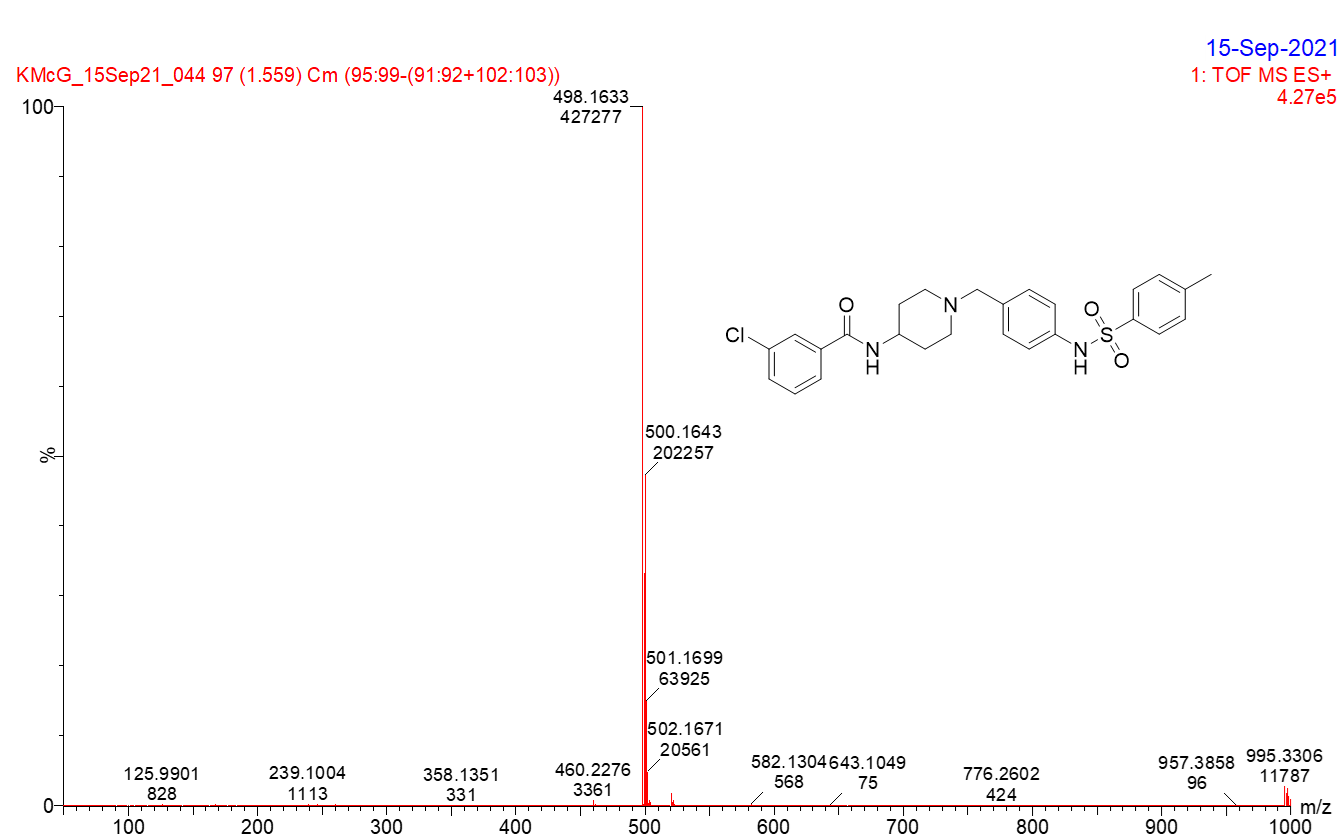
**

**^1^H, ^13^C NMR and HRMS spectra of compound 38**

**
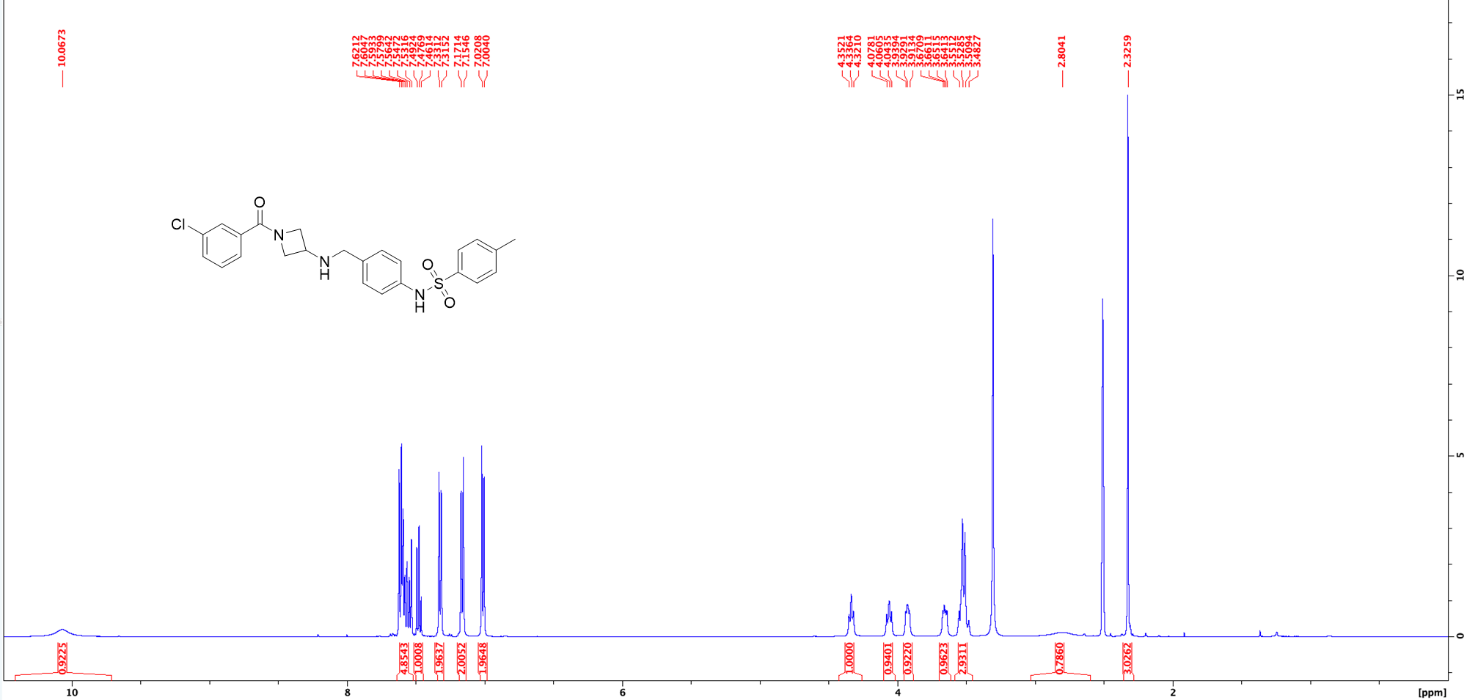
**

**^1^H, ^13^C NMR and HRMS spectra of compound 39**

**^1^H, ^13^C NMR and HRMS spectra of compound 40**

**^1^H, ^13^C NMR and HRMS spectra of compound 41**

**^1^H, ^13^C NMR and HRMS spectra of compound 42**

**^1^H, ^13^C NMR and HRMS spectra of compound 43**

**^1^H, ^13^C NMR and HRMS spectra of compound 44**

**^1^H, ^13^C NMR and HRMS spectra of compound 45**

**^1^H NMR spectrum of compound 46**

**7. References**

1. De Rycker M.; Thomas J.; Riley J.; Brough S.J.; Miles T. J.; Gray D.W. [**Identification of Trypanocidal Activity for Known Clinical Compounds Using a New *Trypanosoma cruzi* Hit-Discovery Screening Cascade**](https://journals.plos.org/plosntds/article?id=10.1371/journal.pntd.0004584)**.** *PLoS Negl. Trop. Dis*. **2016**, 10(4): e0004584
2. Svensen N.; Wyllie S.; Gray D. W.; De Rycker M. Live-imaging rate-of-kill compound profiling for Chagas disease drug discovery with a new automated high-content assay. *PLoS Negl. Trop. Dis*. **2021**, 15(10): e0009870
3. Riley J.; Brand S; Voice M.; Caballero I.; Calvo D.; Read K. D. Development of a Fluorescence-based Trypanosoma cruzi CYP51 Inhibition Assay for Effective Compound Triaging in Drug Discovery Programmes for Chagas Disease. *PLoS Negl. Trop. Dis*. **2015**, 9(9): e0004014
4. Robinson M. W.; Hill A. P.; Readshaw S. A.; Hollerton J. C.; Upton R. J.; Lynn S. M.; Besley S. C.; Boughtflower B. J. Use of Calculated Physicochemical Properties to Enhance Quantitative Response When Using Charged Aerosol Detection *Anal. Chem.* **2017**, 89(3), 1772–1777
5. Brand, S.; Ko, E. J.; Viayna, E.; Thompson, S.; Spinks, D.; Thomas, M.; Sandberg, L.; Francisco, A. F.; Jayawardhana, S.; Smith, V. C.; Jansen, C.; De Rycker, M.; Thomas, J.; MacLean, L.; Osuna-Cabello, M.; Riley, J.; Scullion, P.; Stojanovski, L.; Simeons, F. R. C.; Epemolu, O.; Shishikura, Y.; Crouch, S. D.; Bakshi, T. S.; Nixon, C. J.; Reid, I. H.; Hill, A. P.; Underwood, T. Z.; Hindley, S. J.; Robinson, S. A.; Kelly, J. M.; Fiandor, J. M.; Wyatt, P. G.; Marco, M.; Miles, T. J.; Read, K. D.; Gilbert, I. H. Discovery and Optimization of 5-Amino-1,2,3-triazole-4-carboxamide Series against Trypanosoma cruzi. *J. Med. Chem.* **2017**, 60, 7284-7299.
6. Lewis M. D.; Fortes Francisco A.; Taylor M. C.; Kelly J. M. A New Experimental Model for Assessing Drug Efficacy against *Trypanosoma cruzi* Infection Based on Highly Sensitive In Vivo Imaging *Journal of Biomolecular Screening* **2015**, 20(1), 36–43
